# Supplementary figures and images for: The BAF53A-BACH1-GCLM axis regulates glutathione metabolism and enhances ferroptosis resistance in esophageal squamous cell carcinoma
Source: PeerJ. 2025 Oct 3;13:e20156. doi: 10.7717/peerj.20156 (PMC12499559; doi:10.7717/peerj.20156)

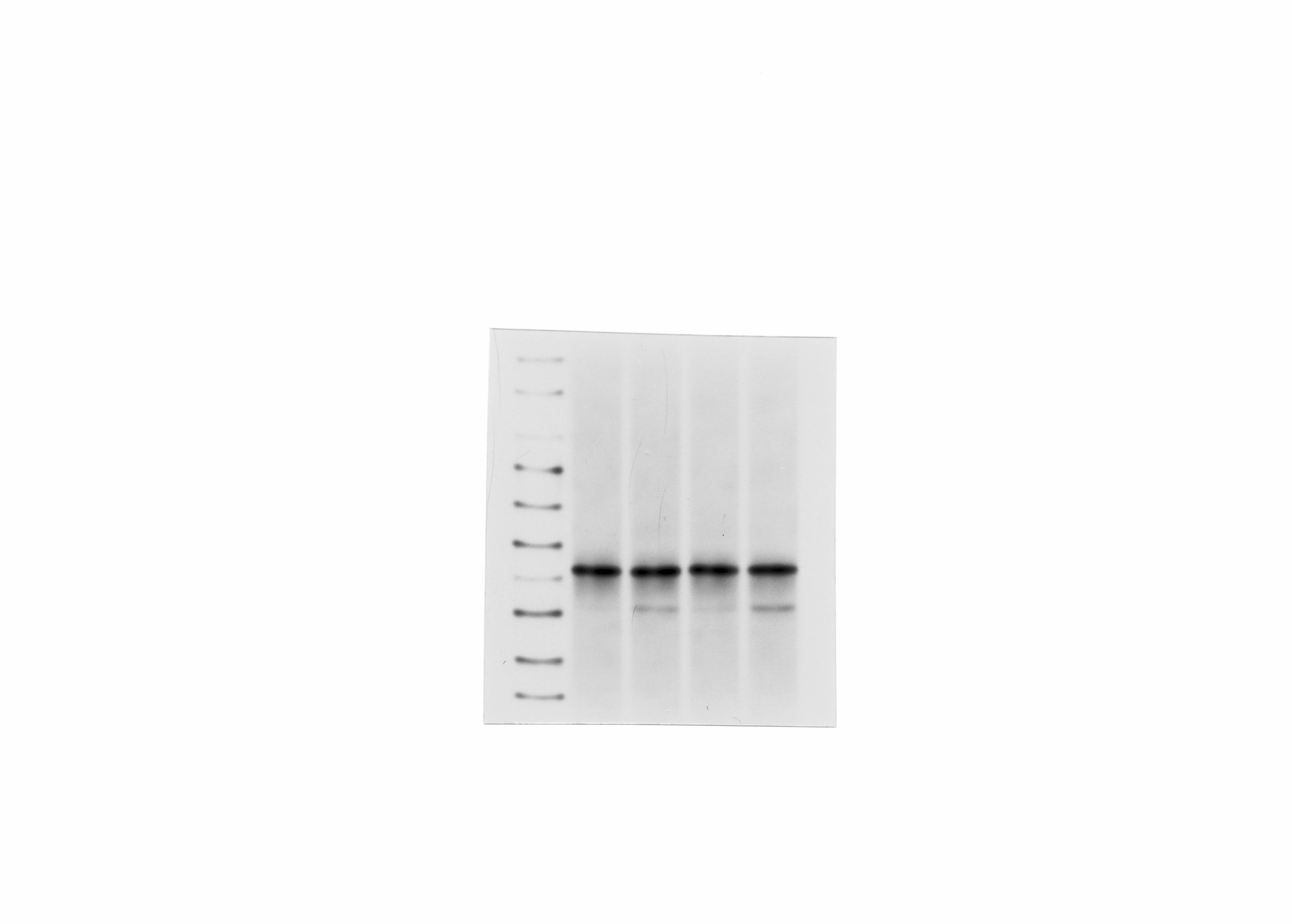

Supplement: Supplemental Information 1 [file peerj-13-20156-s001.zip › 6B-2/ACTB.tif]

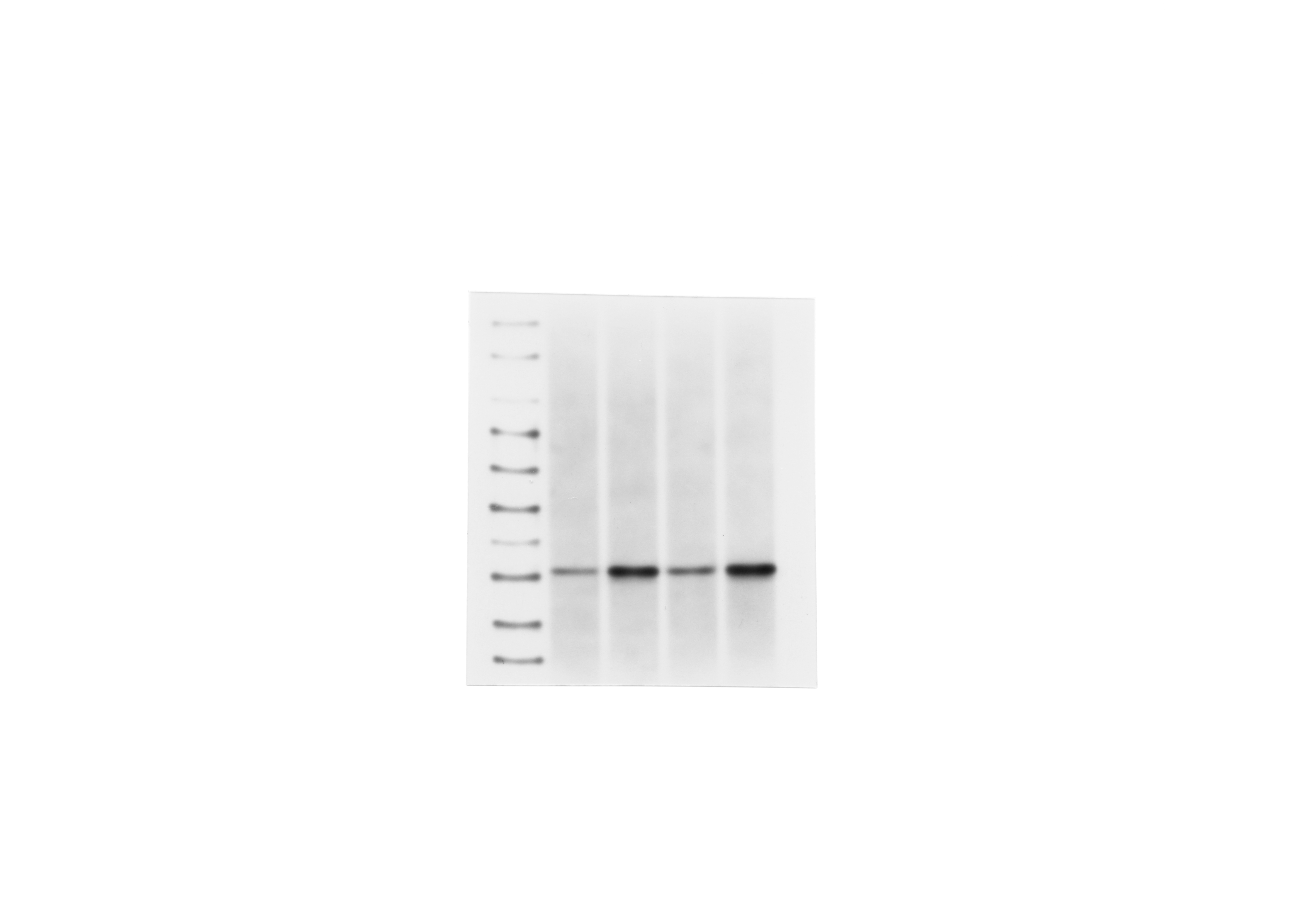

Supplement: Supplemental Information 1 [file peerj-13-20156-s001.zip › 6B-2/GCLM.tif]

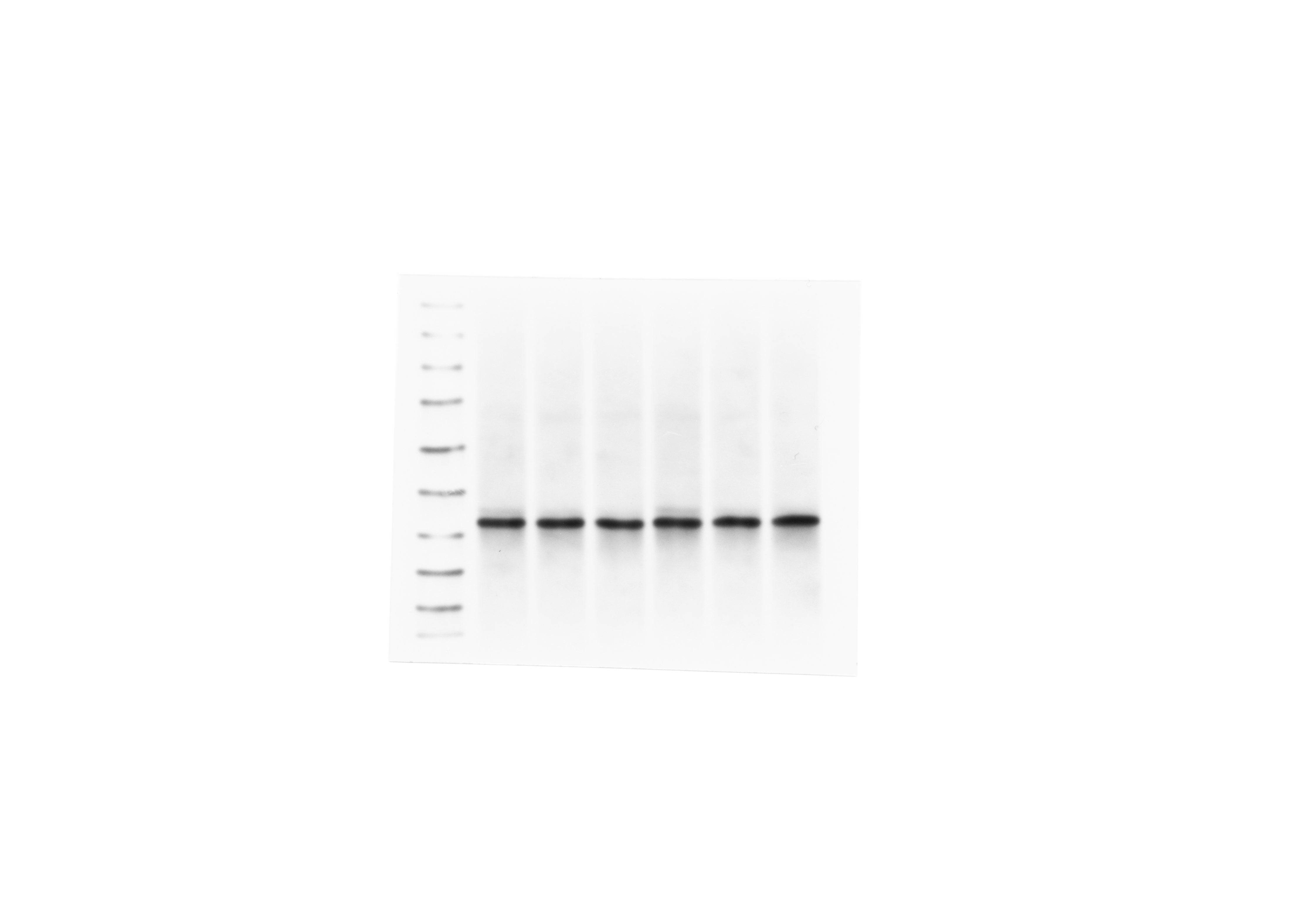

Supplement: Supplemental Information 1 [file peerj-13-20156-s001.zip › 1D/ACTB.tif]

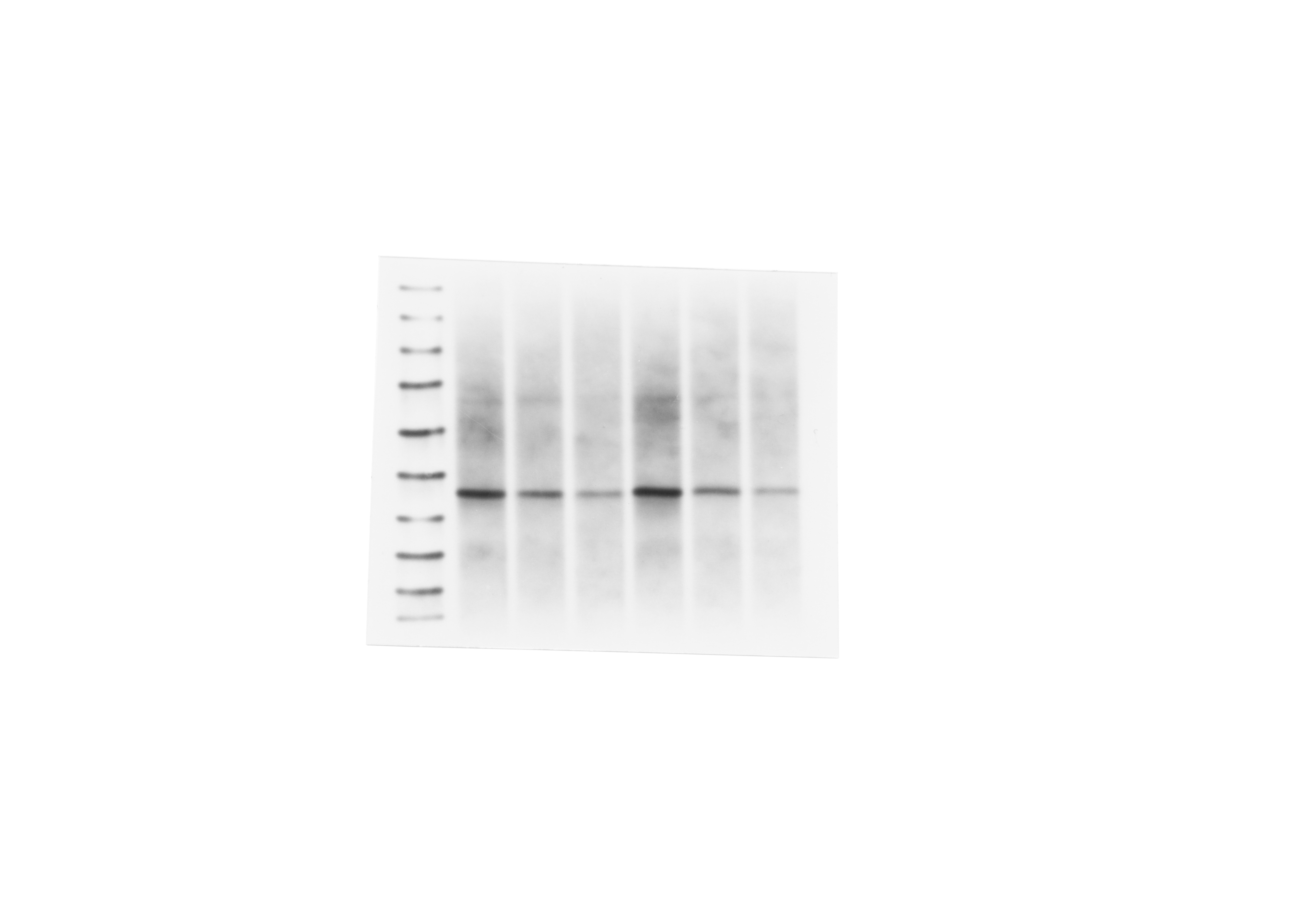

Supplement: Supplemental Information 1 [file peerj-13-20156-s001.zip › 1D/BAF53A.tif]

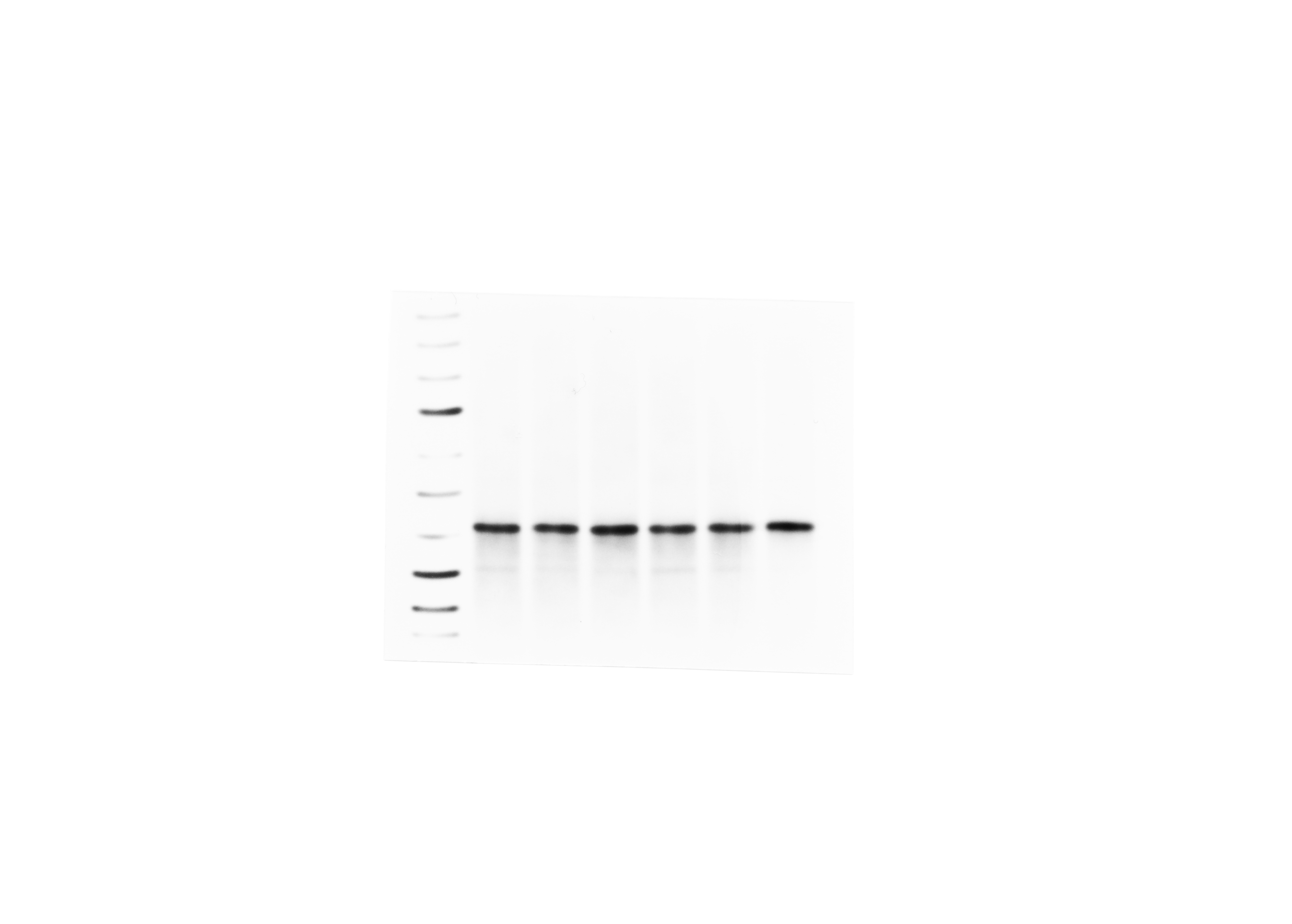

Supplement: Supplemental Information 1 [file peerj-13-20156-s001.zip › 4B/ACTB.tif]

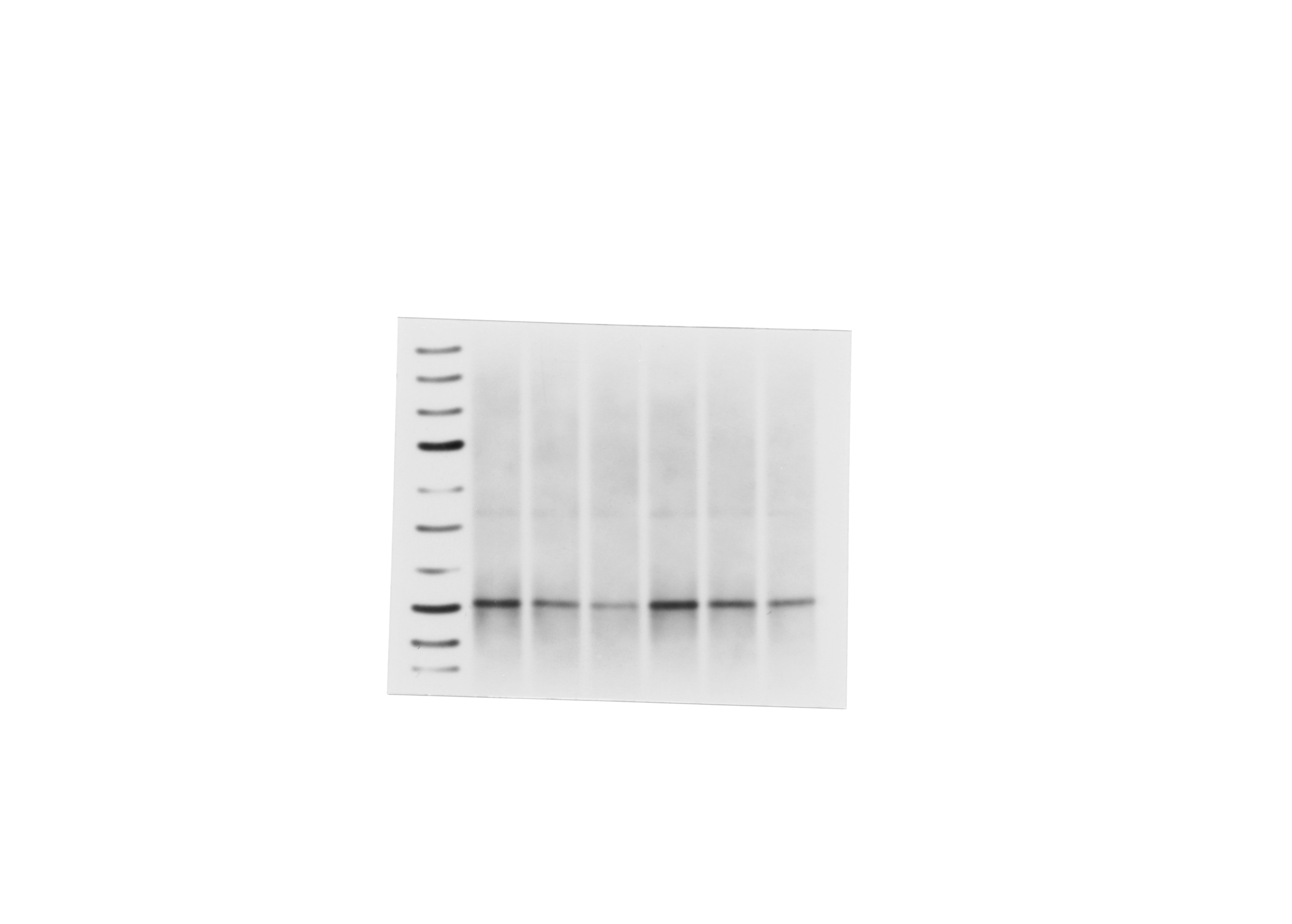

Supplement: Supplemental Information 1 [file peerj-13-20156-s001.zip › 4B/GCLM.tif]

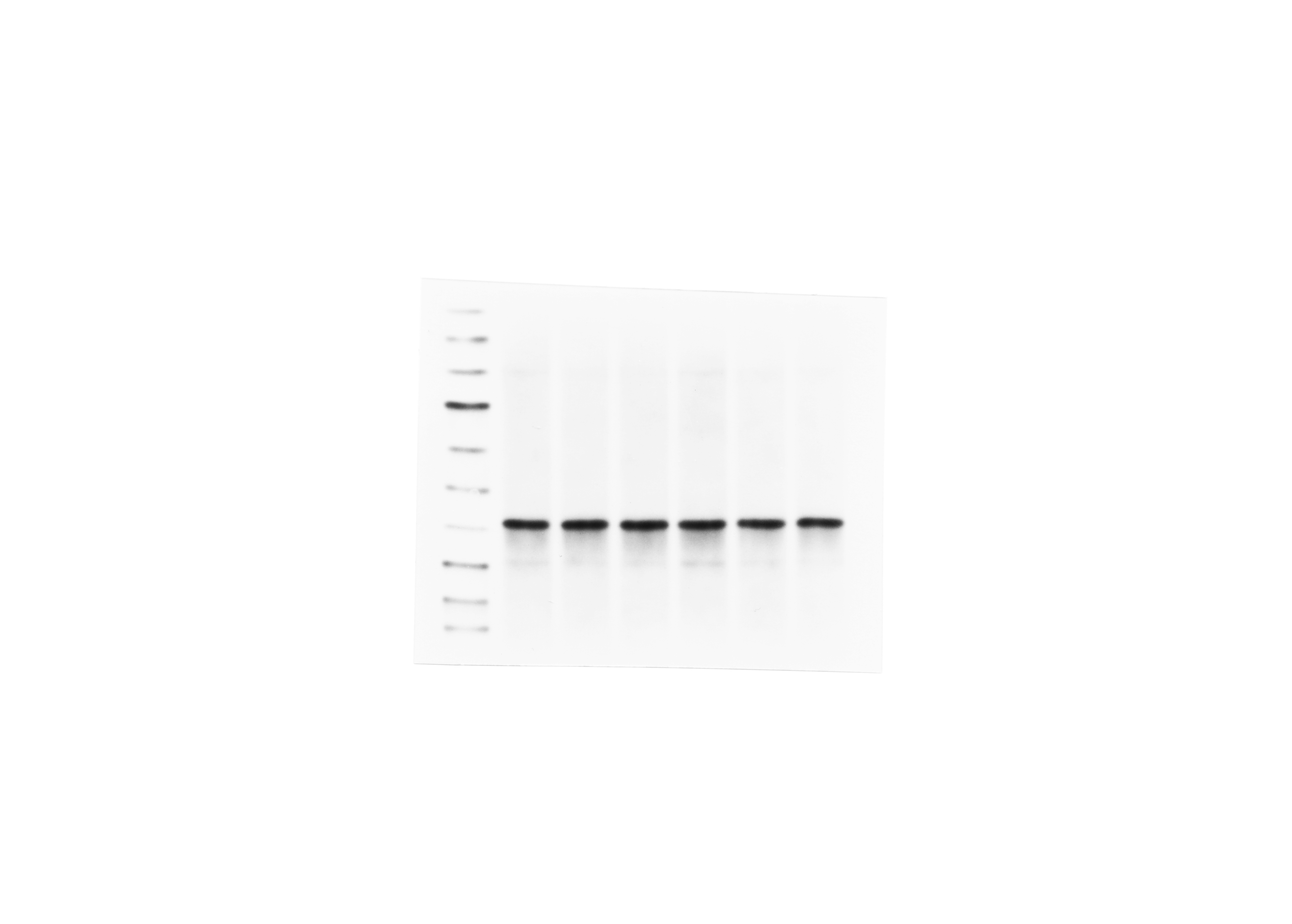

Supplement: Supplemental Information 1 [file peerj-13-20156-s001.zip › 4F/ACTB.tif]

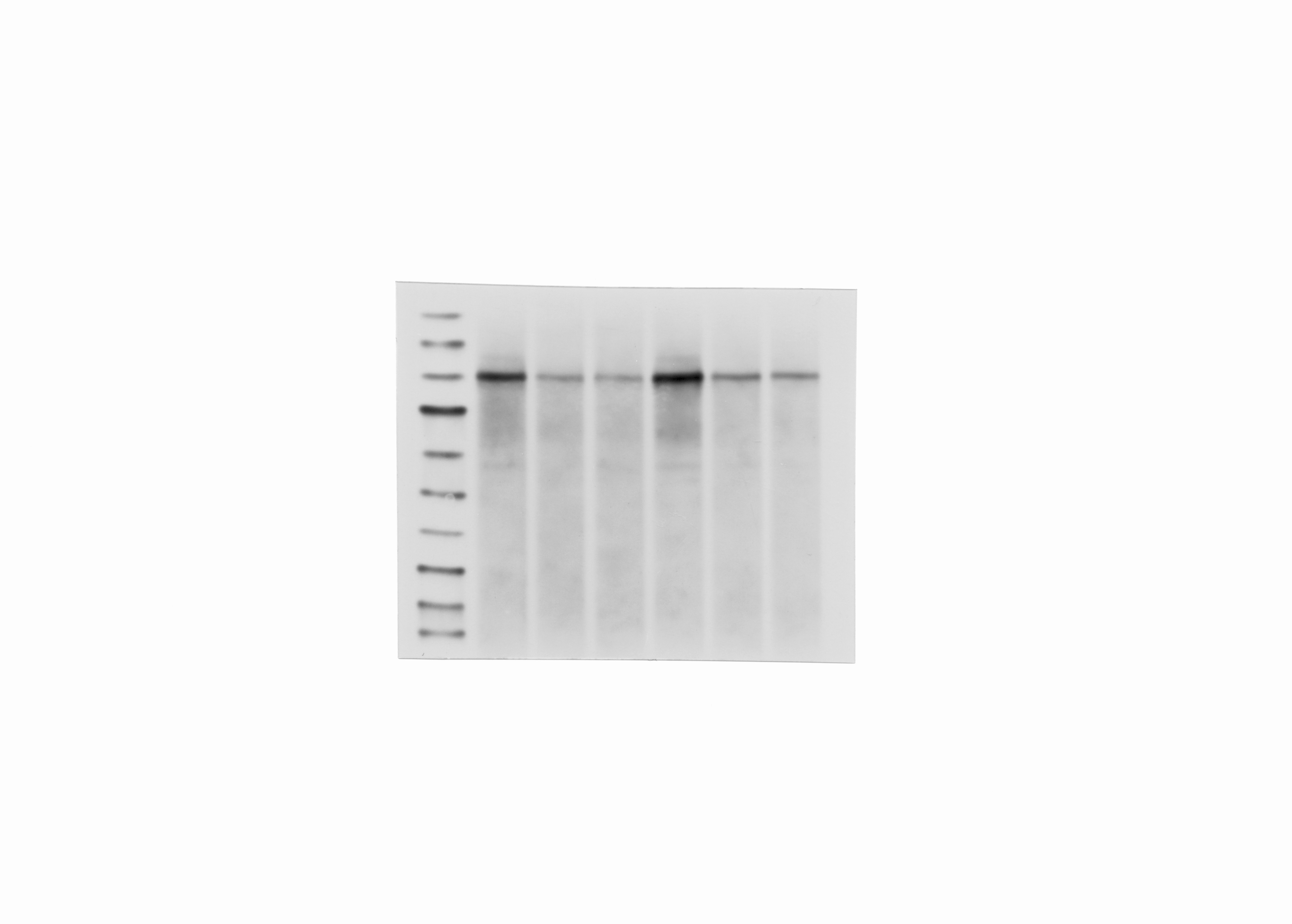

Supplement: Supplemental Information 1 [file peerj-13-20156-s001.zip › 4F/BACH1.tif]

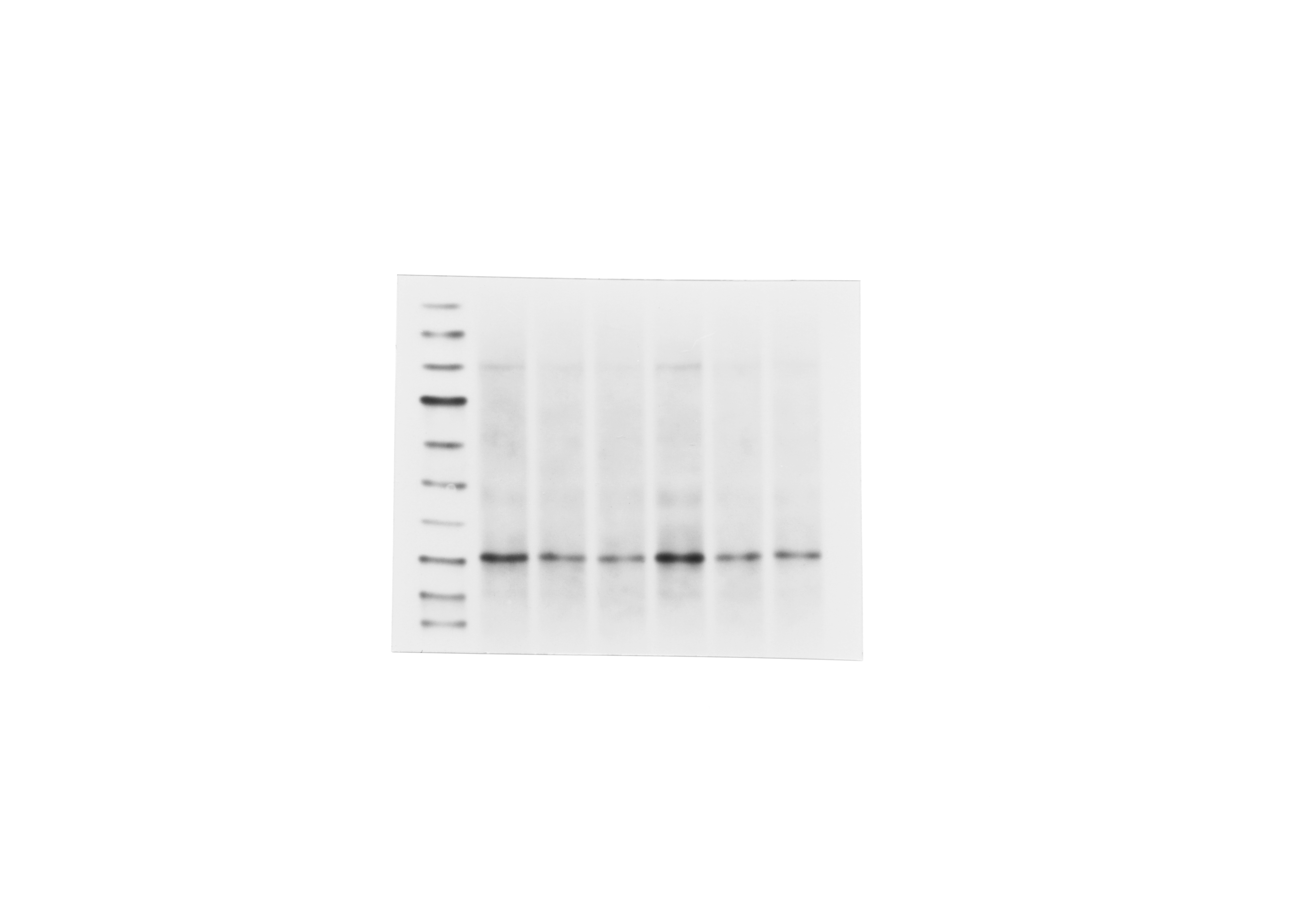

Supplement: Supplemental Information 1 [file peerj-13-20156-s001.zip › 4F/GCLM.tif]

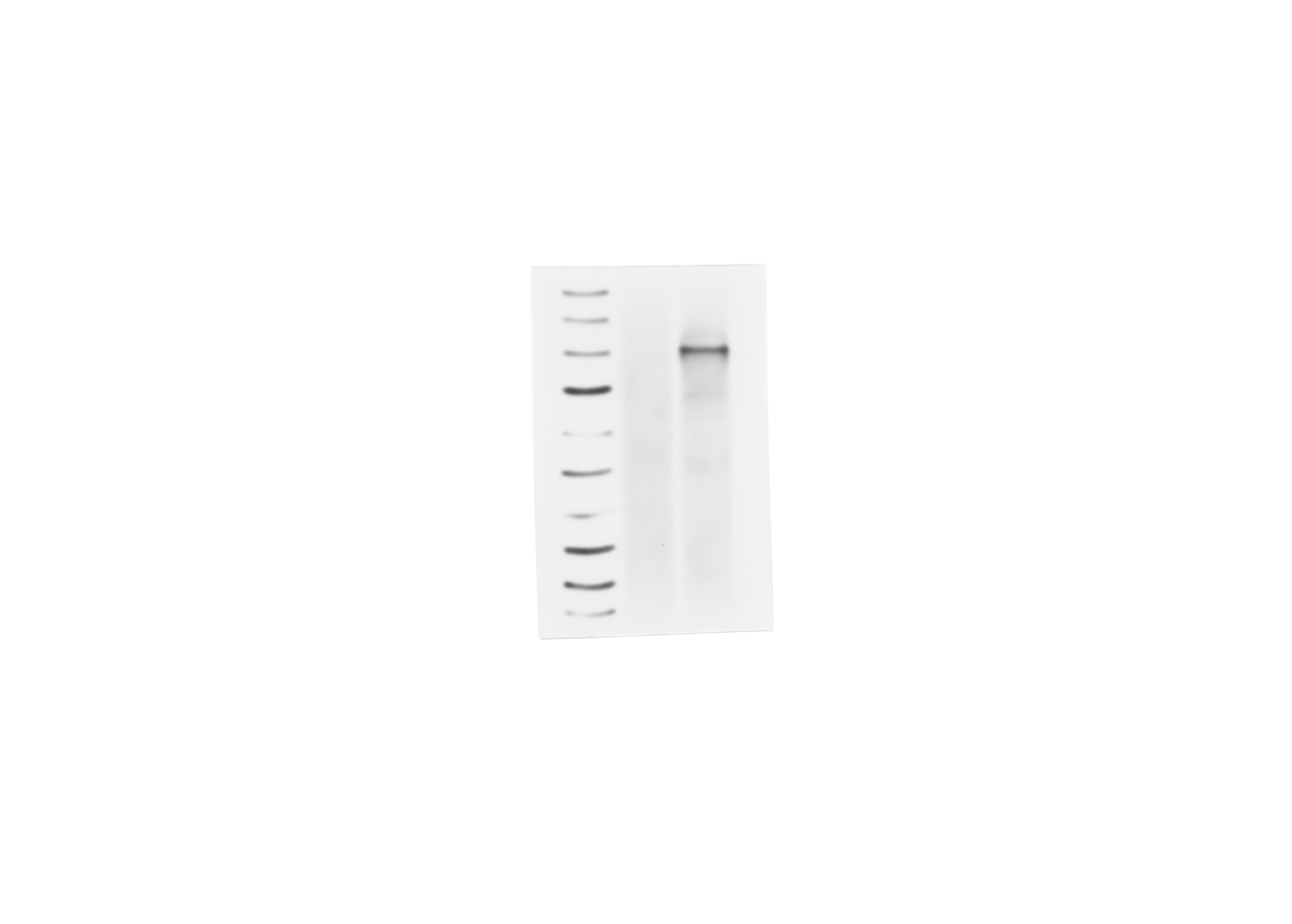

Supplement: Supplemental Information 1 [file peerj-13-20156-s001.zip › 4I-1/BACH1-1.tif]

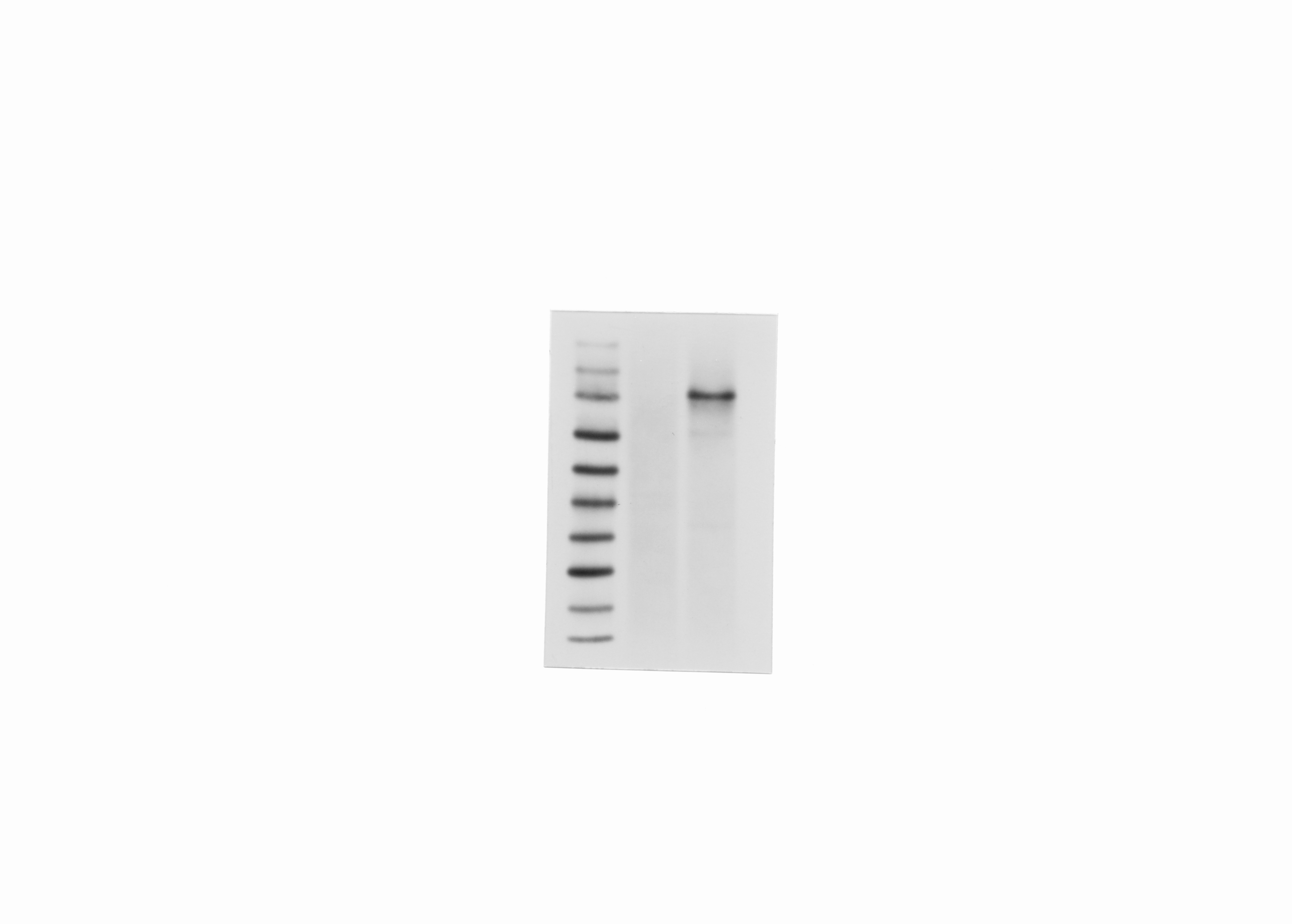

Supplement: Supplemental Information 1 [file peerj-13-20156-s001.zip › 4I-1/BACH1-2.tif]

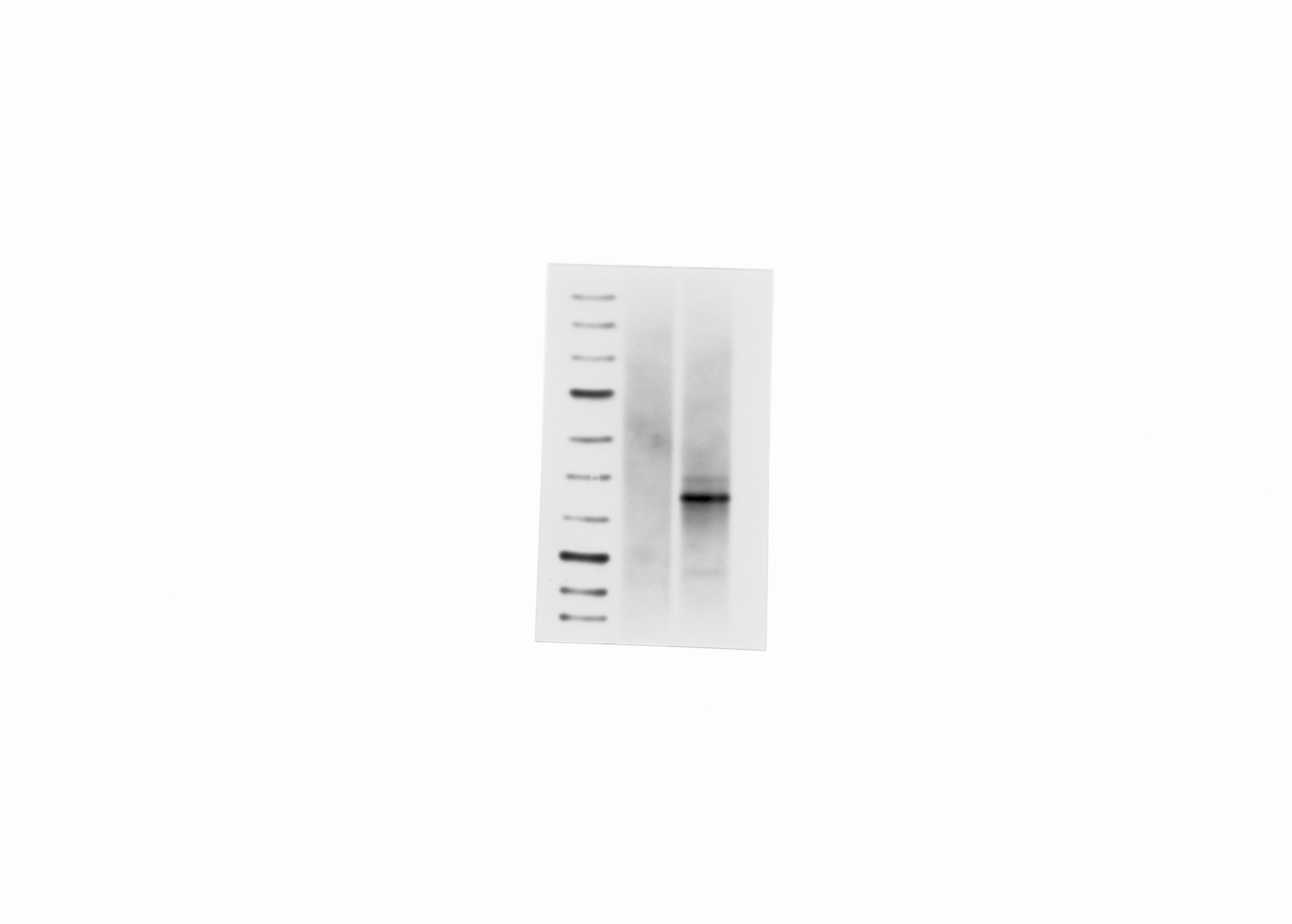

Supplement: Supplemental Information 1 [file peerj-13-20156-s001.zip › 4I-1/BAF53A-1.tif]

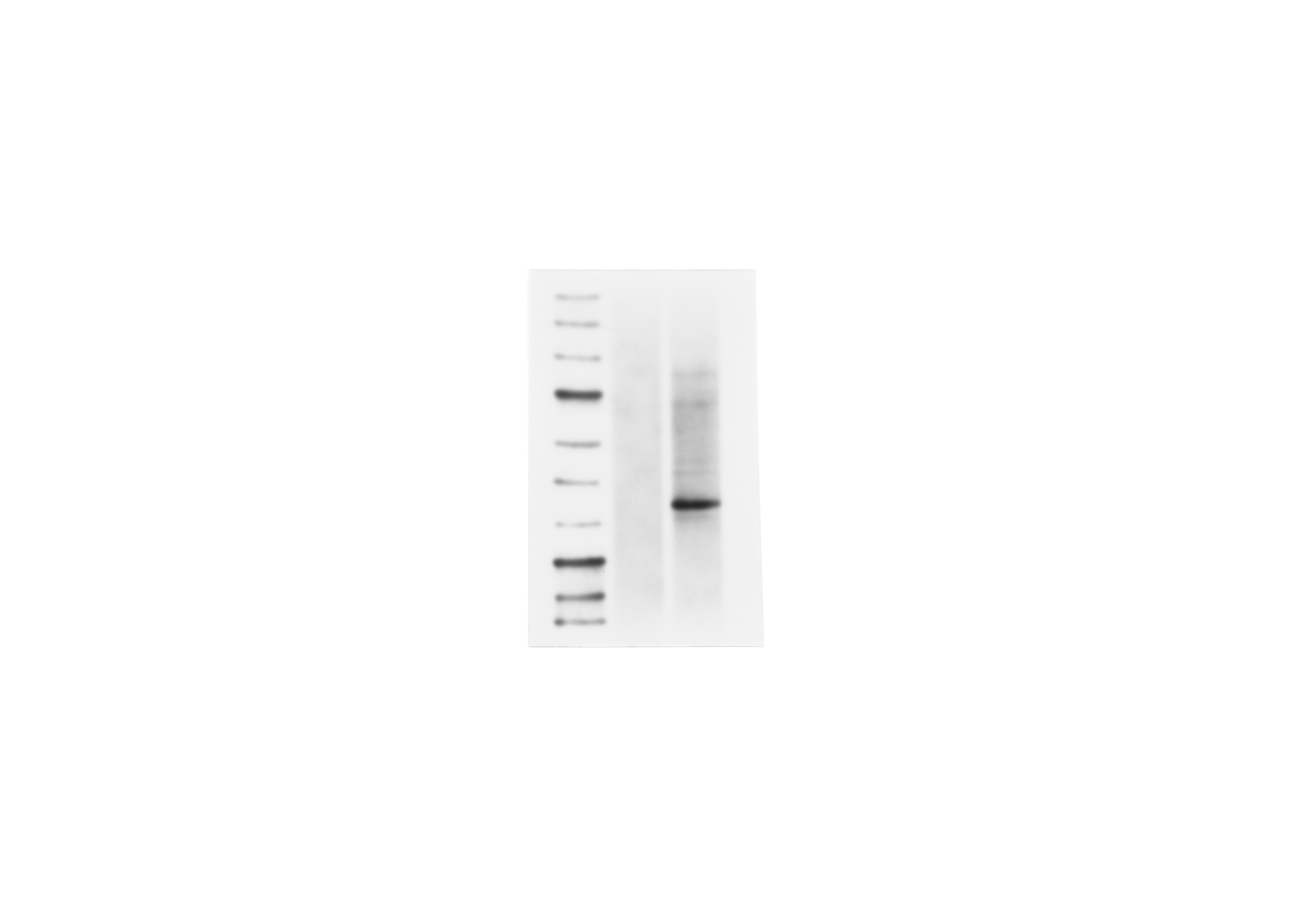

Supplement: Supplemental Information 1 [file peerj-13-20156-s001.zip › 4I-1/BAF53A-2.tif]

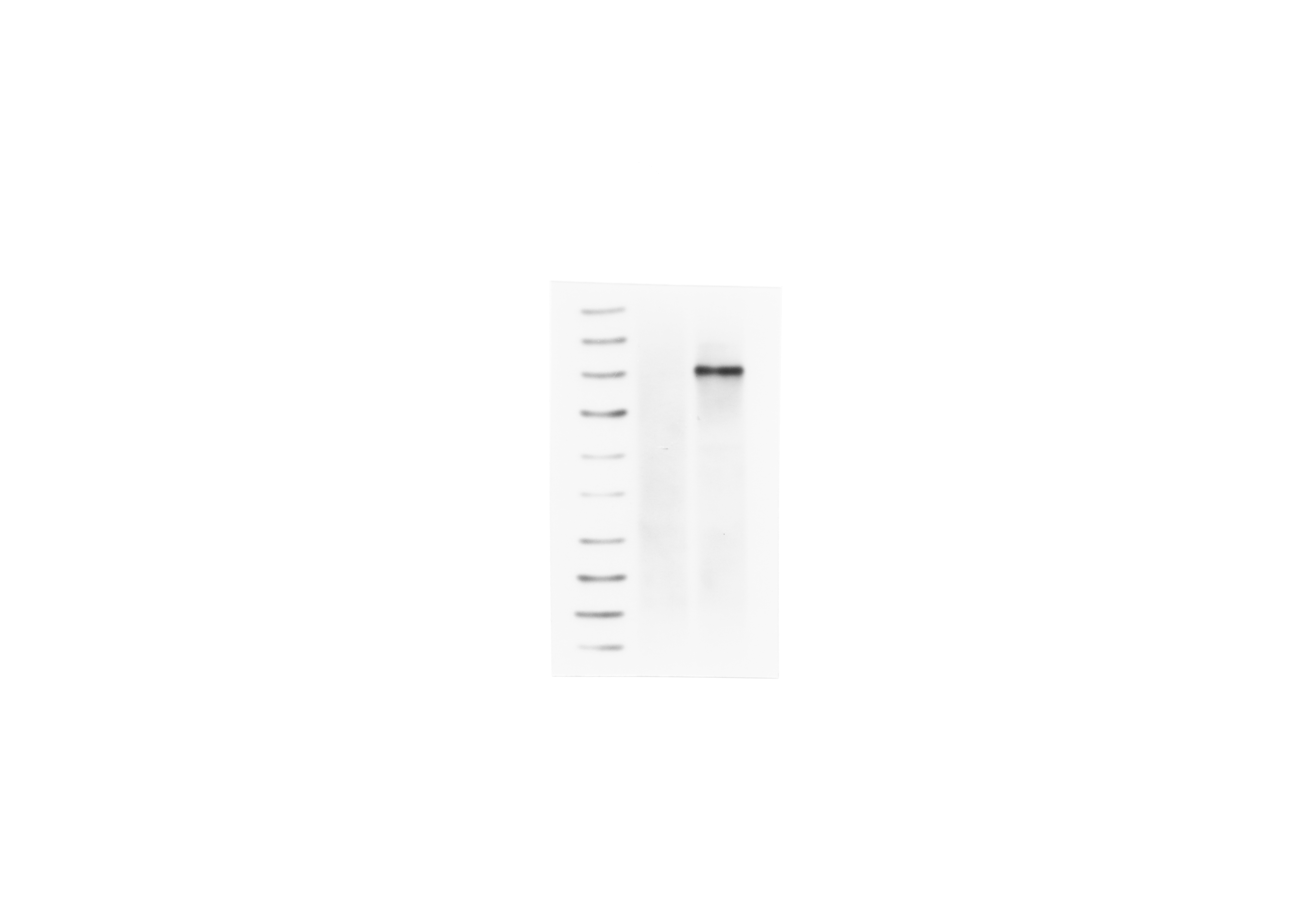

Supplement: Supplemental Information 1 [file peerj-13-20156-s001.zip › 4I-2/BACH1-1.tif]

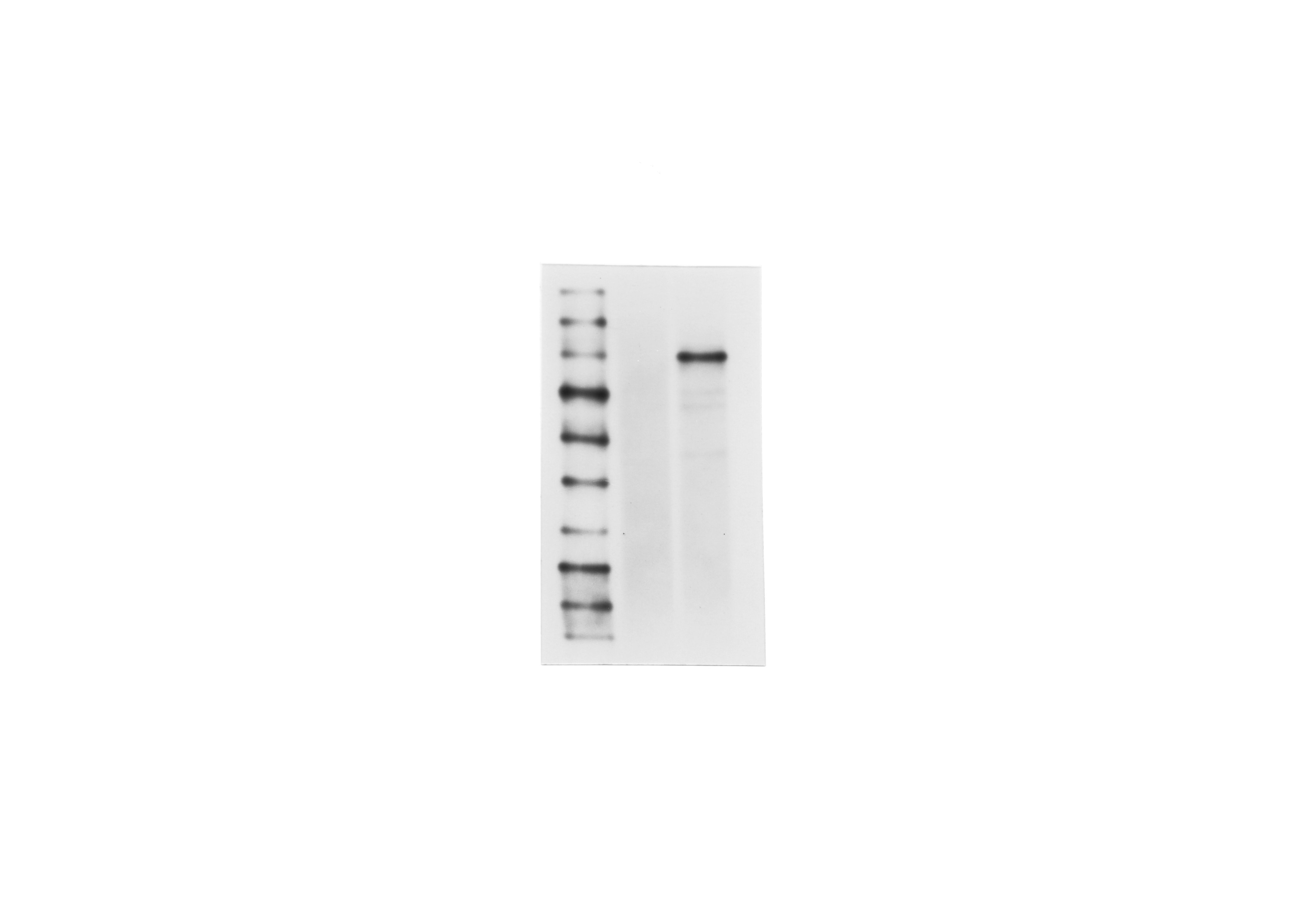

Supplement: Supplemental Information 1 [file peerj-13-20156-s001.zip › 4I-2/BACH1-2.tif]

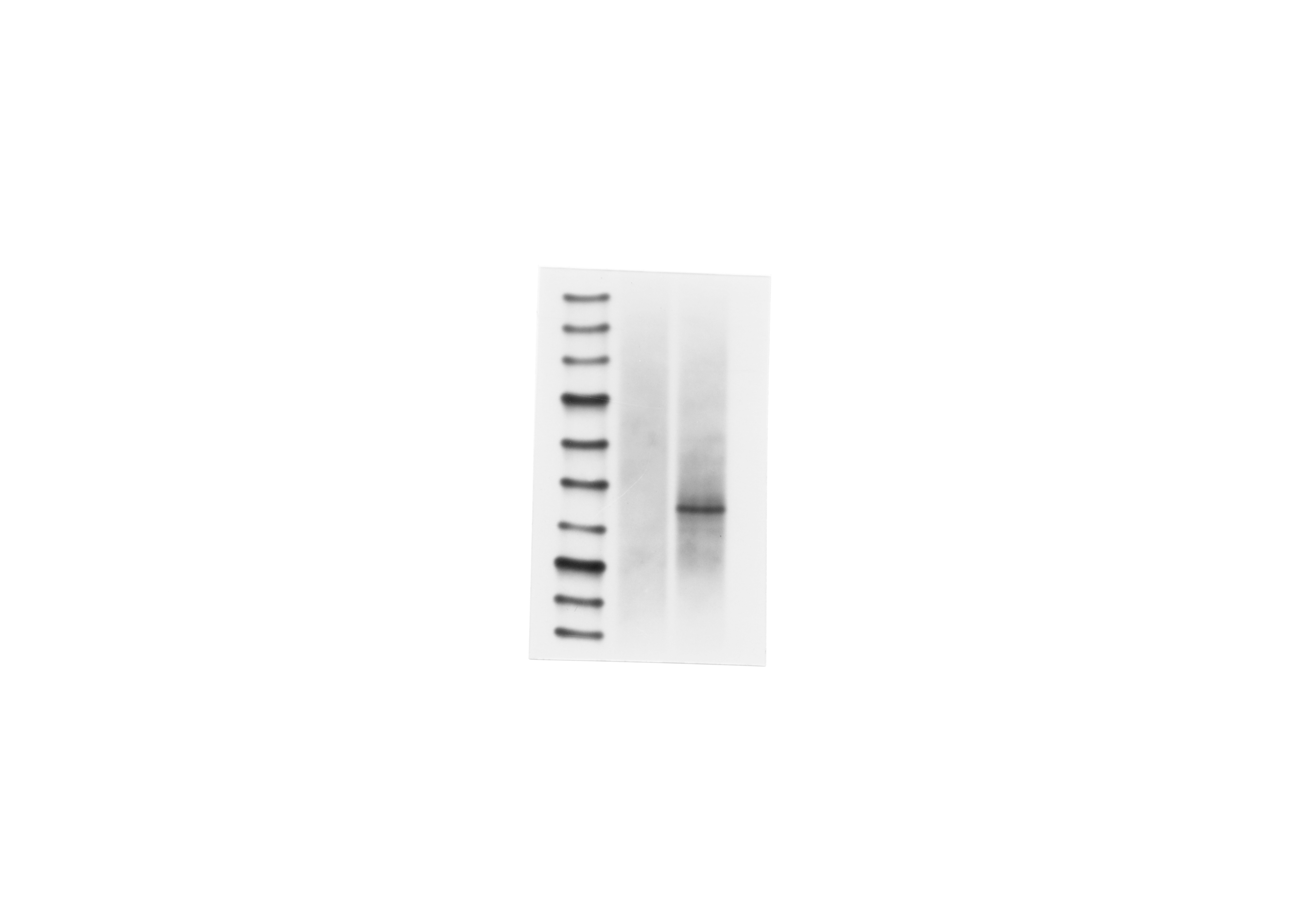

Supplement: Supplemental Information 1 [file peerj-13-20156-s001.zip › 4I-2/BAF53A-1.tif]

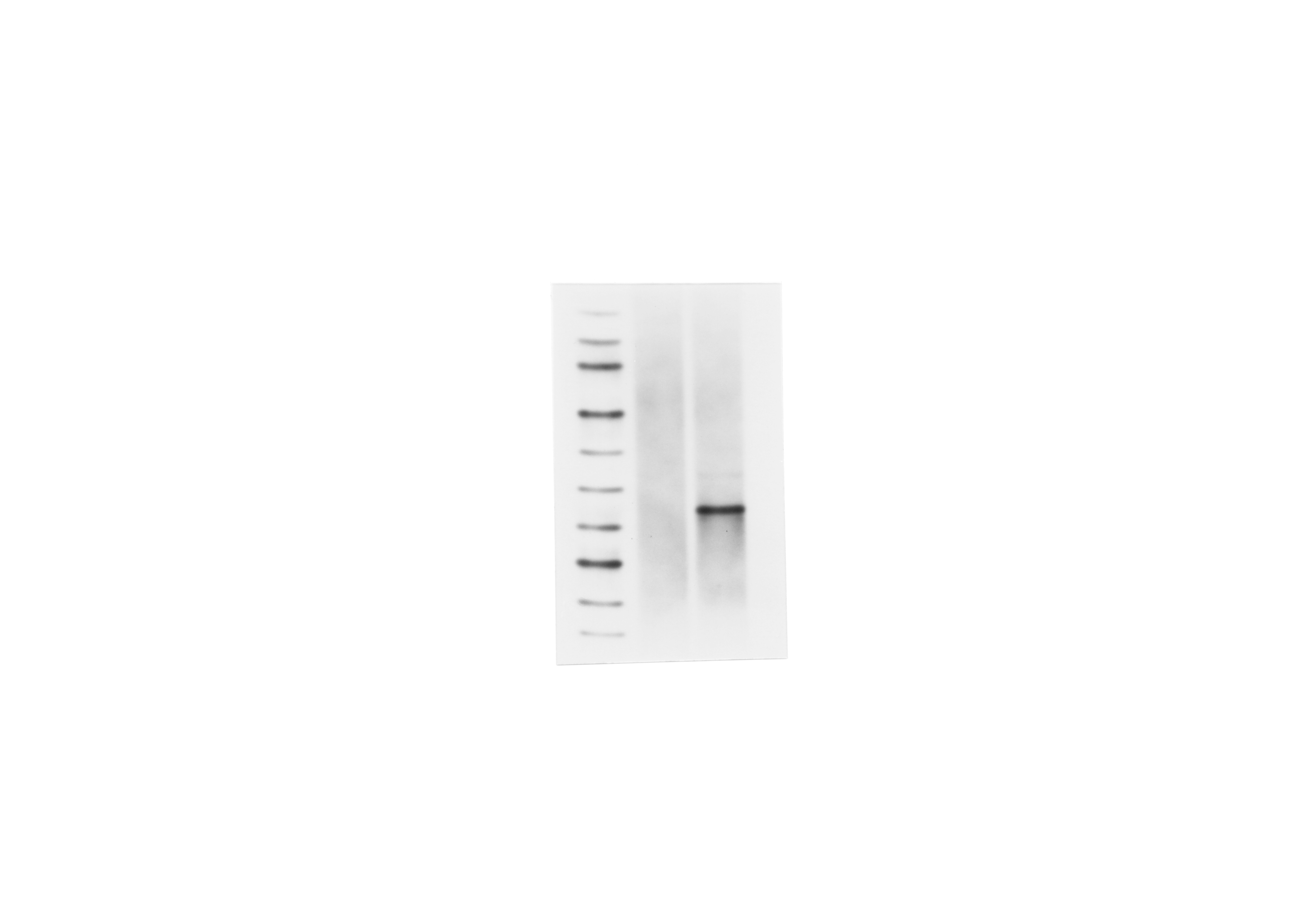

Supplement: Supplemental Information 1 [file peerj-13-20156-s001.zip › 4I-2/BAF53A-2.tif]

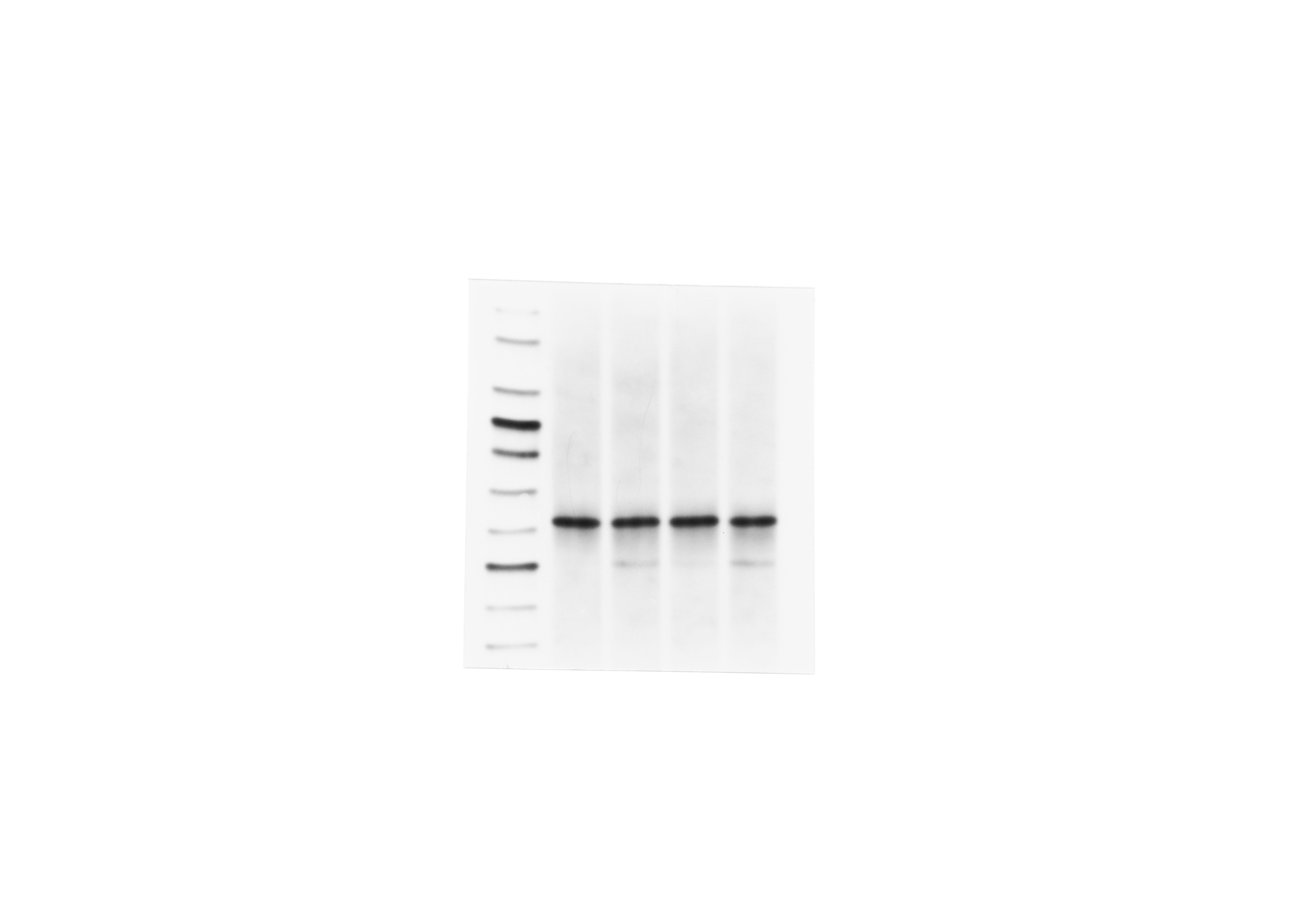

Supplement: Supplemental Information 1 [file peerj-13-20156-s001.zip › 6B-1/ACTB.tif]

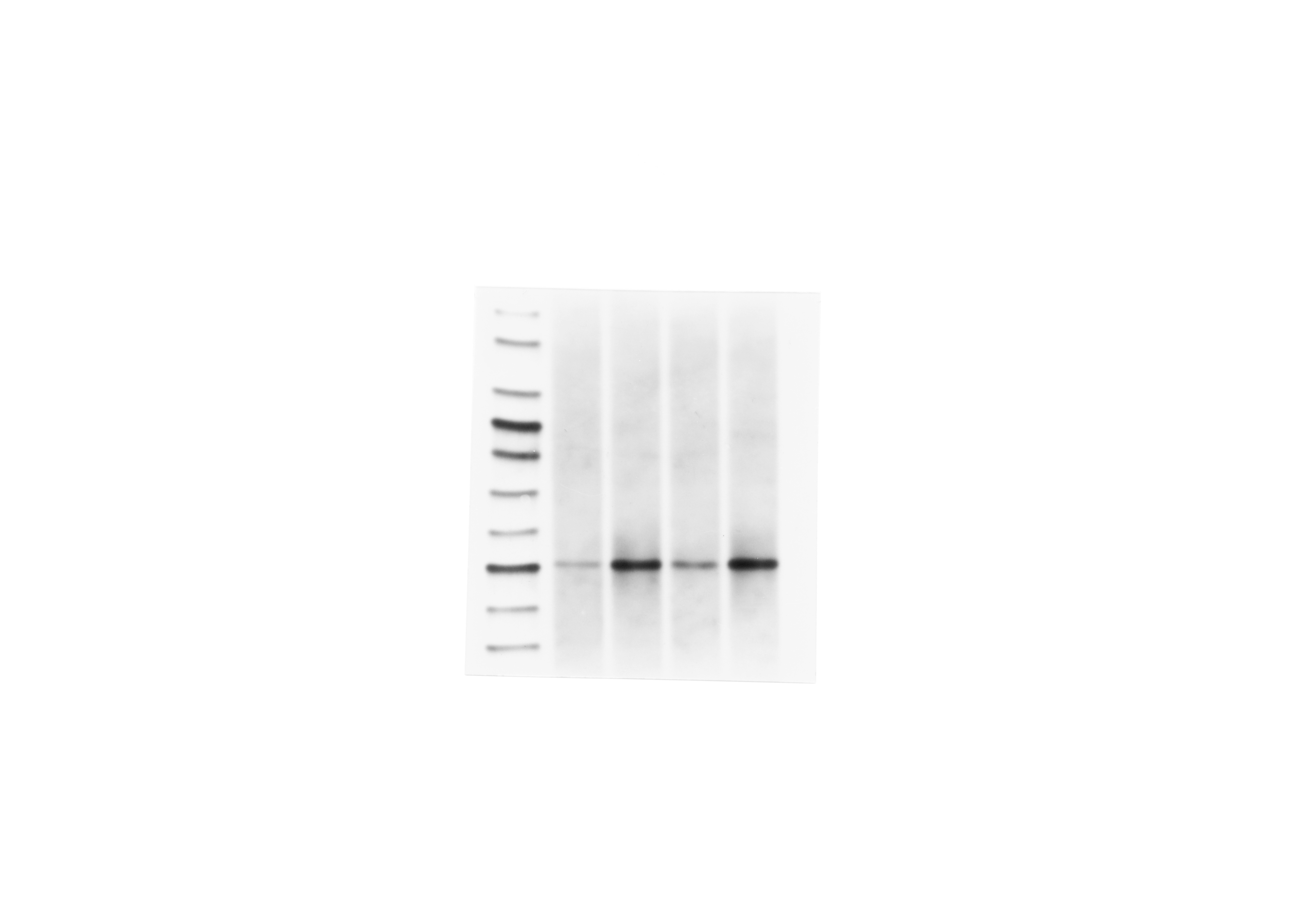

Supplement: Supplemental Information 1 [file peerj-13-20156-s001.zip › 6B-1/GCLM.tif]

1D

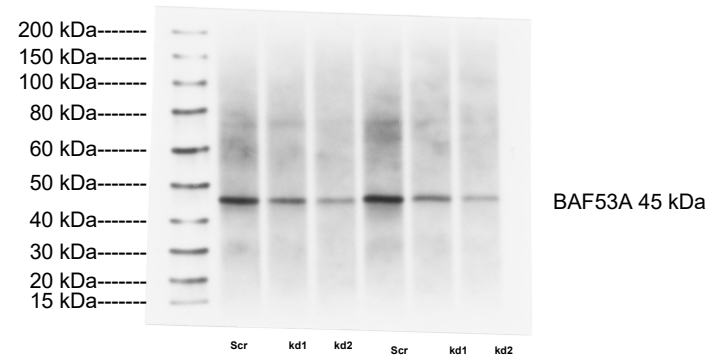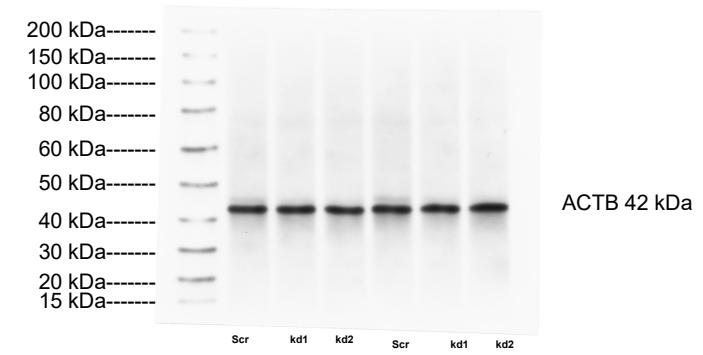

4B

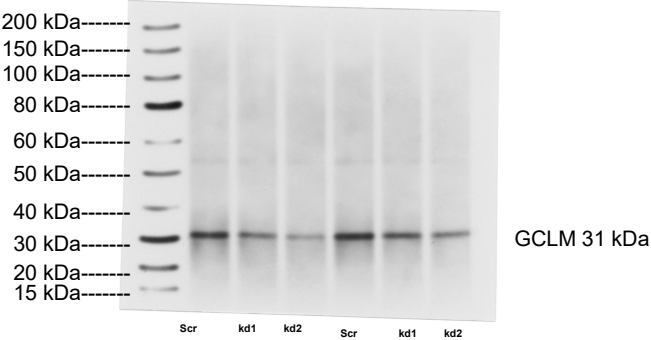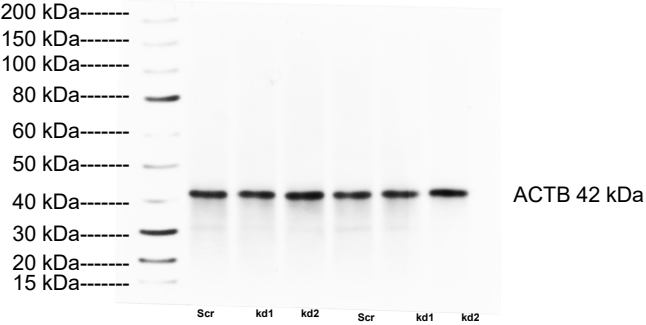

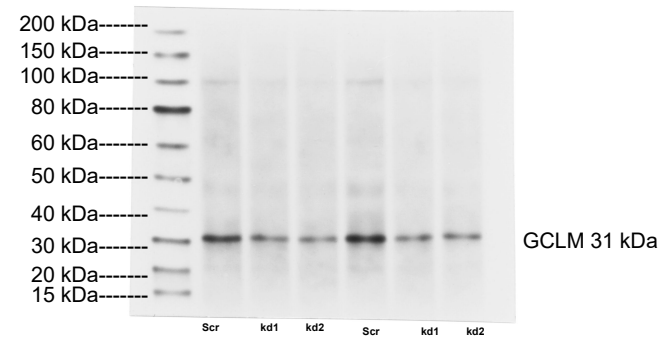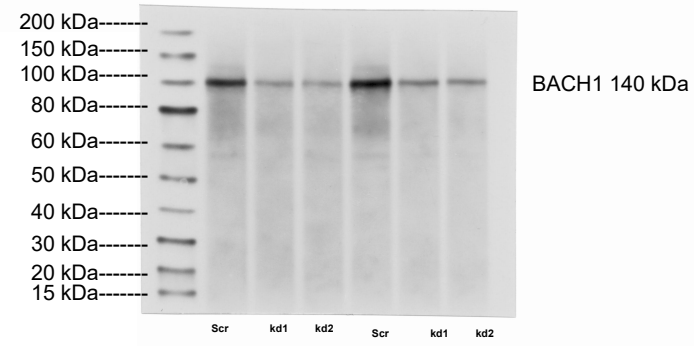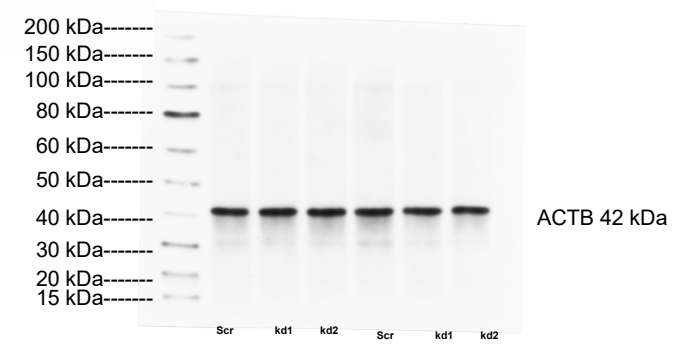

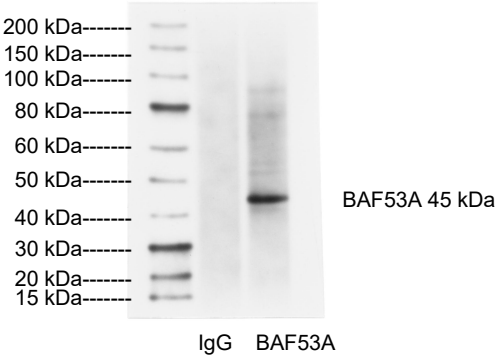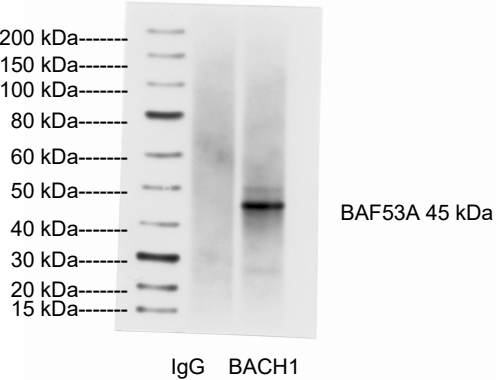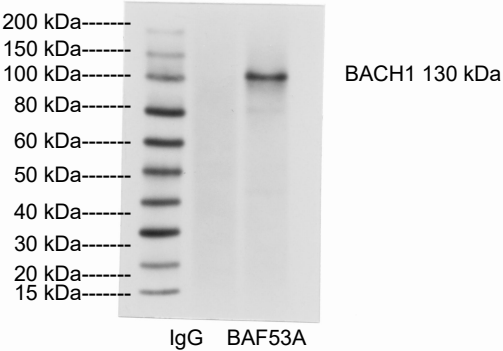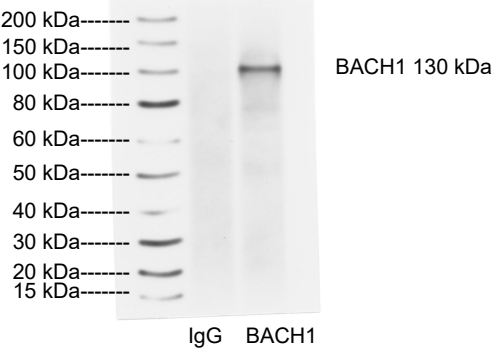

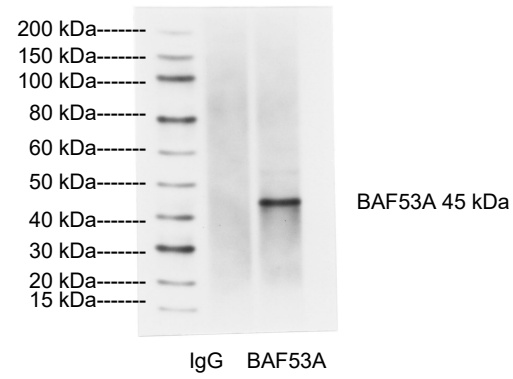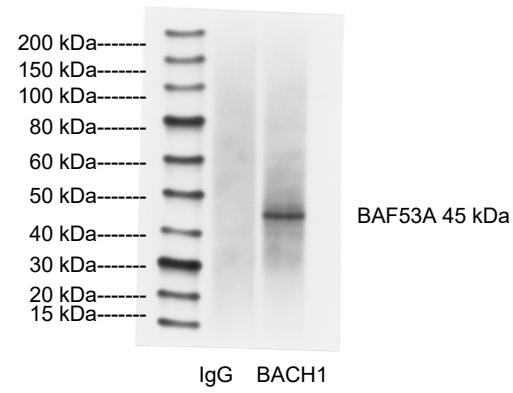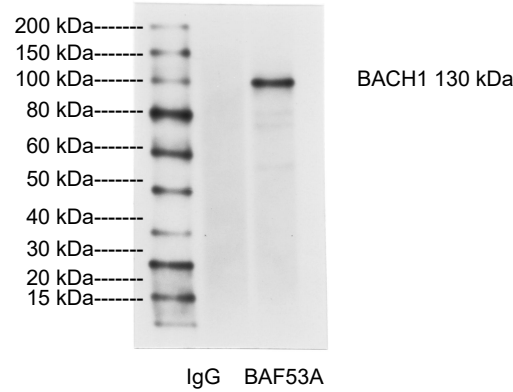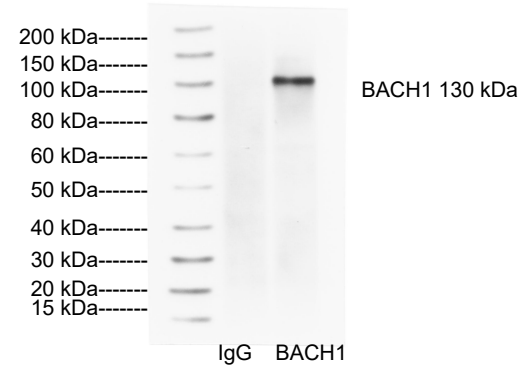

6B-1

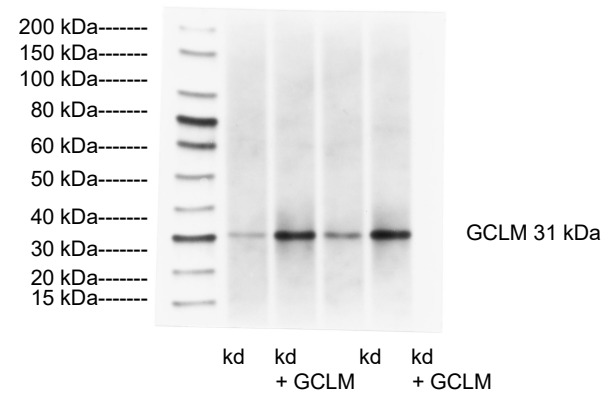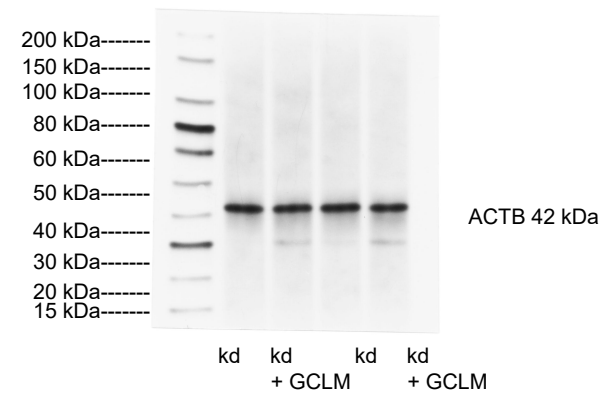

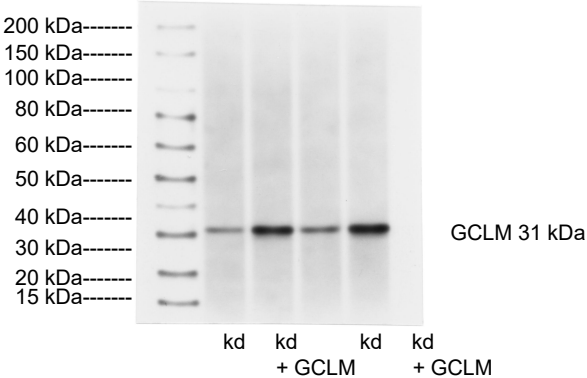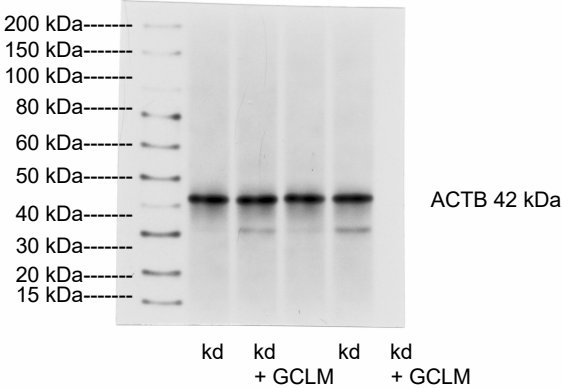

Supplement: Supplemental Information 3 [file peerj-13-20156-s003.pdf]

p-value = 0.012

R = 0.19

log<sub>2</sub>(GCLM TPM)

8

7

6

5

4

3

2

2

3

4

6

log<sub>2</sub>(BACH1 TPM)

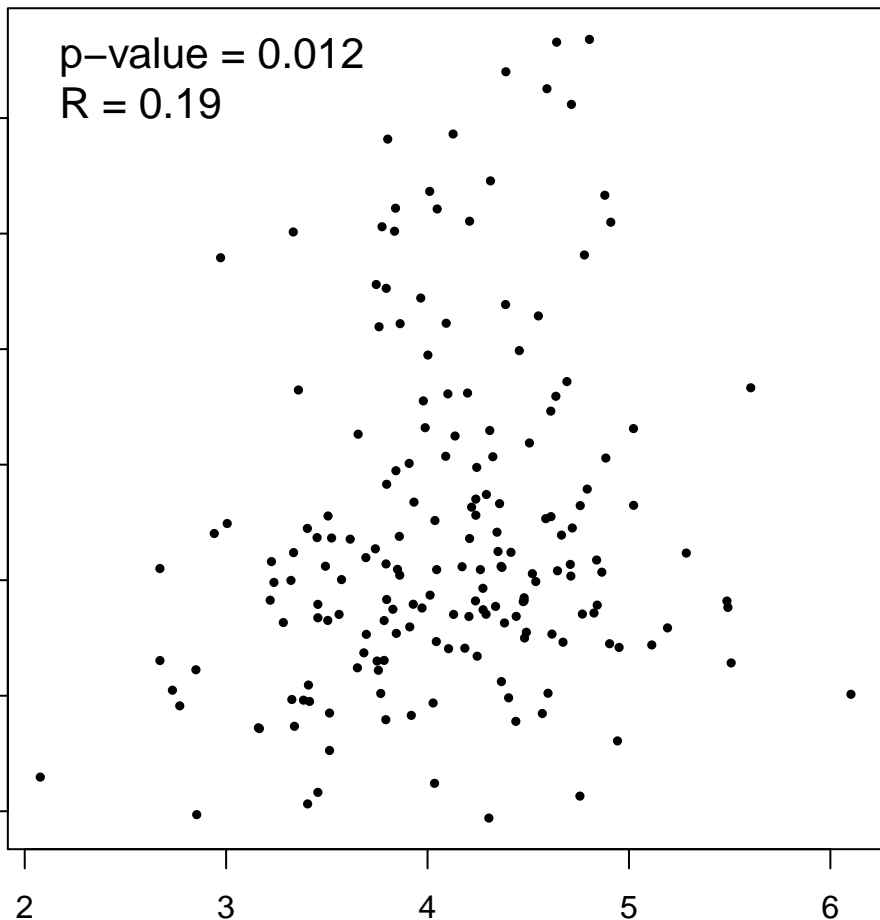

Supplement: Supplemental Information 4 [file peerj-13-20156-s004.zip › 6I2.pdf]

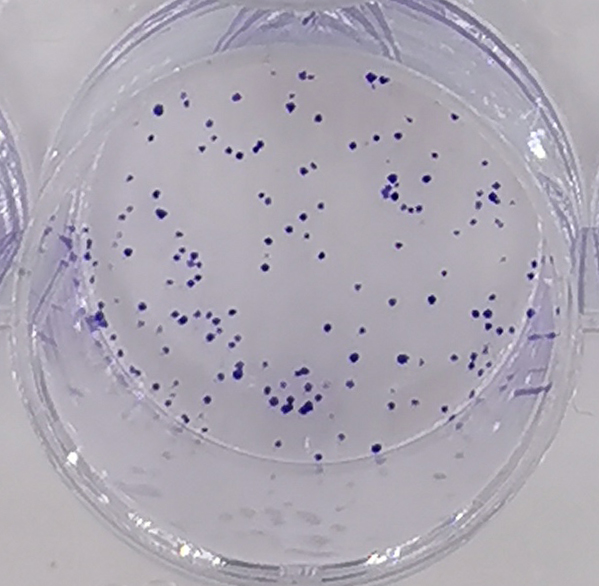

Supplement: Supplemental Information 4 [file peerj-13-20156-s004.zip › 1F/1.jpg]

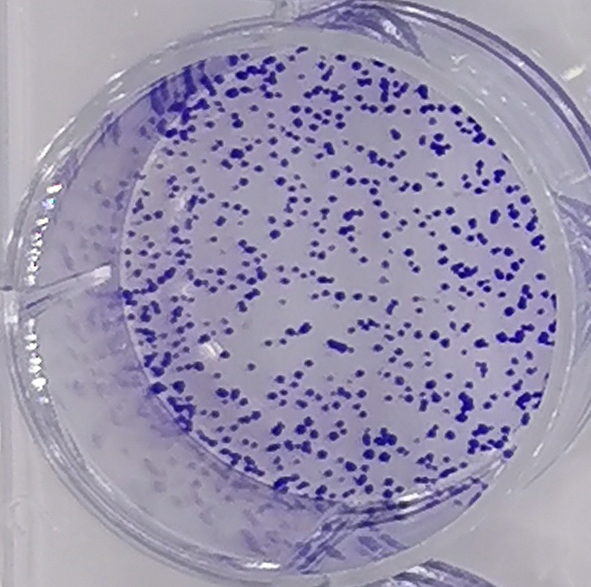

Supplement: Supplemental Information 4 [file peerj-13-20156-s004.zip › 1F/2.jpg]

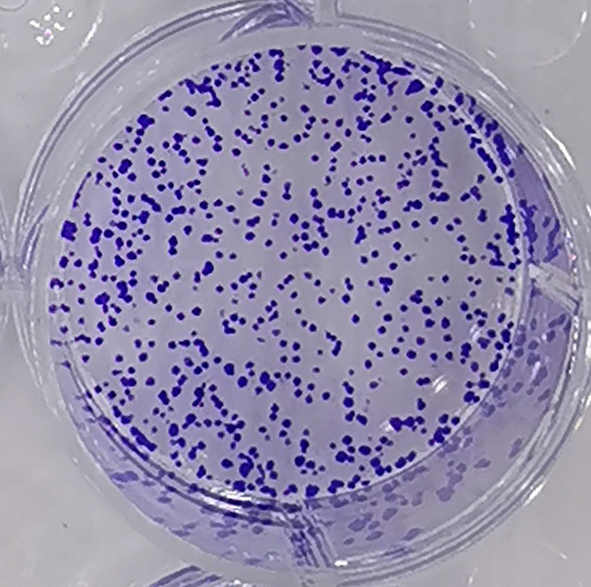

Supplement: Supplemental Information 4 [file peerj-13-20156-s004.zip › 1F/3.jpg]

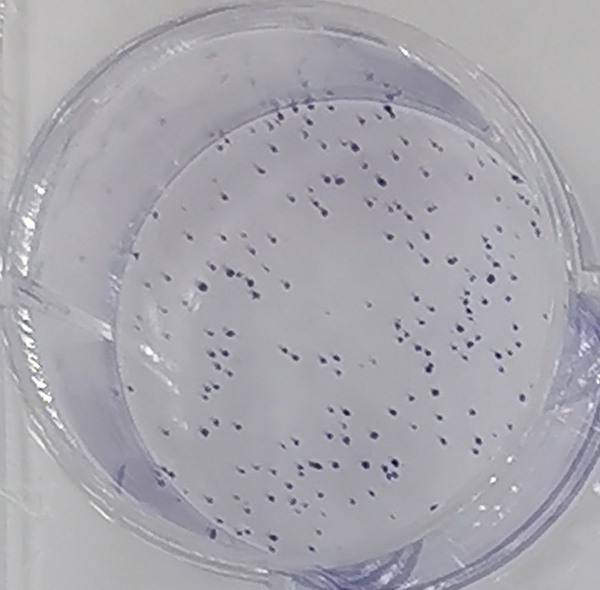

Supplement: Supplemental Information 4 [file peerj-13-20156-s004.zip › 1F/4.jpg]

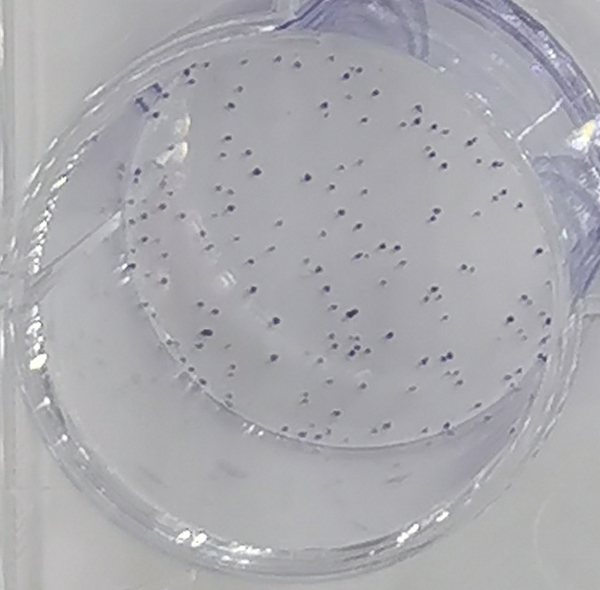

Supplement: Supplemental Information 4 [file peerj-13-20156-s004.zip › 1F/5.jpg]

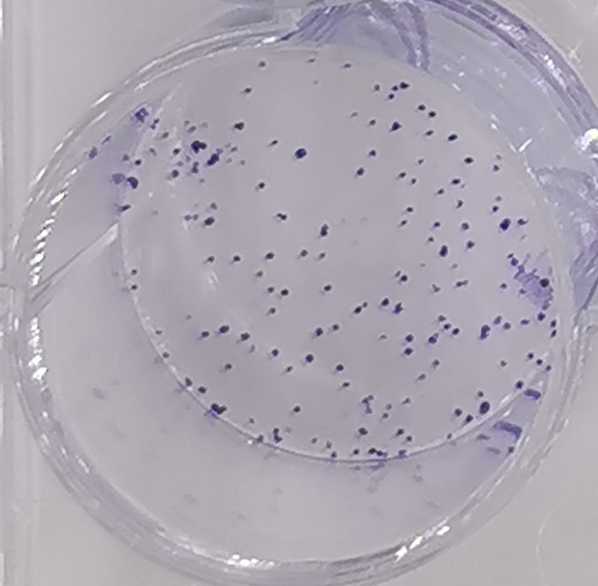

Supplement: Supplemental Information 4 [file peerj-13-20156-s004.zip › 1F/6.jpg]

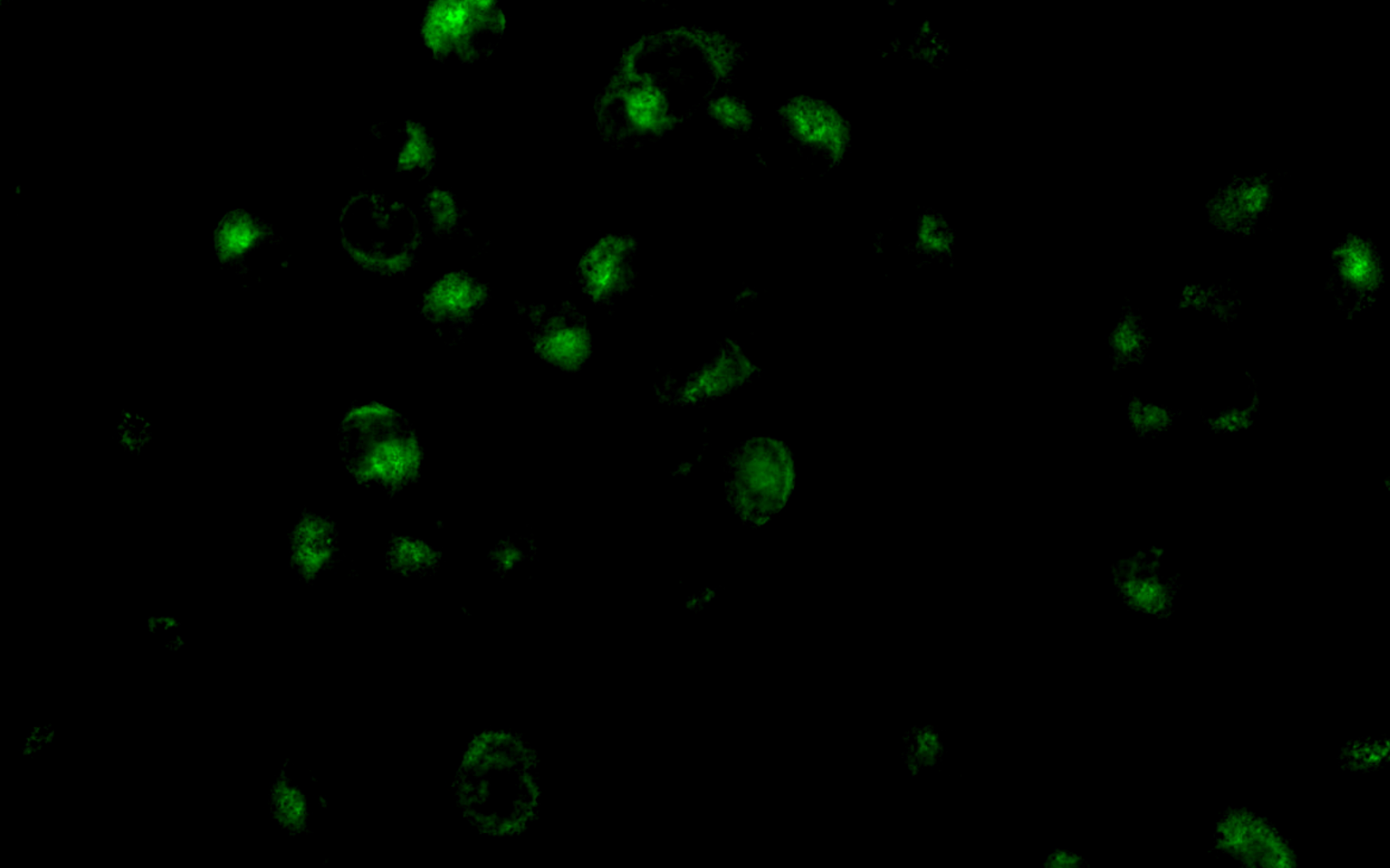

Supplement: Supplemental Information 4 [file peerj-13-20156-s004.zip › 2E/1.jpg]

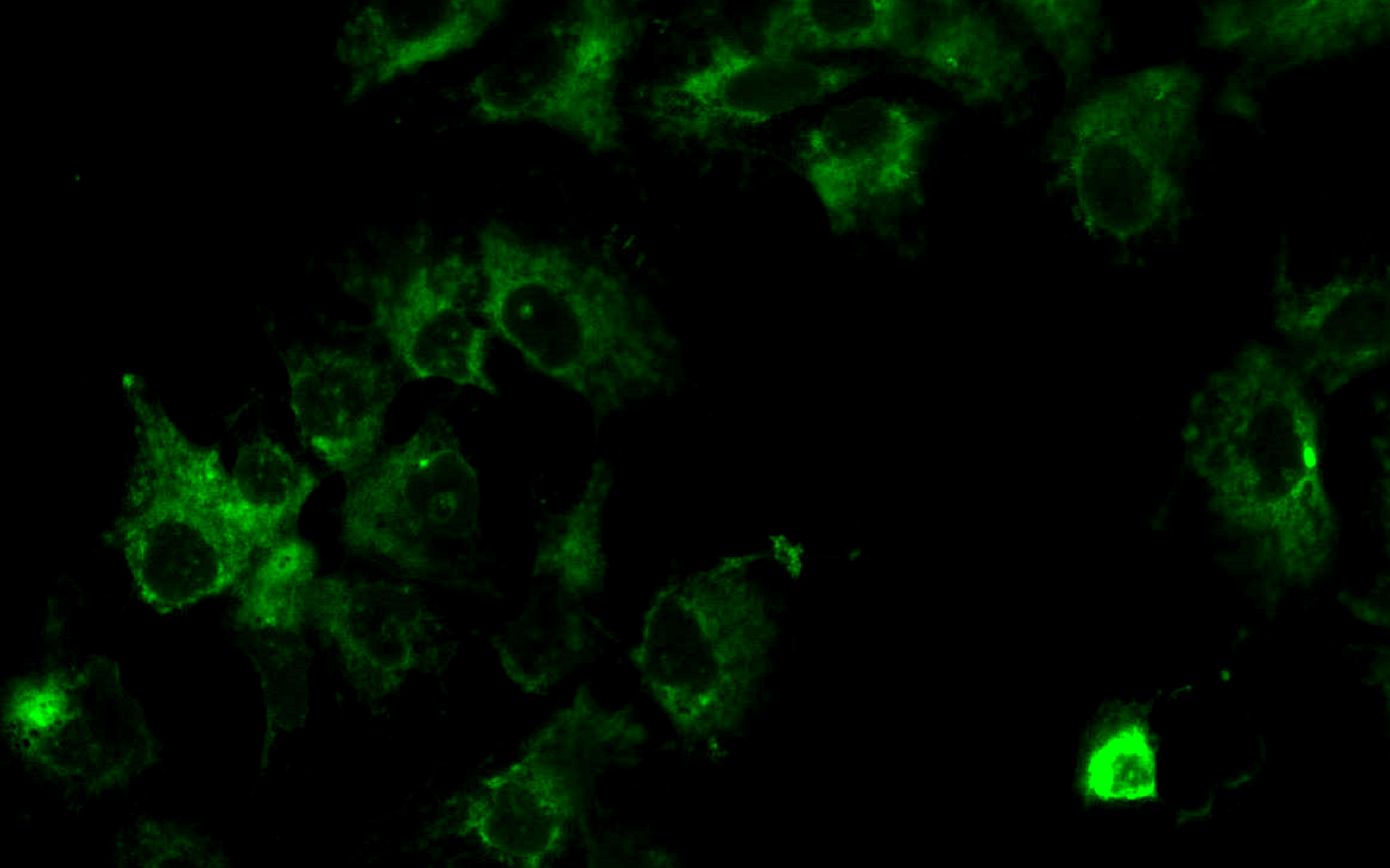

Supplement: Supplemental Information 4 [file peerj-13-20156-s004.zip › 2E/10.jpg]

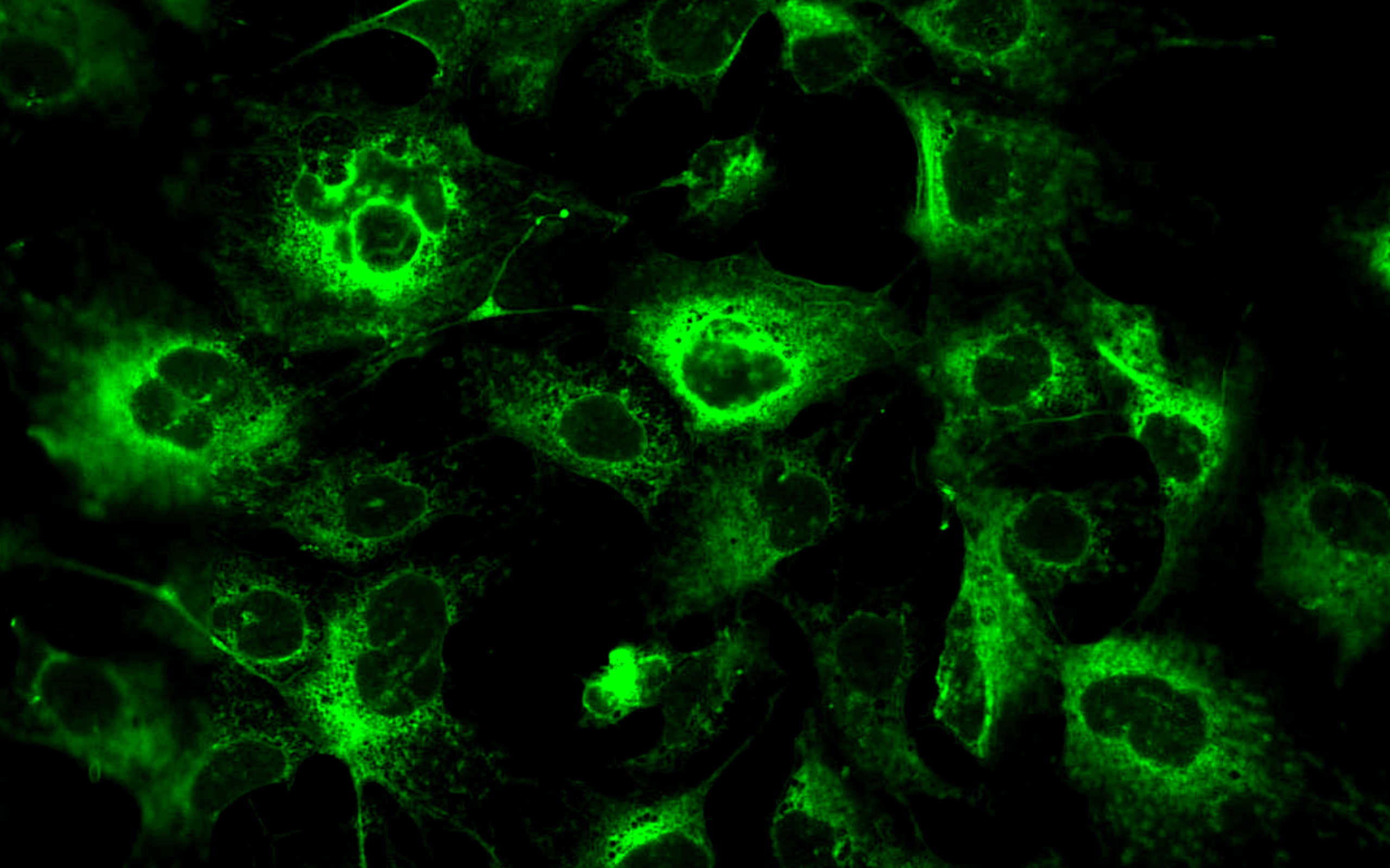

Supplement: Supplemental Information 4 [file peerj-13-20156-s004.zip › 2E/11.jpg]

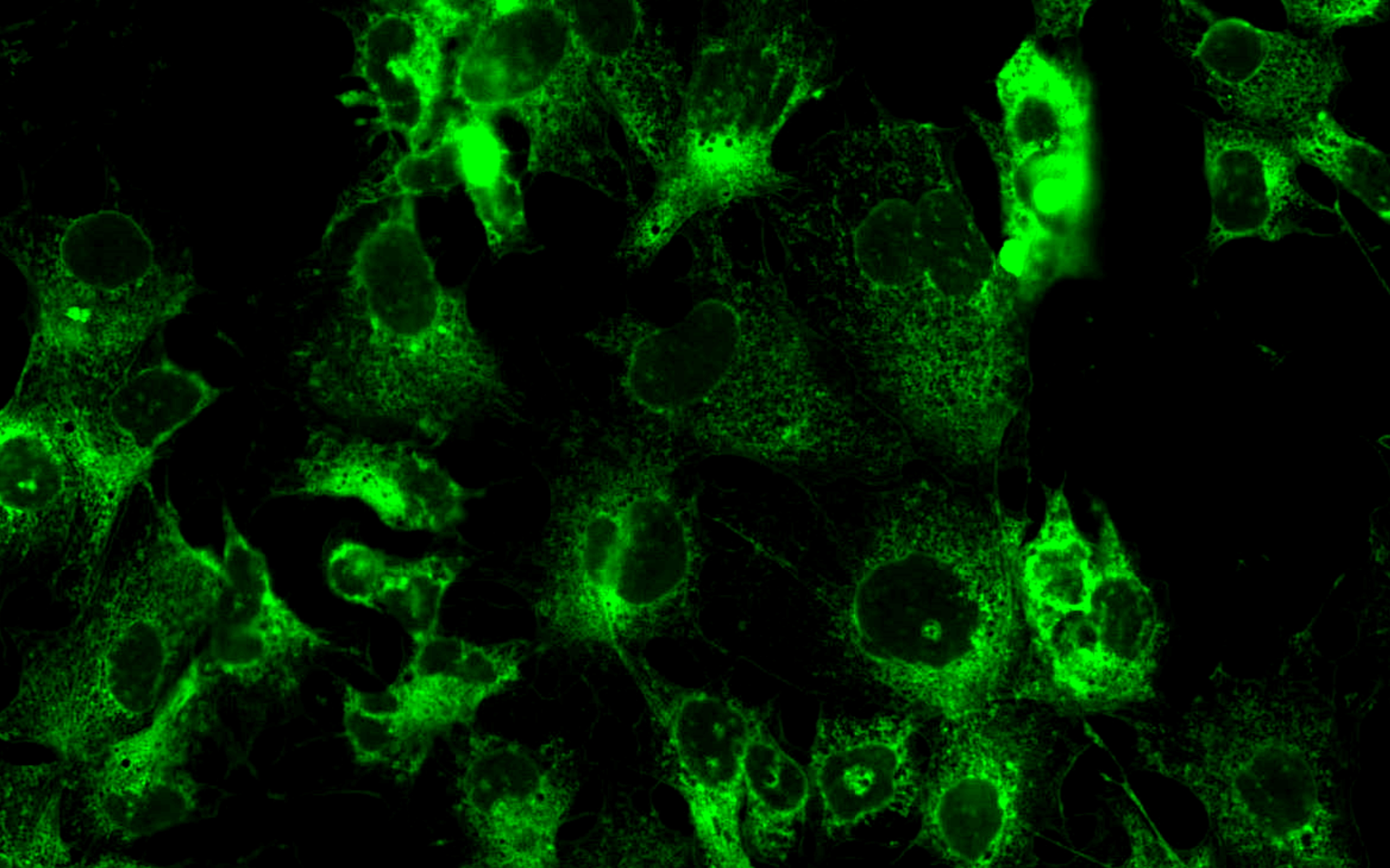

Supplement: Supplemental Information 4 [file peerj-13-20156-s004.zip › 2E/12.jpg]

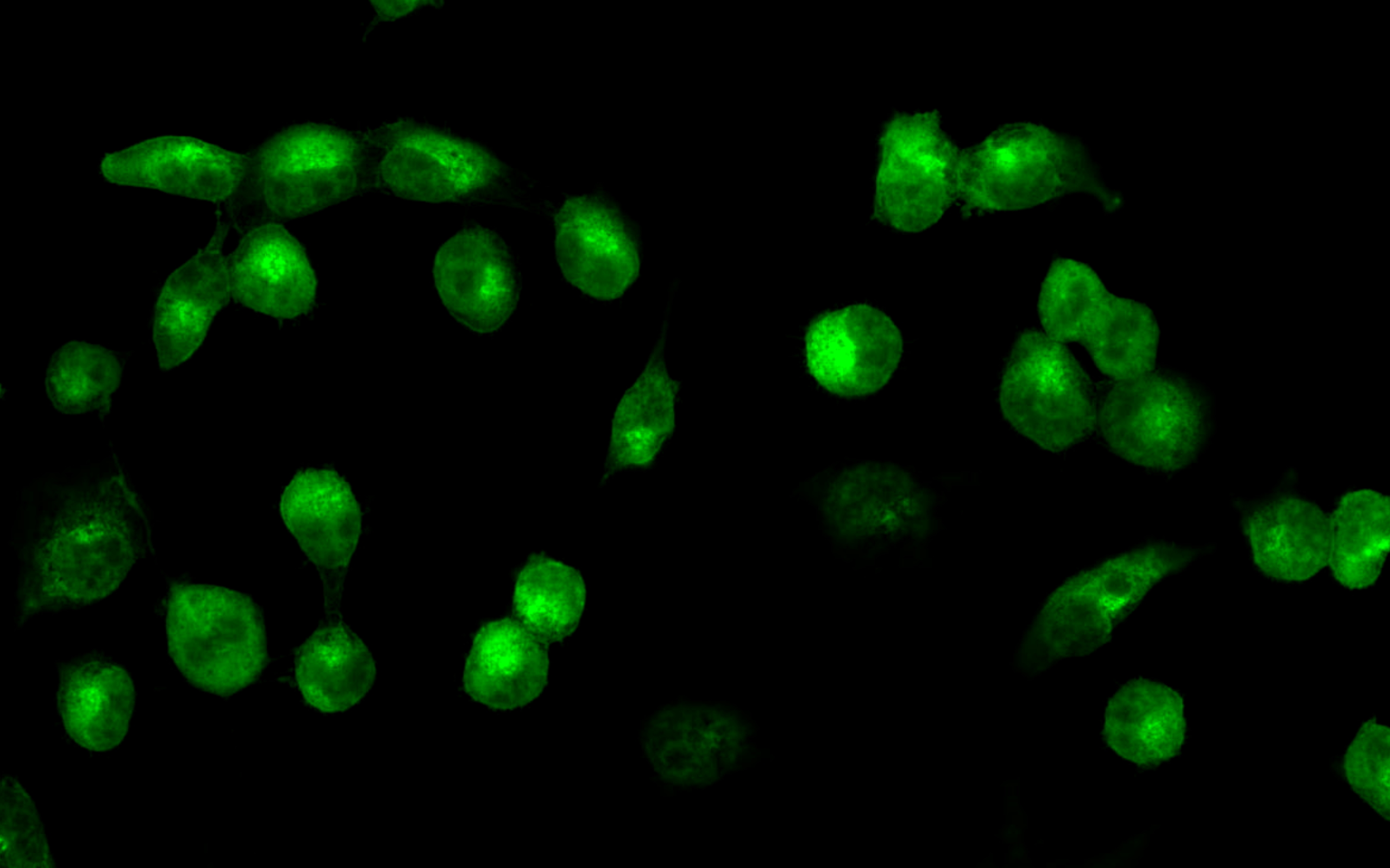

Supplement: Supplemental Information 4 [file peerj-13-20156-s004.zip › 2E/2.jpg]

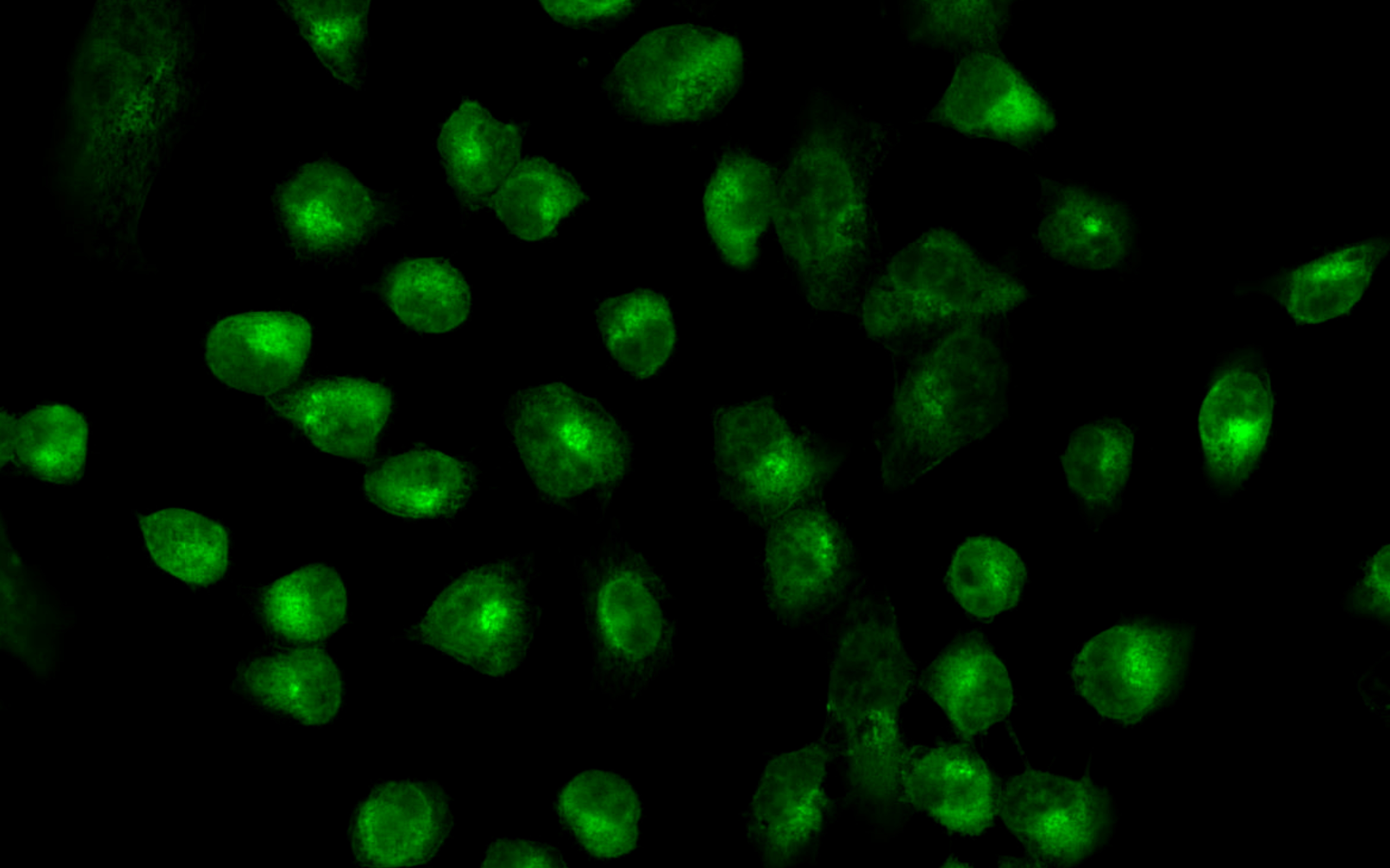

Supplement: Supplemental Information 4 [file peerj-13-20156-s004.zip › 2E/3.jpg]

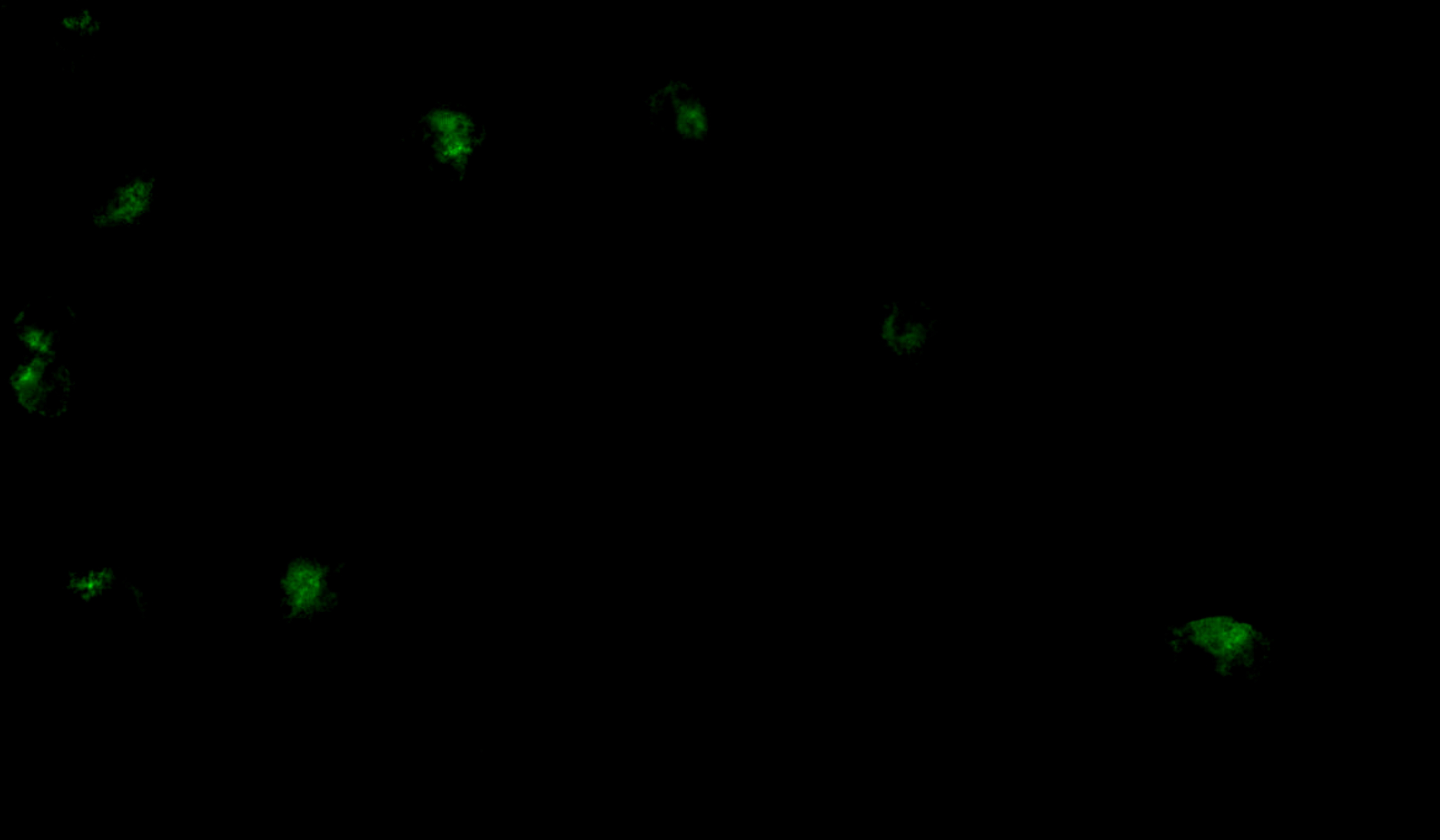

Supplement: Supplemental Information 4 [file peerj-13-20156-s004.zip › 2E/4.jpg]

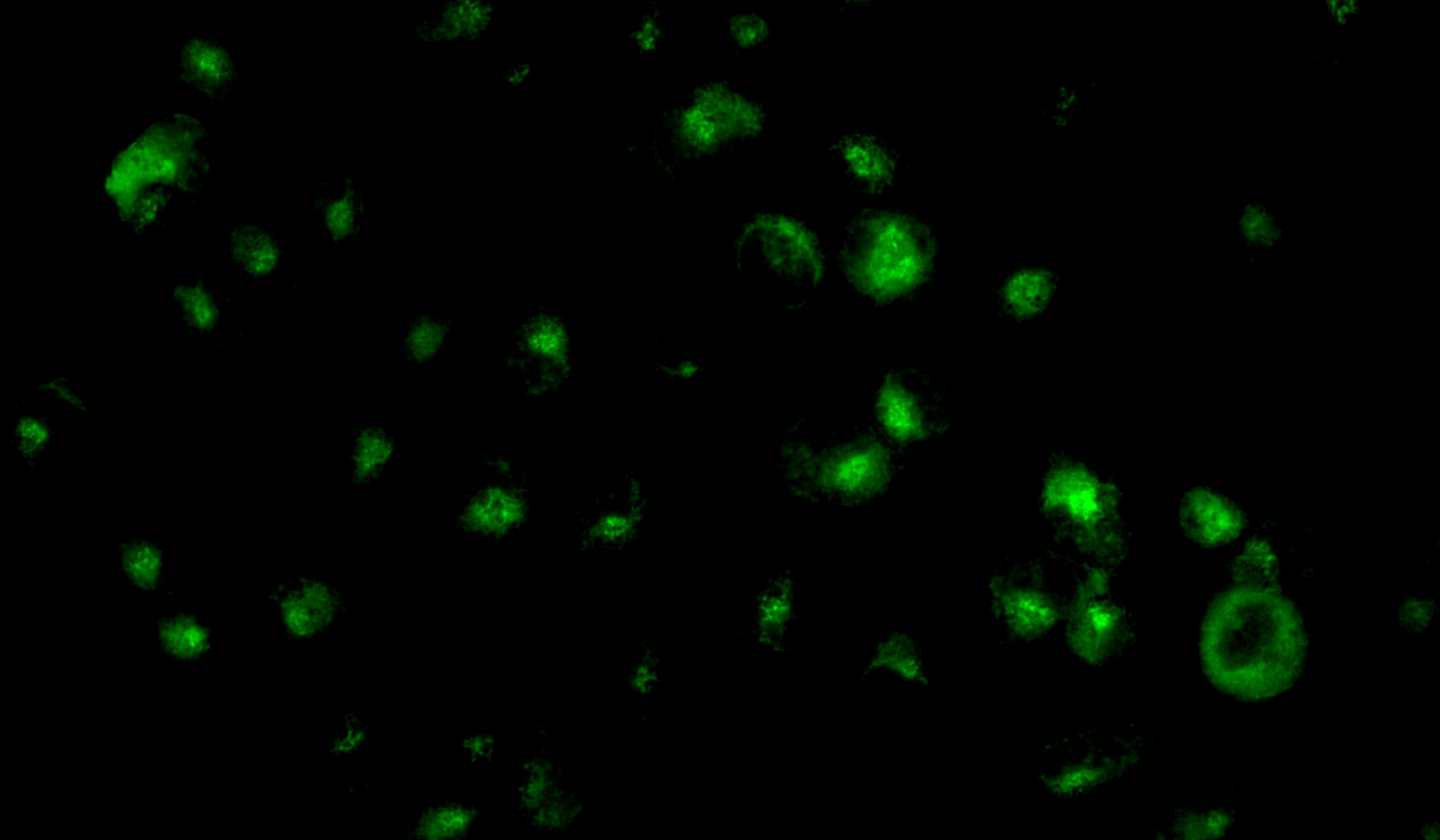

Supplement: Supplemental Information 4 [file peerj-13-20156-s004.zip › 2E/5.jpg]

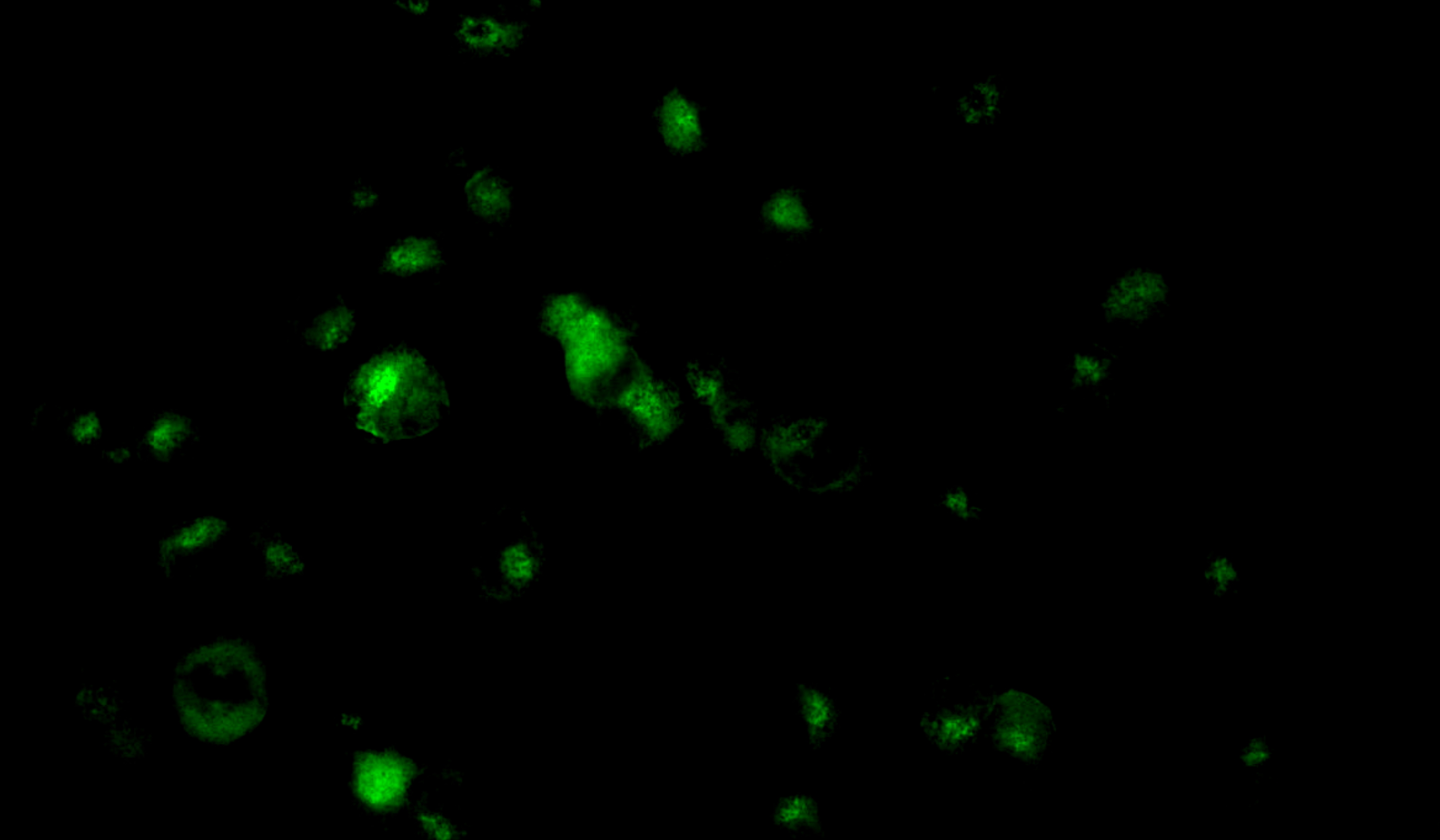

Supplement: Supplemental Information 4 [file peerj-13-20156-s004.zip › 2E/6.jpg]

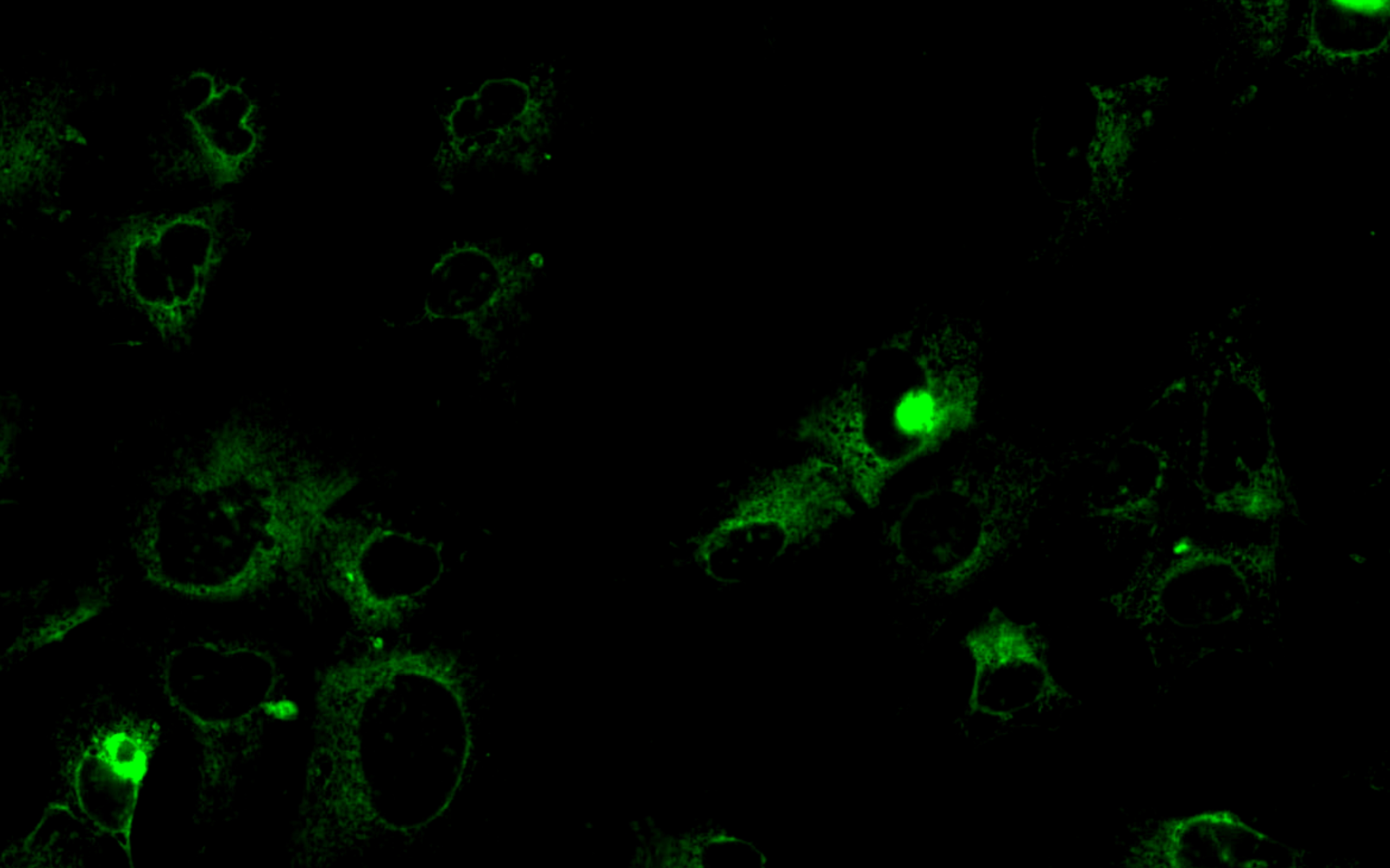

Supplement: Supplemental Information 4 [file peerj-13-20156-s004.zip › 2E/7.jpg]

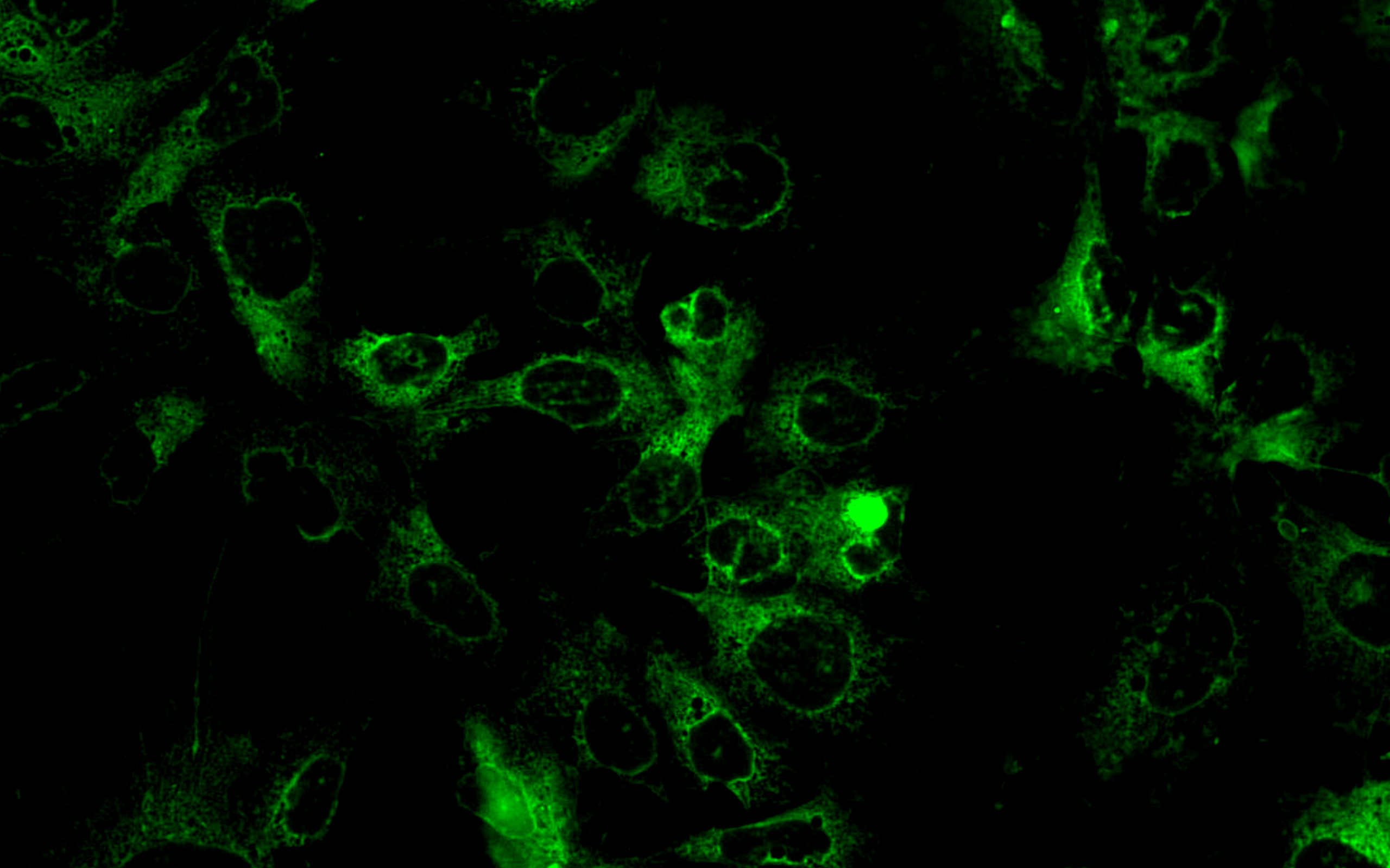

Supplement: Supplemental Information 4 [file peerj-13-20156-s004.zip › 2E/8.jpg]

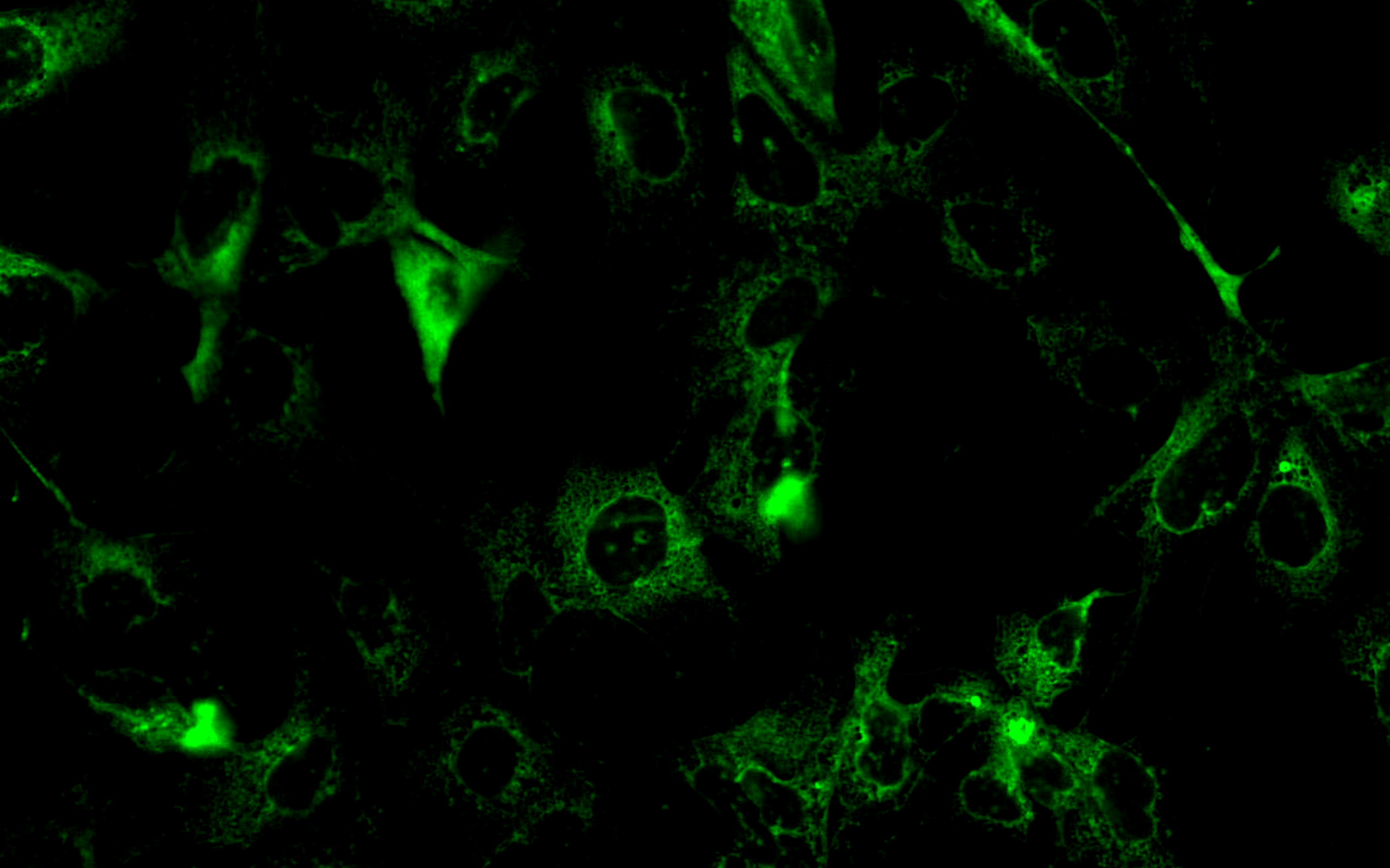

Supplement: Supplemental Information 4 [file peerj-13-20156-s004.zip › 2E/9.jpg]

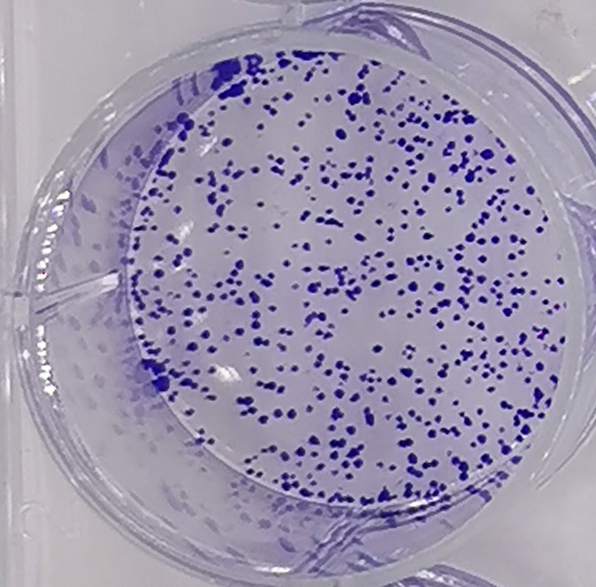

Supplement: Supplemental Information 4 [file peerj-13-20156-s004.zip › 2G/1.jpg]

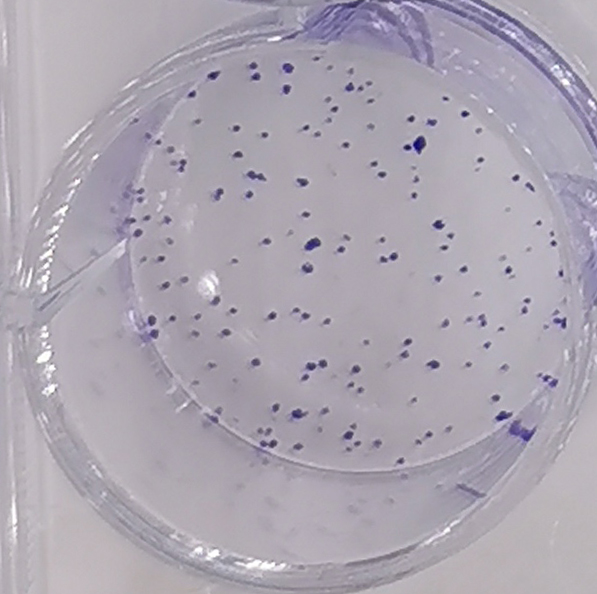

Supplement: Supplemental Information 4 [file peerj-13-20156-s004.zip › 2G/2.jpg]

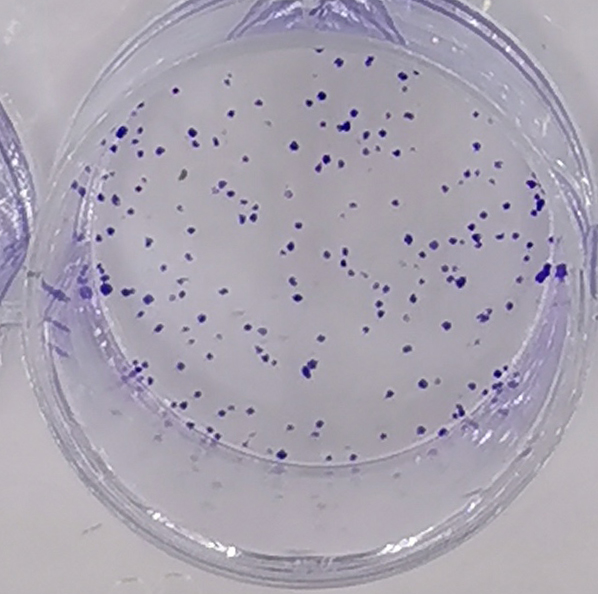

Supplement: Supplemental Information 4 [file peerj-13-20156-s004.zip › 2G/3.jpg]

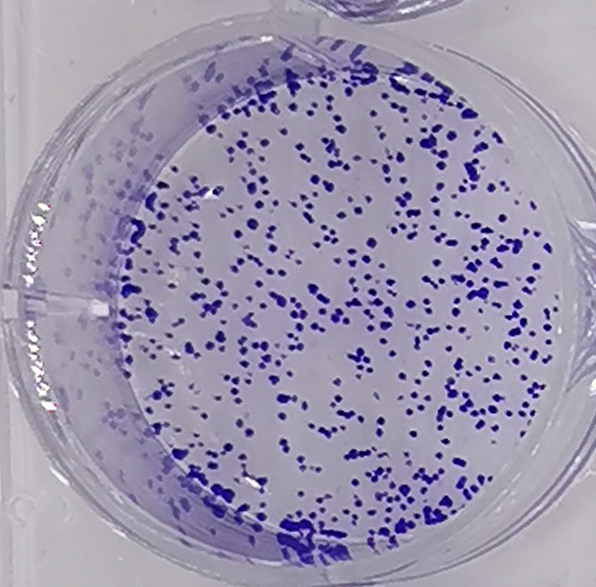

Supplement: Supplemental Information 4 [file peerj-13-20156-s004.zip › 2G/4.jpg]

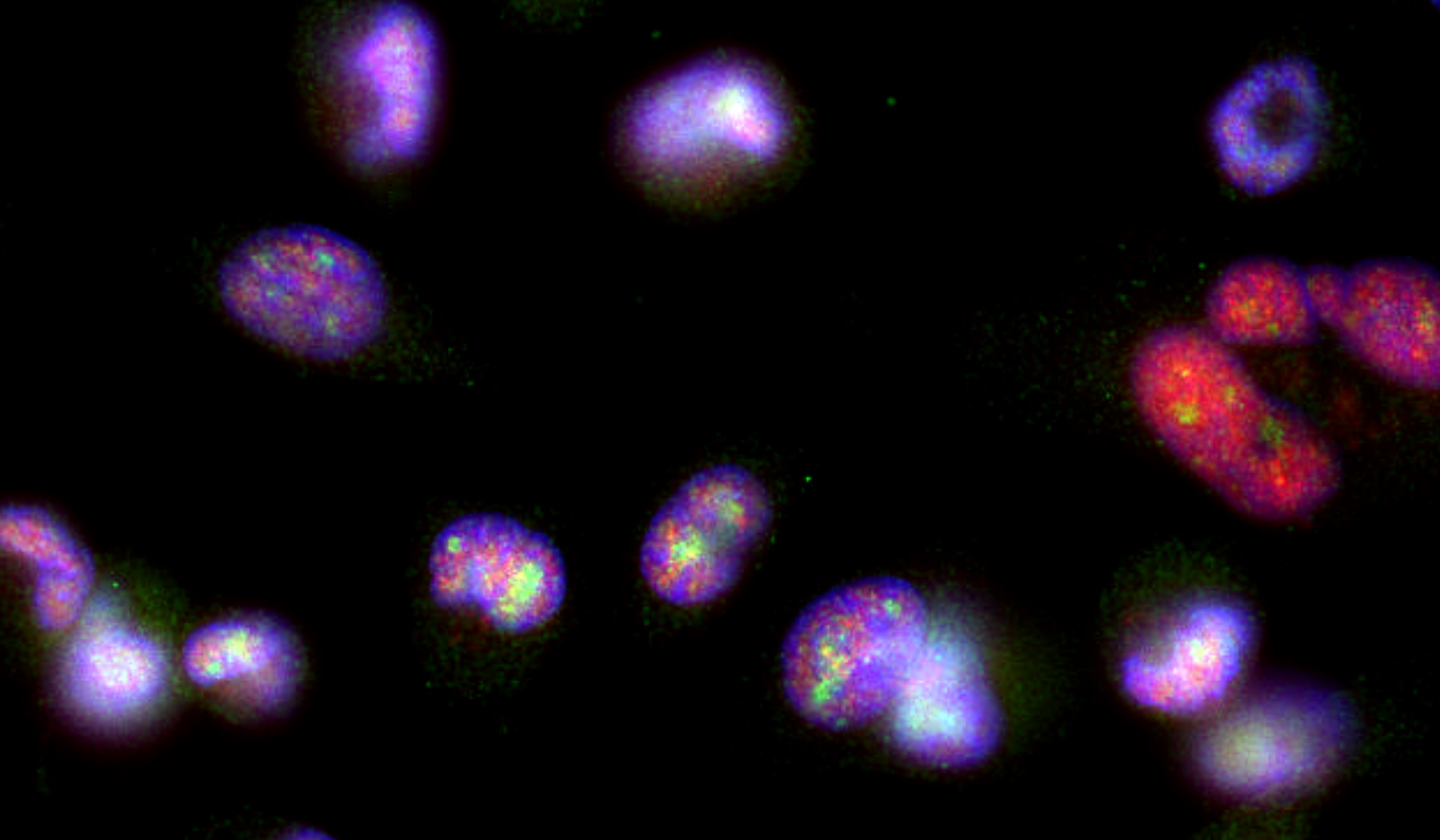

Supplement: Supplemental Information 4 [file peerj-13-20156-s004.zip › 4J/1.jpg]

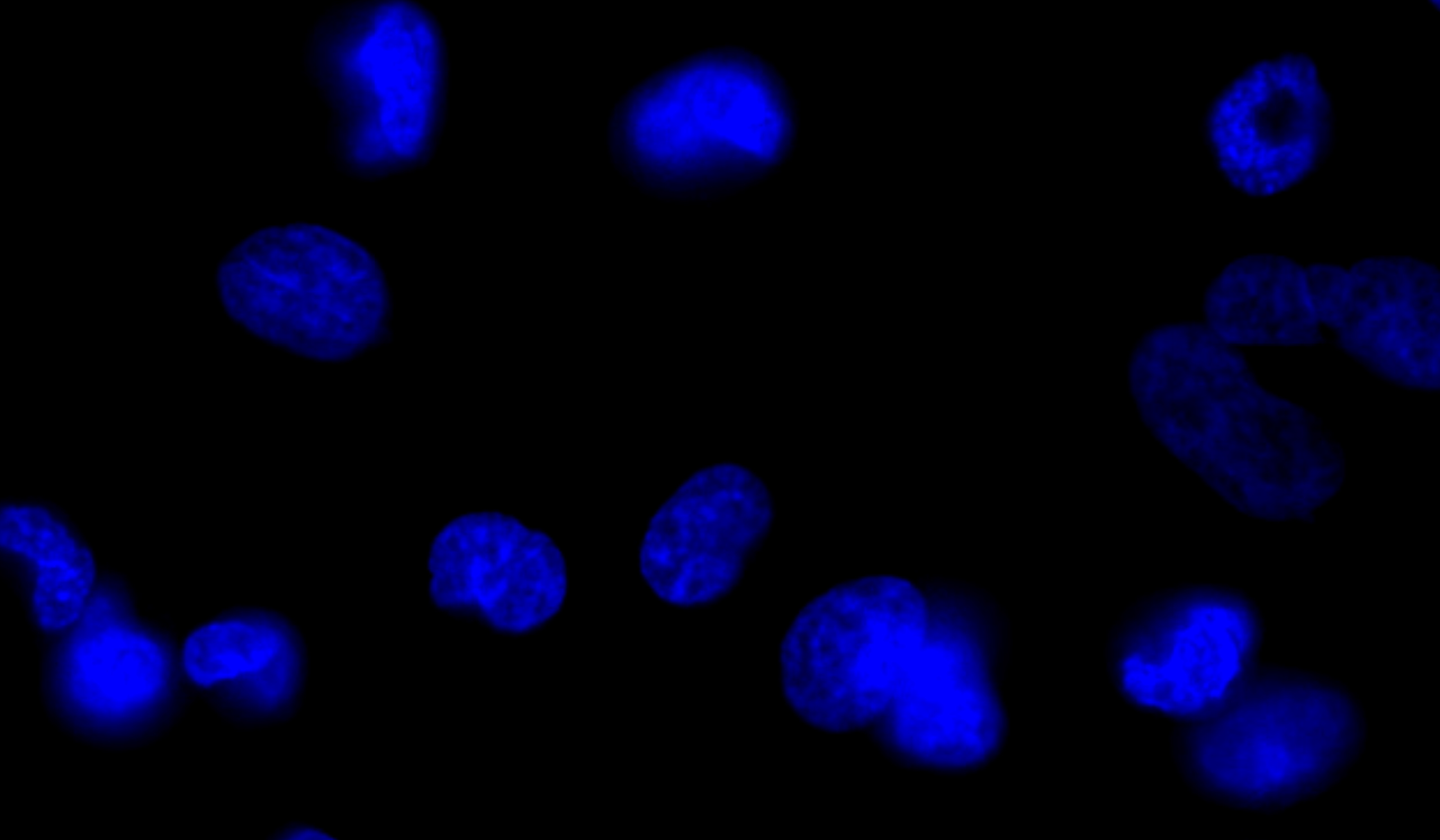

Supplement: Supplemental Information 4 [file peerj-13-20156-s004.zip › 4J/2.jpg]

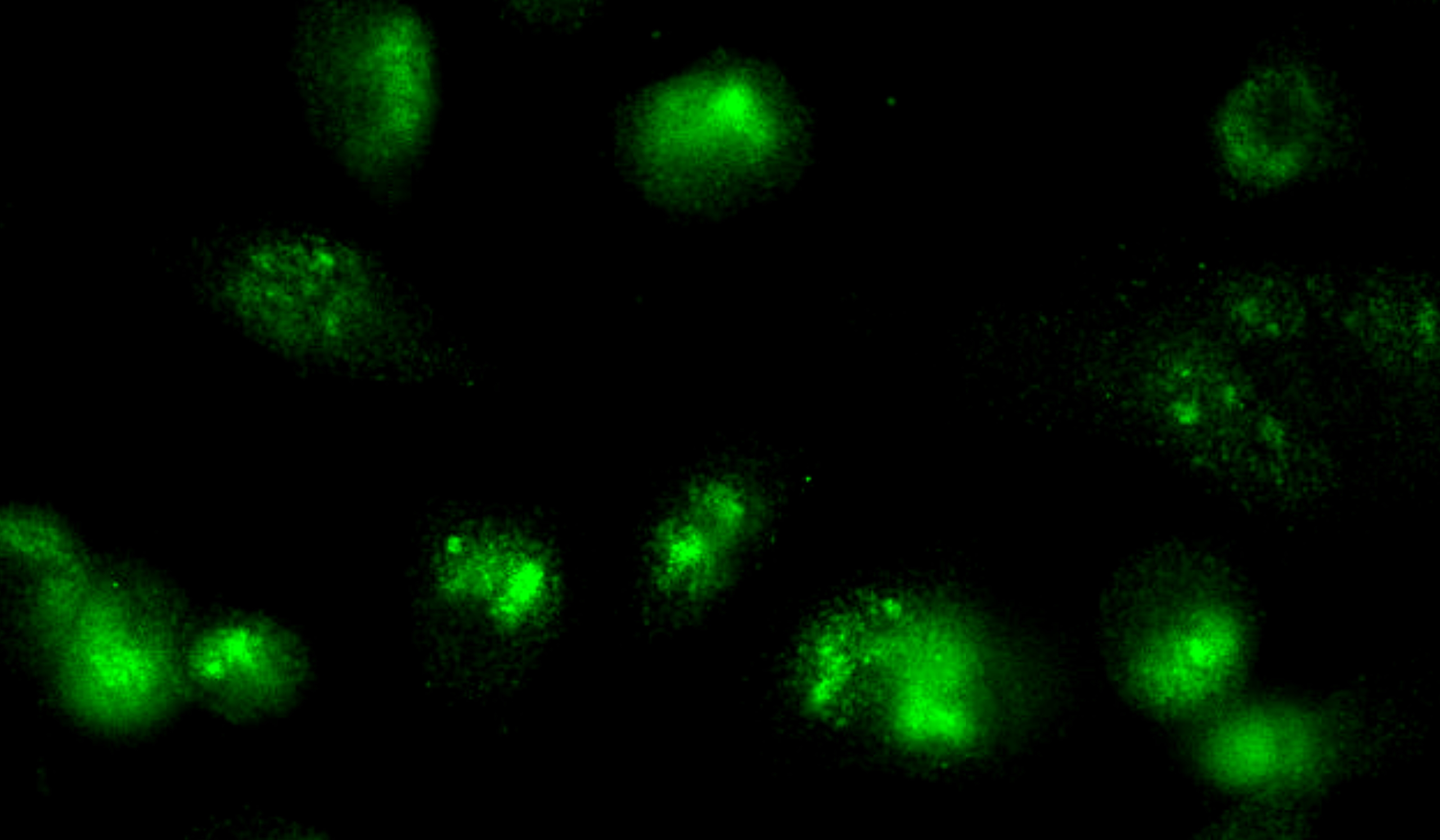

Supplement: Supplemental Information 4 [file peerj-13-20156-s004.zip › 4J/3.jpg]

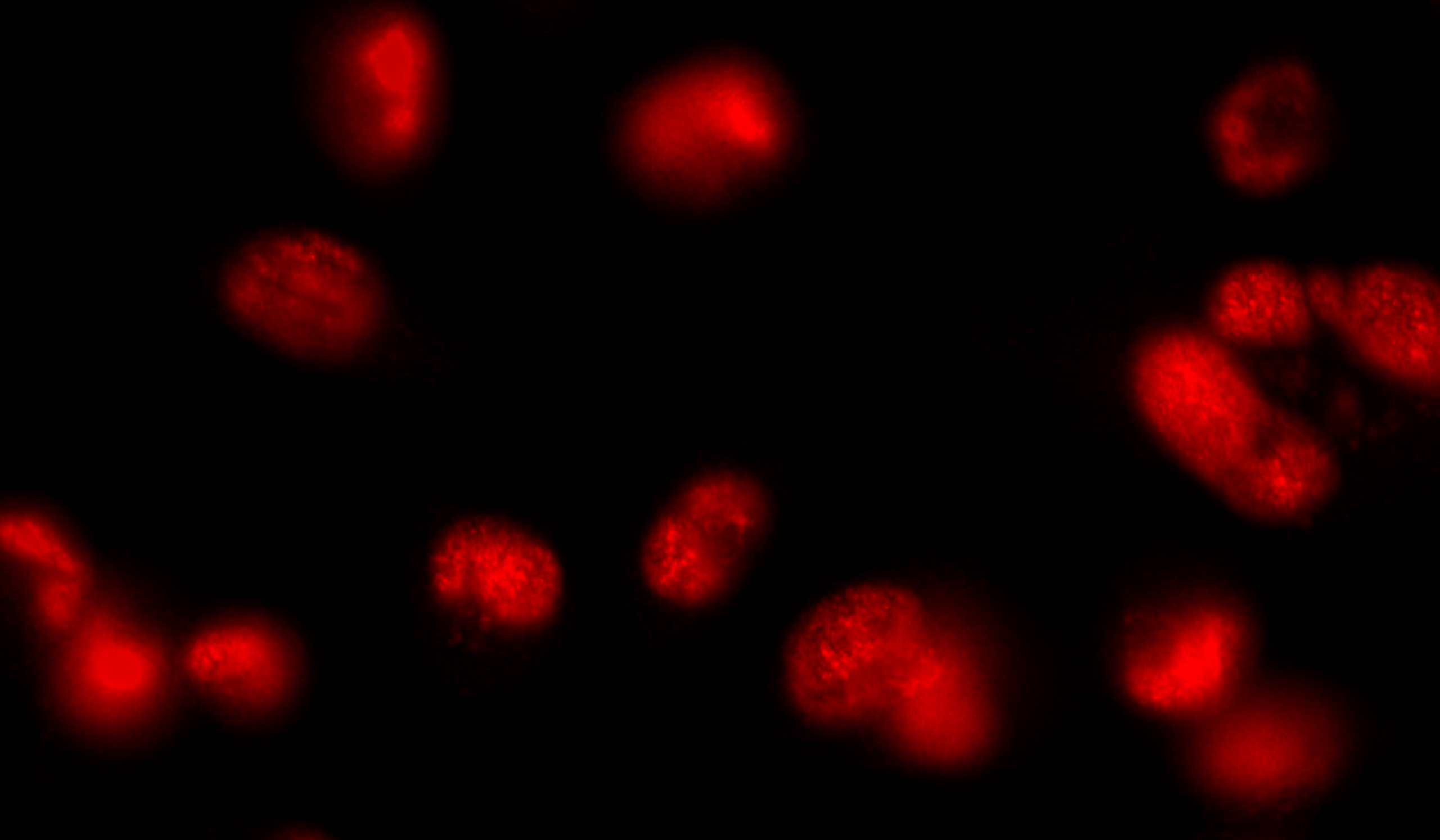

Supplement: Supplemental Information 4 [file peerj-13-20156-s004.zip › 4J/4.jpg]

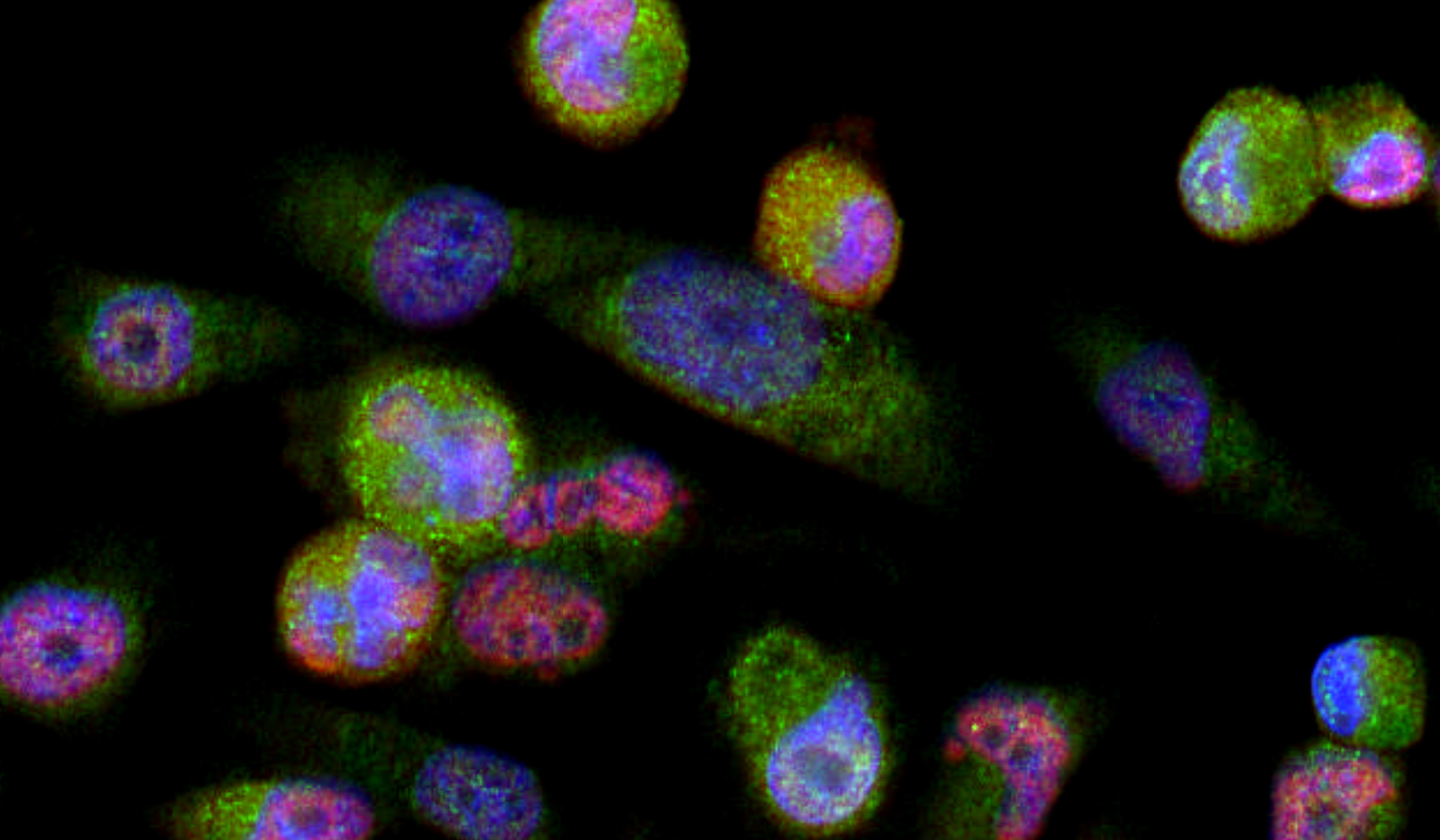

Supplement: Supplemental Information 4 [file peerj-13-20156-s004.zip › 4J/5.jpg]

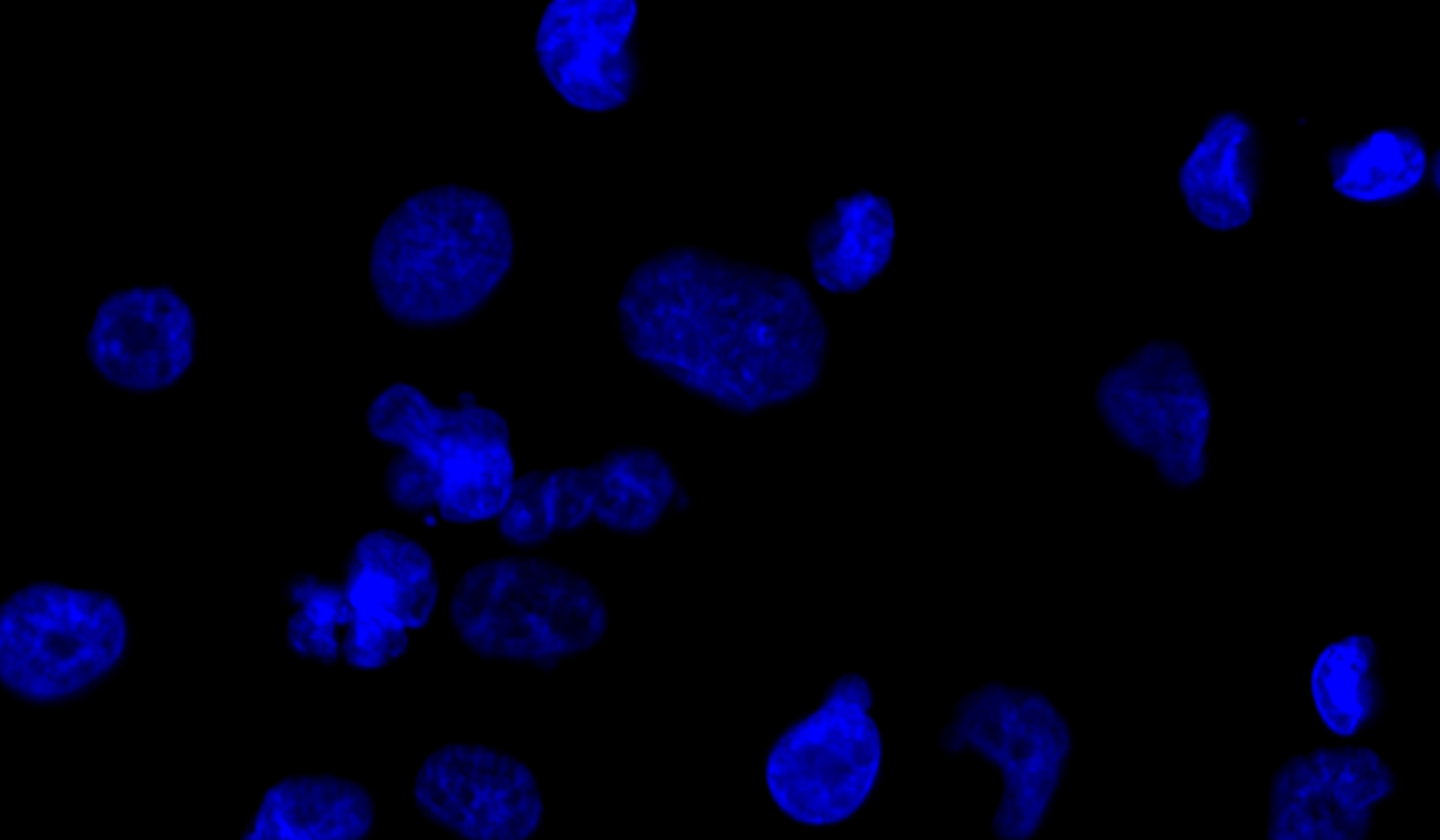

Supplement: Supplemental Information 4 [file peerj-13-20156-s004.zip › 4J/6.jpg]

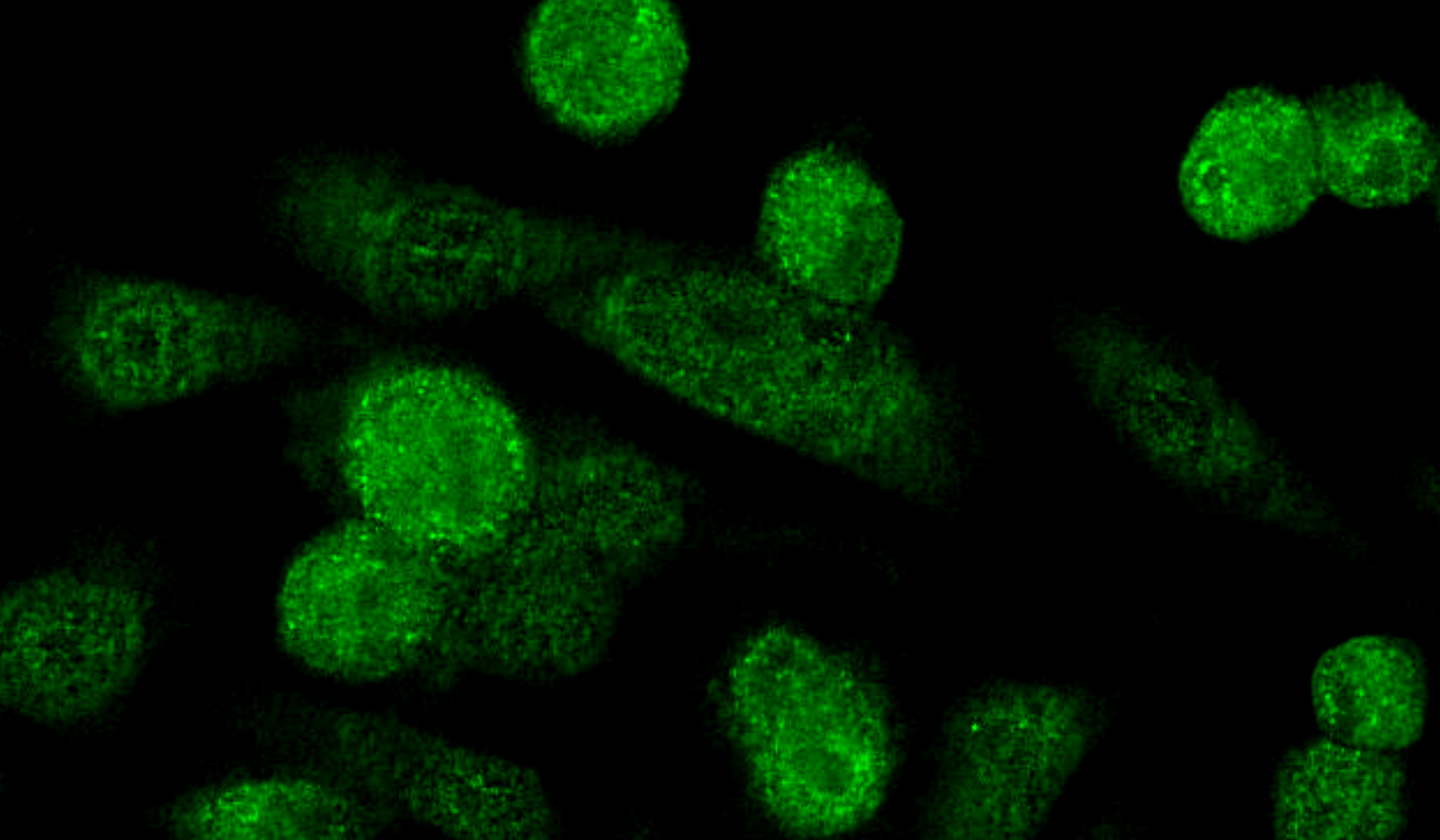

Supplement: Supplemental Information 4 [file peerj-13-20156-s004.zip › 4J/7.jpg]

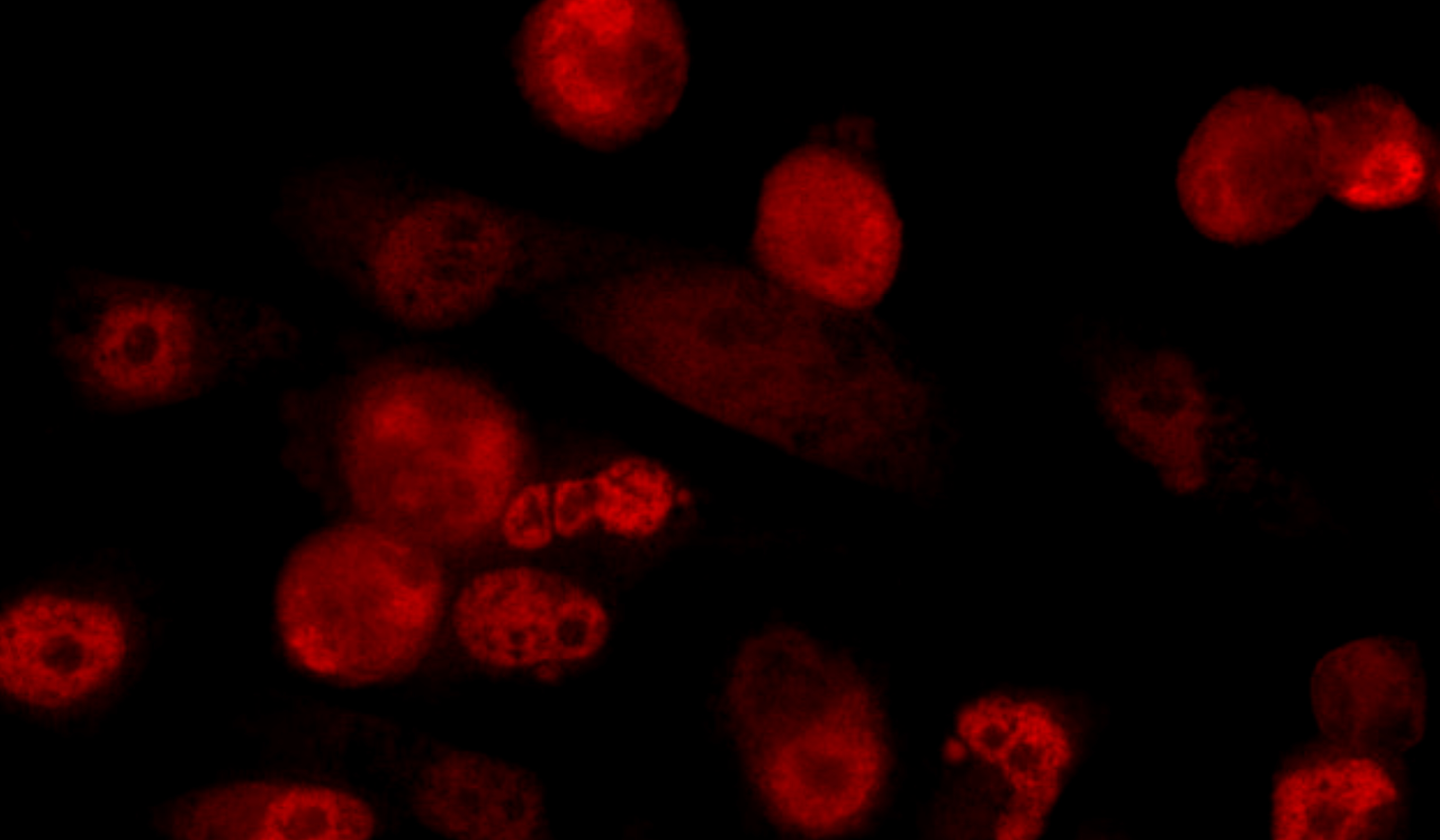

Supplement: Supplemental Information 4 [file peerj-13-20156-s004.zip › 4J/8.jpg]

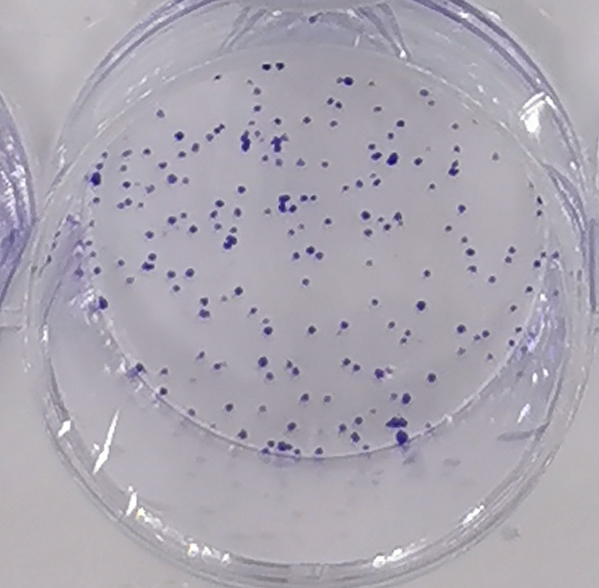

Supplement: Supplemental Information 4 [file peerj-13-20156-s004.zip › 5B/1.jpg]

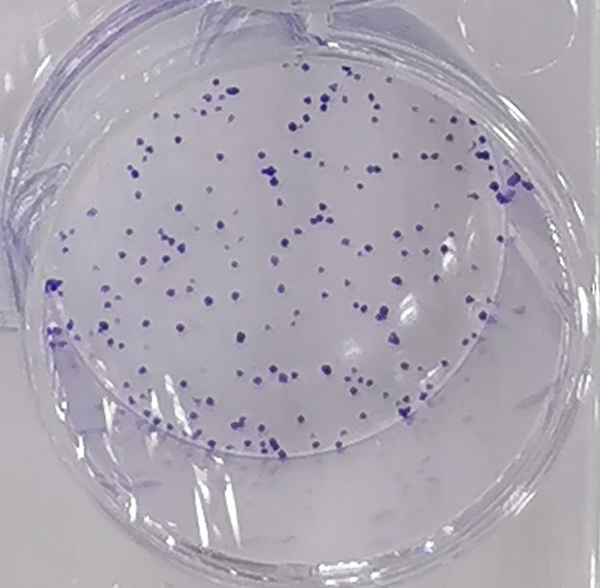

Supplement: Supplemental Information 4 [file peerj-13-20156-s004.zip › 5B/2.jpg]

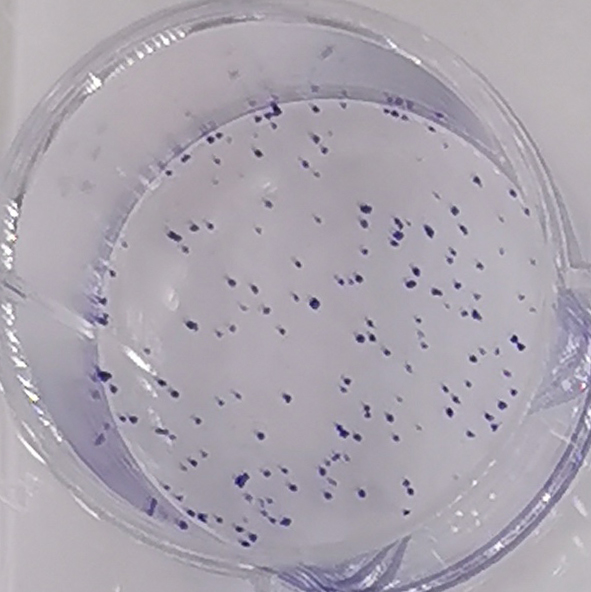

Supplement: Supplemental Information 4 [file peerj-13-20156-s004.zip › 5B/3.jpg]

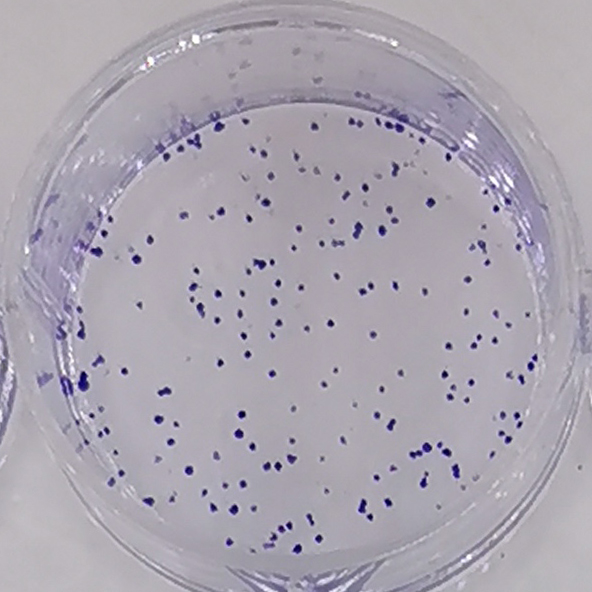

Supplement: Supplemental Information 4 [file peerj-13-20156-s004.zip › 5B/4.jpg]

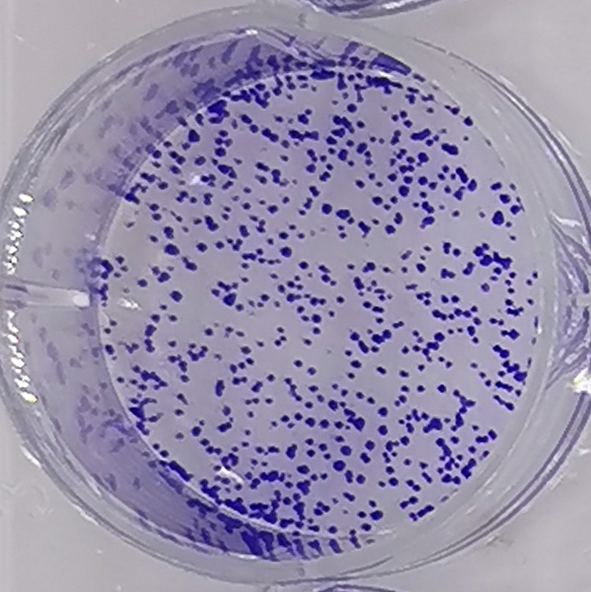

Supplement: Supplemental Information 4 [file peerj-13-20156-s004.zip › 5B/5.jpg]

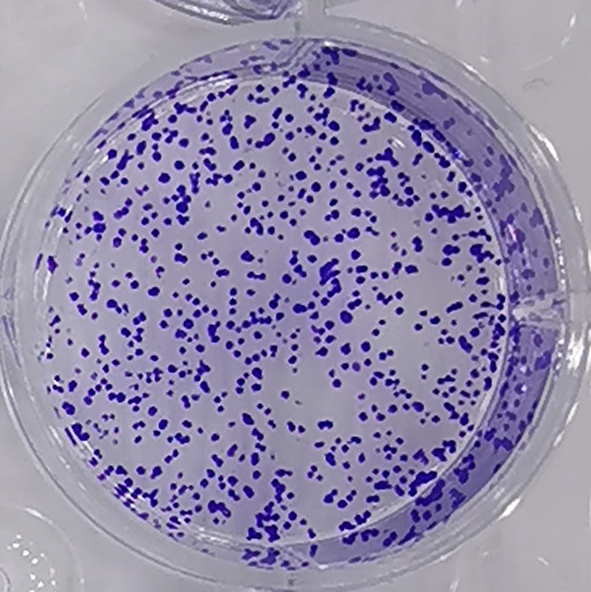

Supplement: Supplemental Information 4 [file peerj-13-20156-s004.zip › 5B/6.jpg]

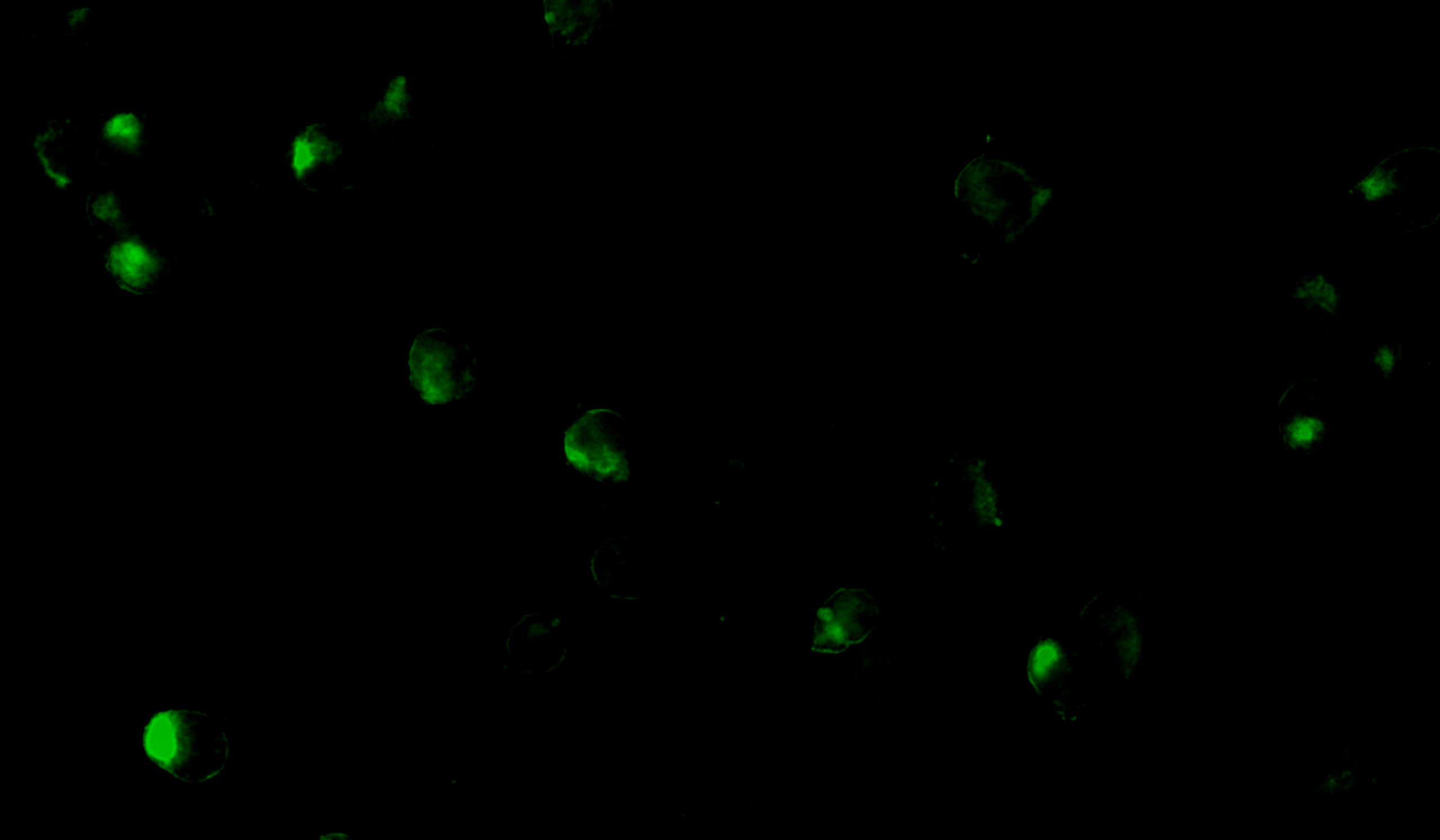

Supplement: Supplemental Information 4 [file peerj-13-20156-s004.zip › 5C/1.jpg]

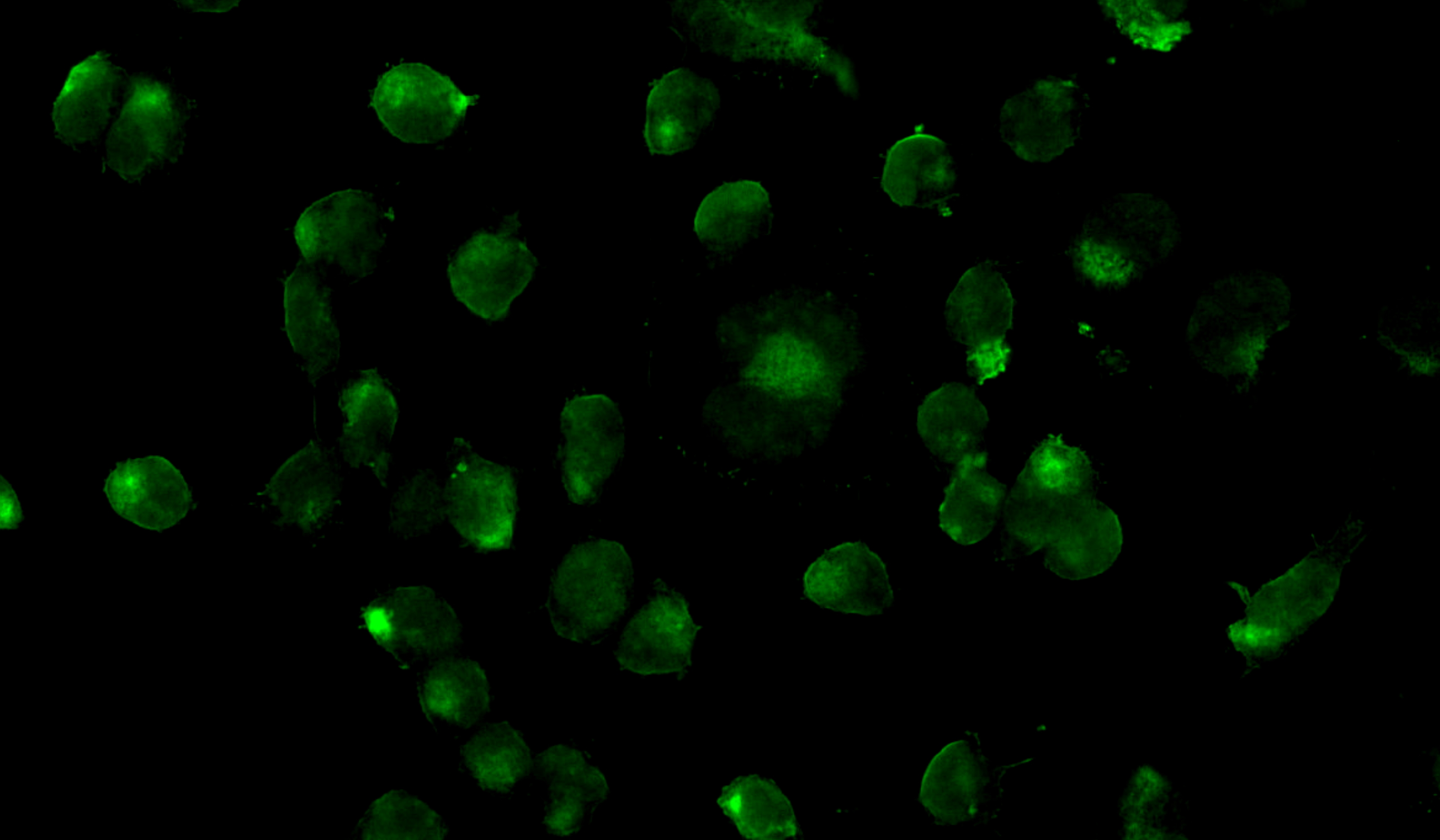

Supplement: Supplemental Information 4 [file peerj-13-20156-s004.zip › 5C/2.jpg]

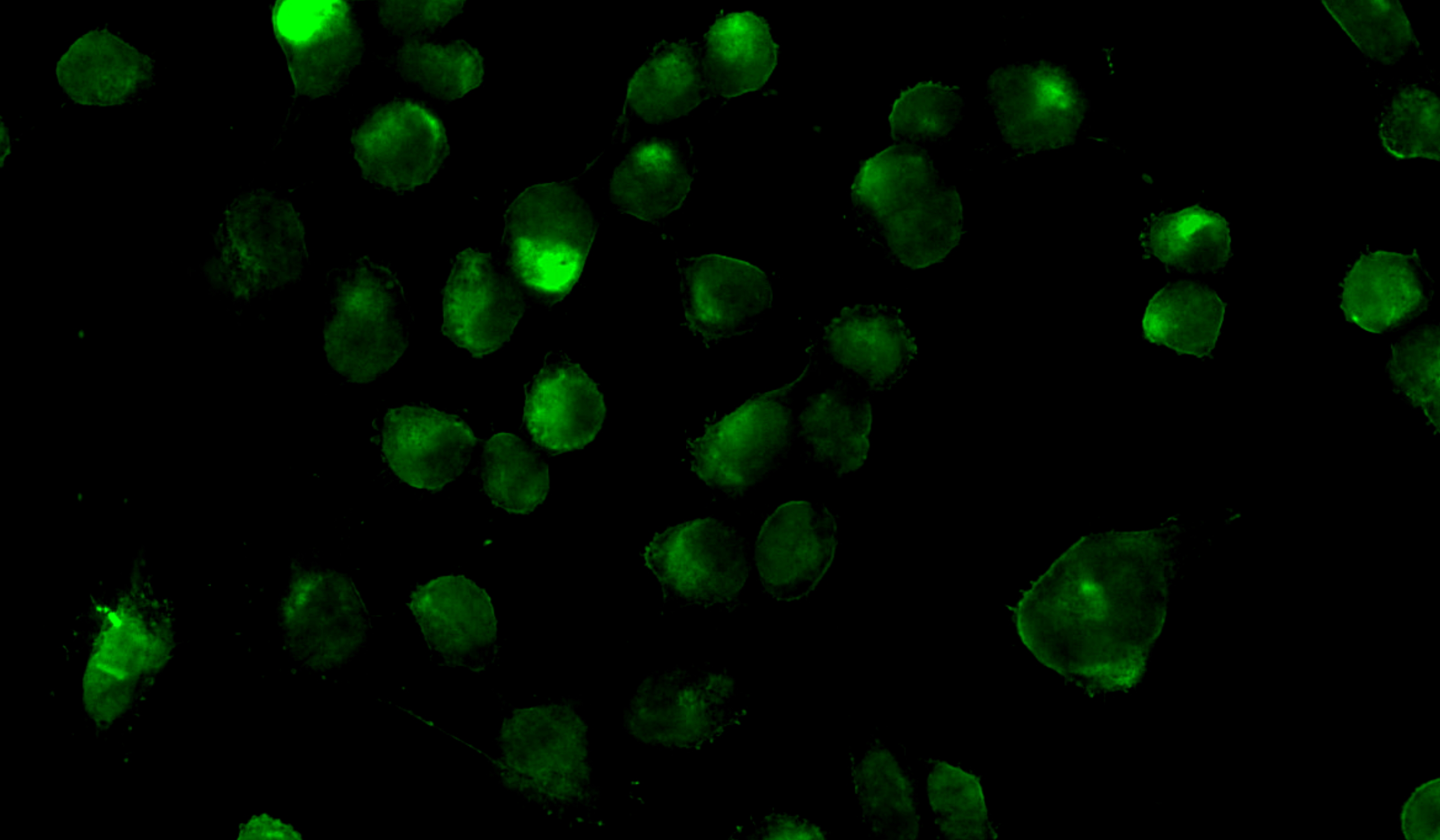

Supplement: Supplemental Information 4 [file peerj-13-20156-s004.zip › 5C/3.jpg]

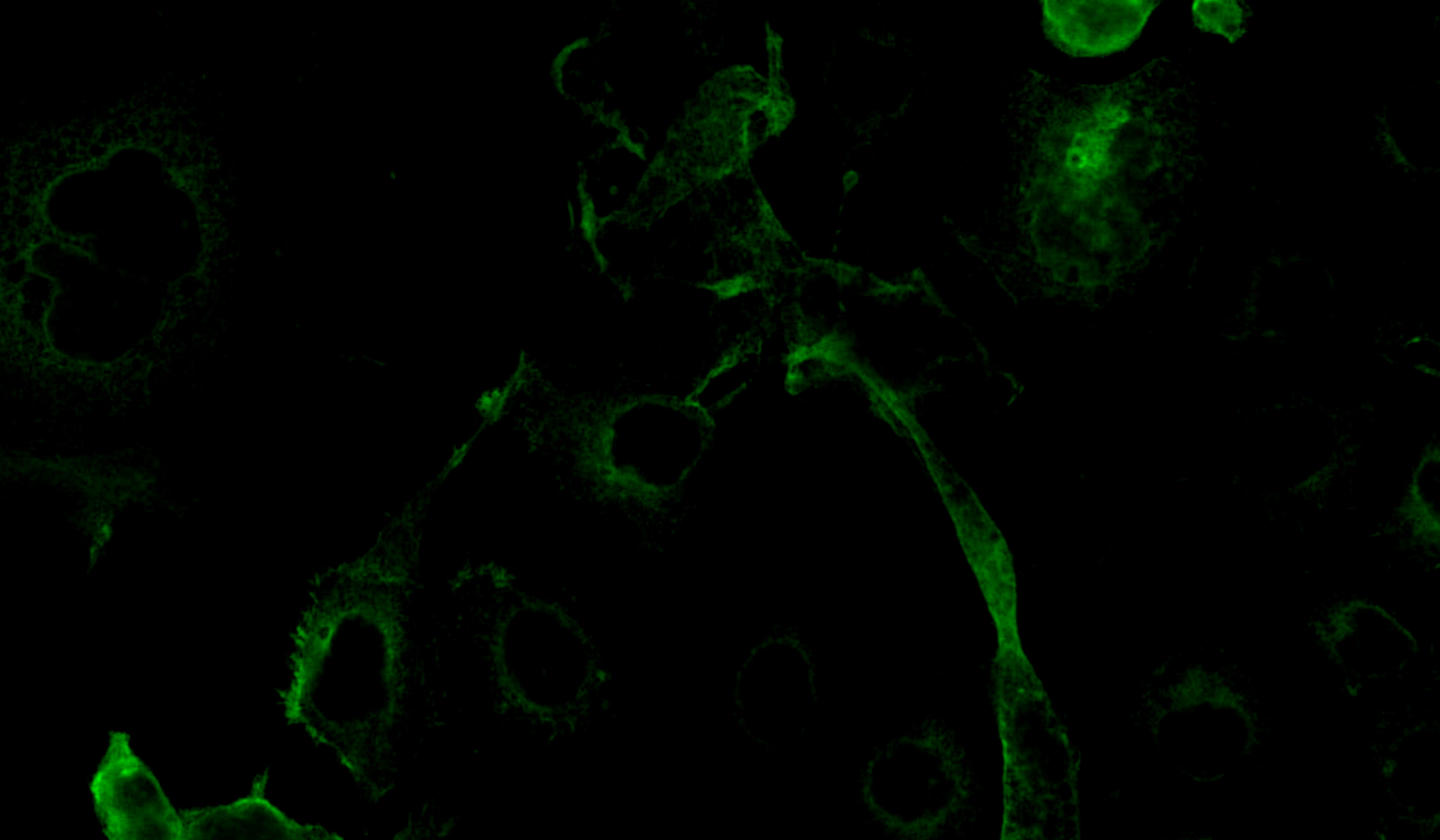

Supplement: Supplemental Information 4 [file peerj-13-20156-s004.zip › 5C/4.jpg]

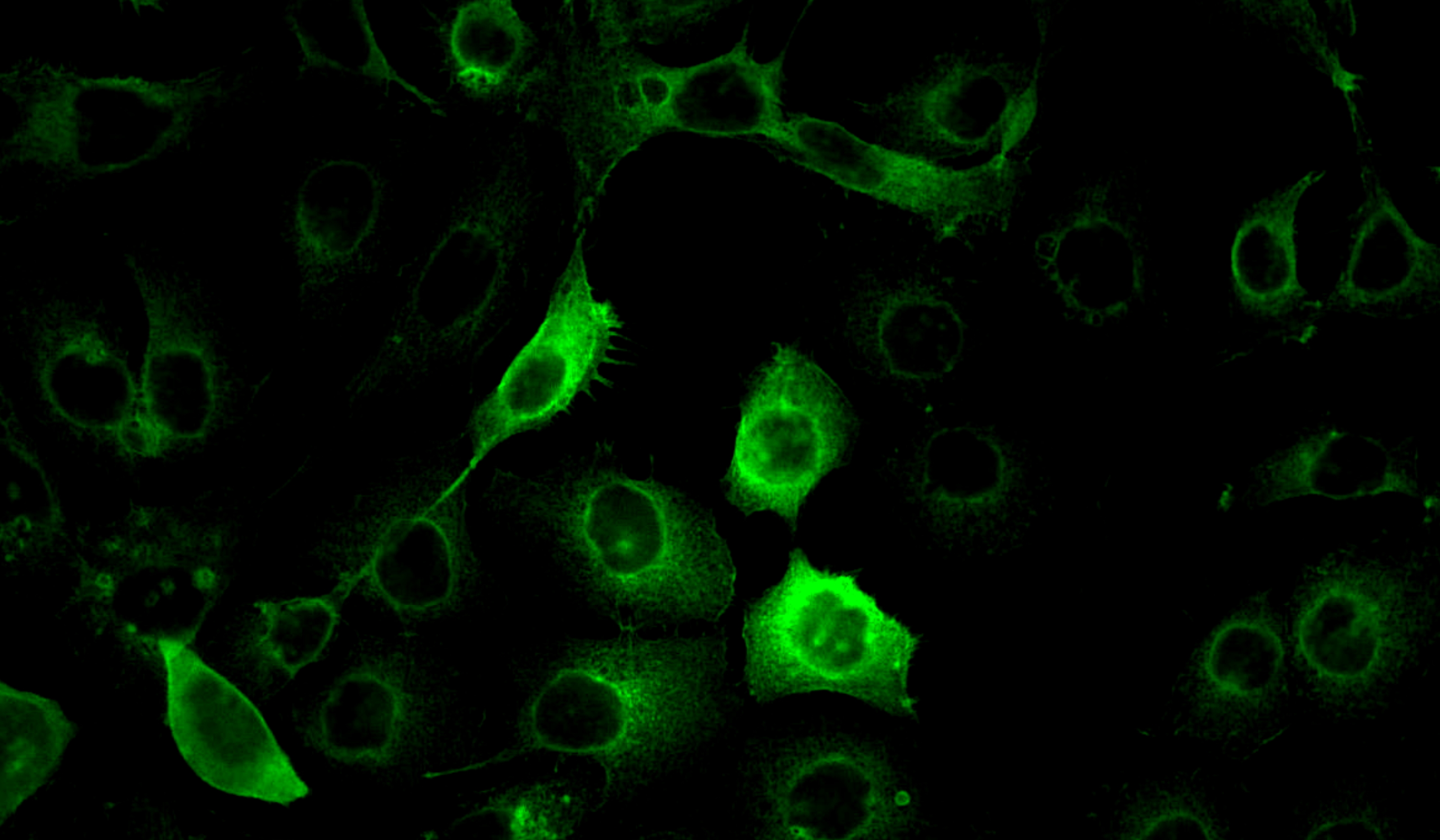

Supplement: Supplemental Information 4 [file peerj-13-20156-s004.zip › 5C/5.jpg]

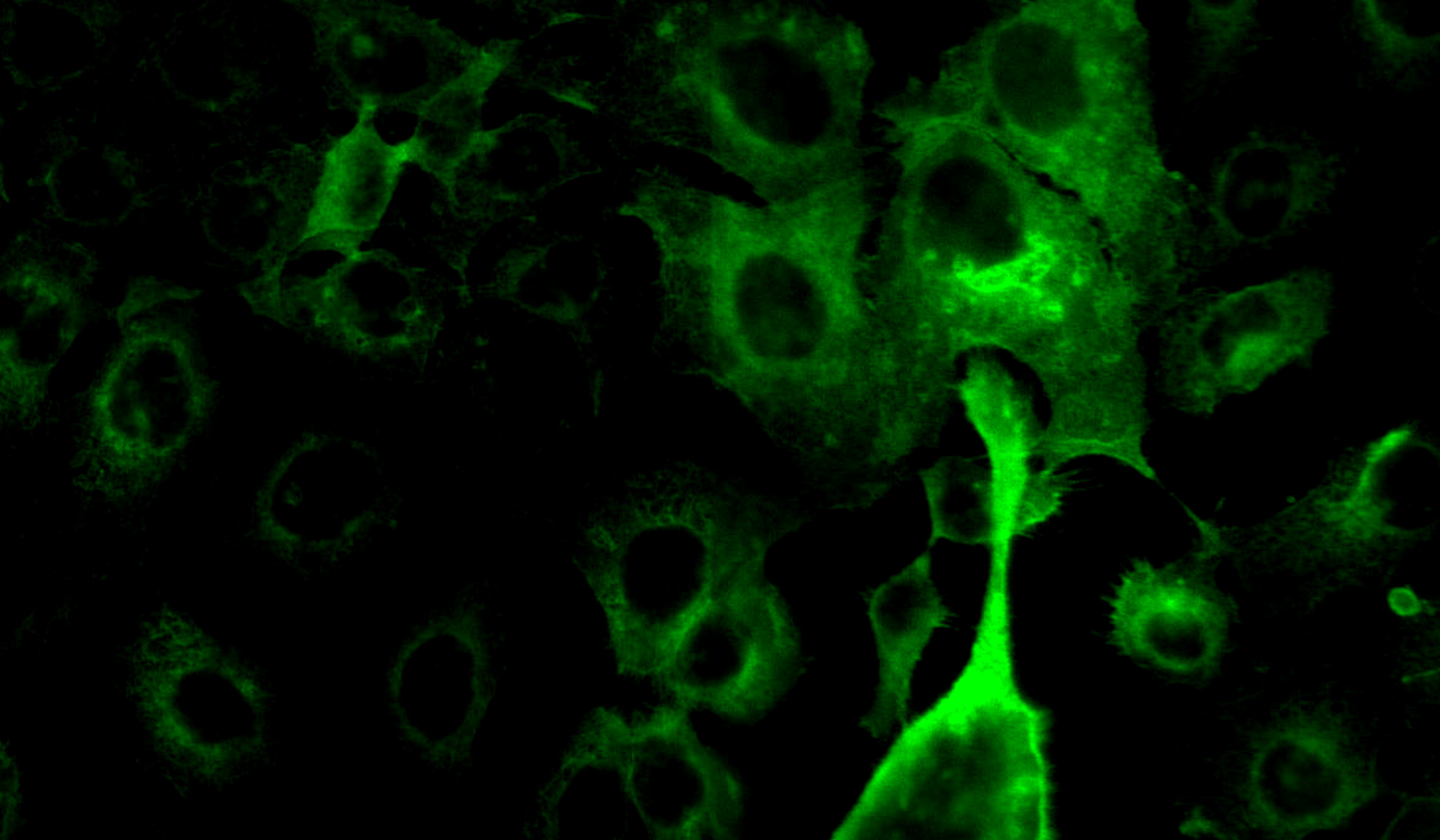

Supplement: Supplemental Information 4 [file peerj-13-20156-s004.zip › 5C/6.jpg]

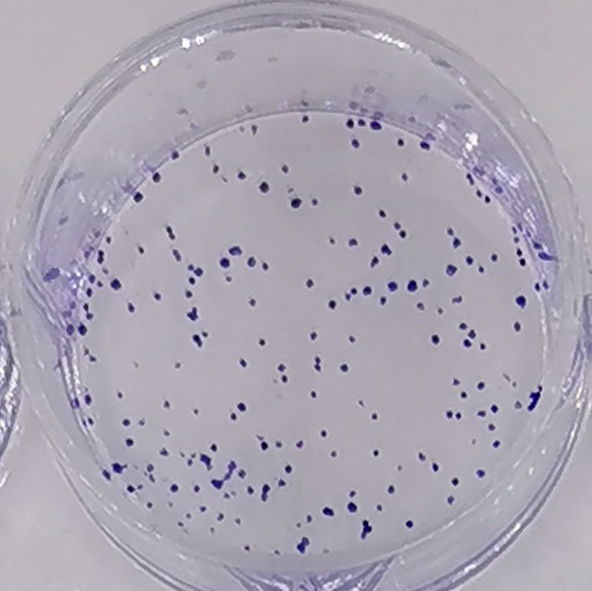

Supplement: Supplemental Information 4 [file peerj-13-20156-s004.zip › 6D/1.jpg]

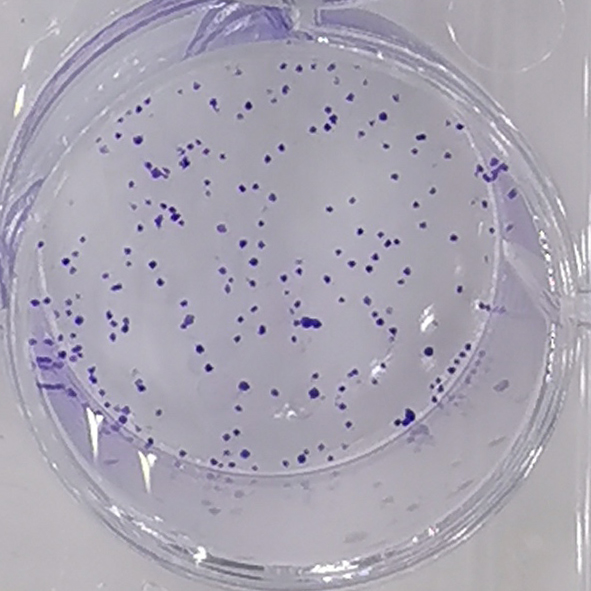

Supplement: Supplemental Information 4 [file peerj-13-20156-s004.zip › 6D/2.jpg]

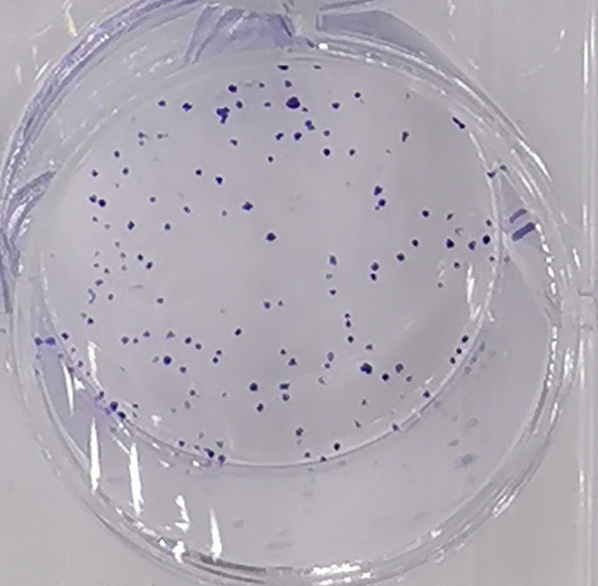

Supplement: Supplemental Information 4 [file peerj-13-20156-s004.zip › 6D/3.jpg]

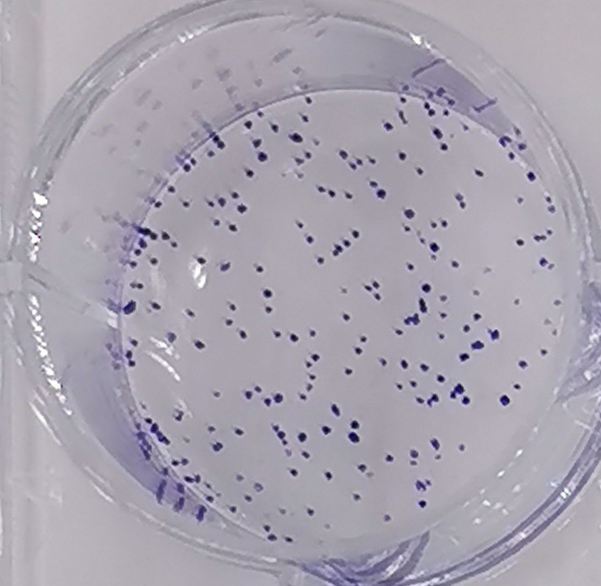

Supplement: Supplemental Information 4 [file peerj-13-20156-s004.zip › 6D/4.jpg]

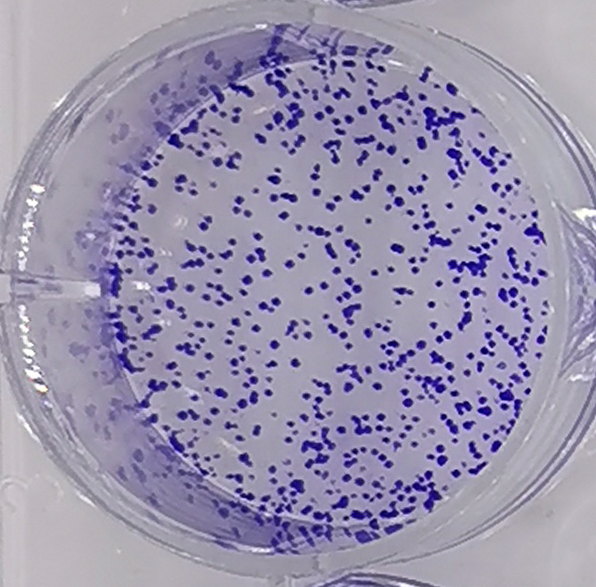

Supplement: Supplemental Information 4 [file peerj-13-20156-s004.zip › 6D/5.jpg]

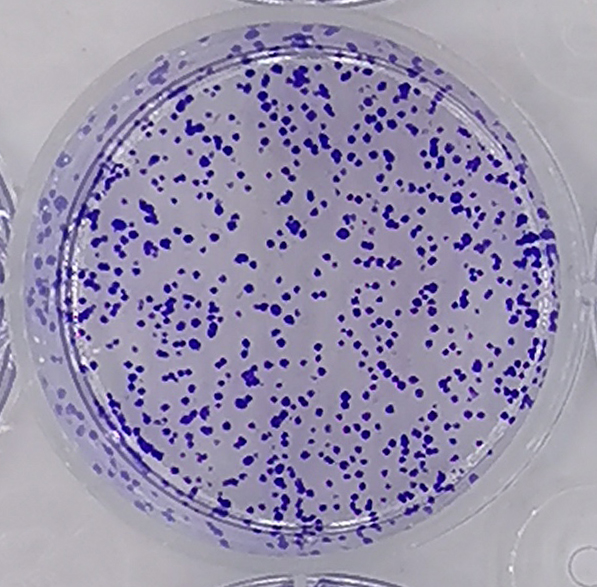

Supplement: Supplemental Information 4 [file peerj-13-20156-s004.zip › 6D/6.jpg]

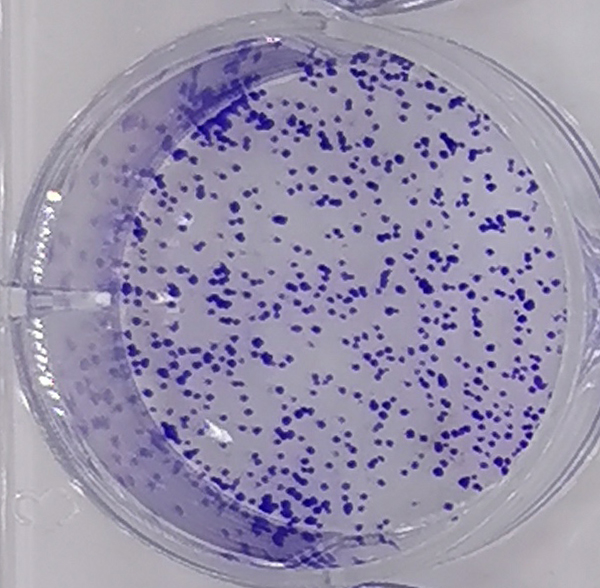

Supplement: Supplemental Information 4 [file peerj-13-20156-s004.zip › 6D/7.jpg]

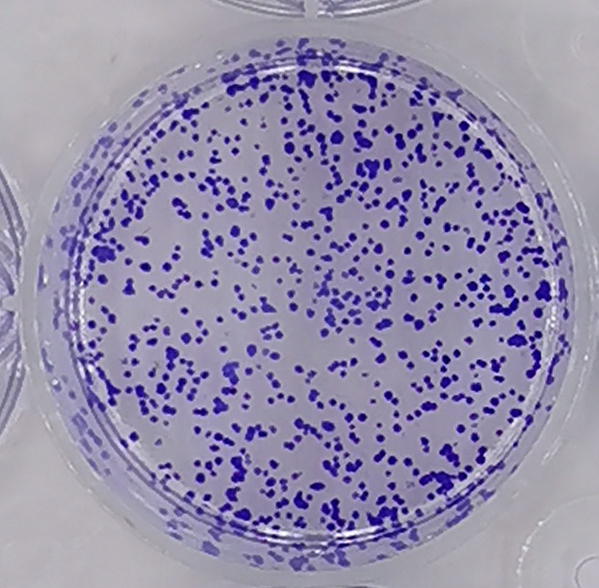

Supplement: Supplemental Information 4 [file peerj-13-20156-s004.zip › 6D/8.jpg]

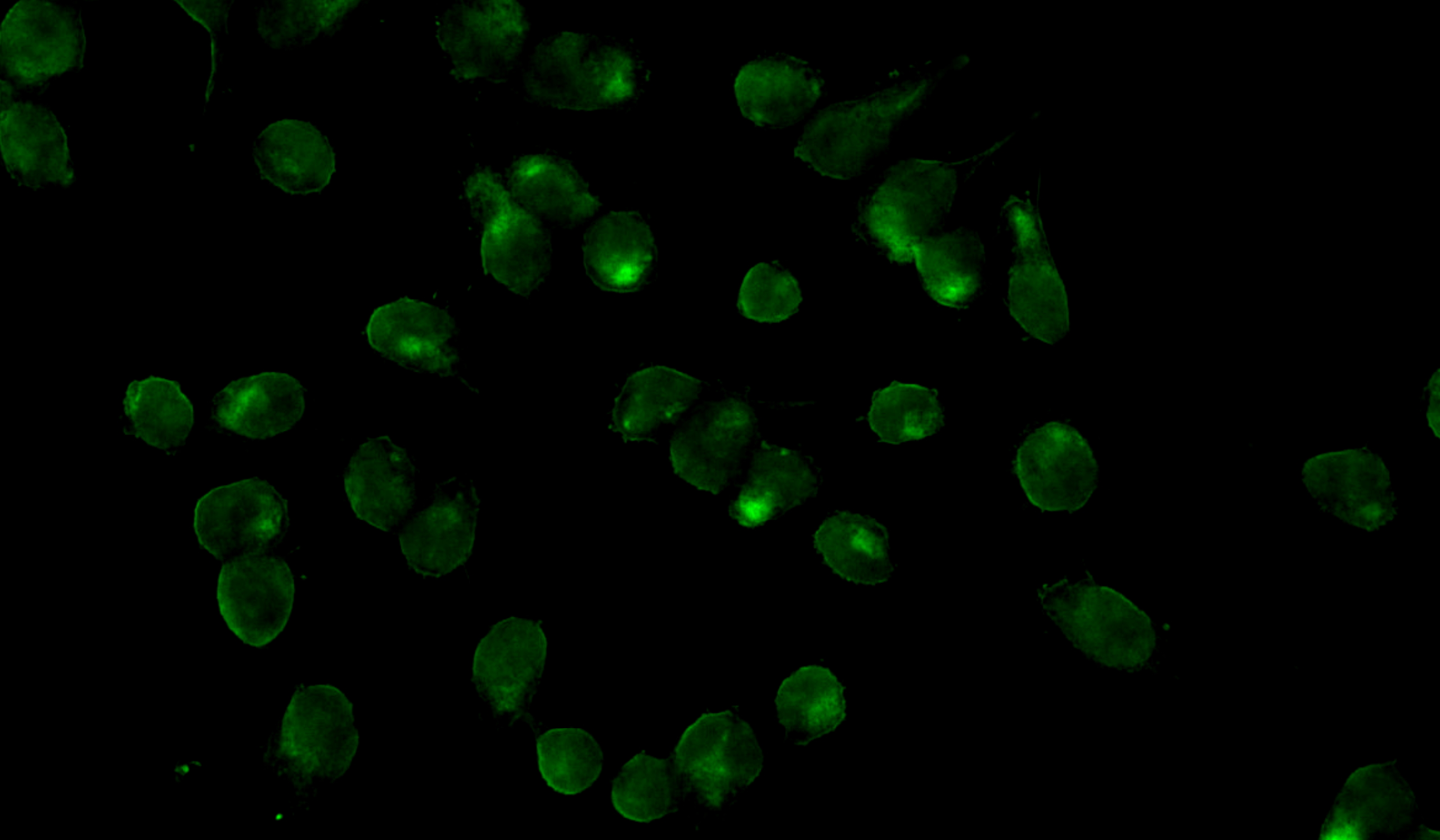

Supplement: Supplemental Information 4 [file peerj-13-20156-s004.zip › 6G/1.jpg]

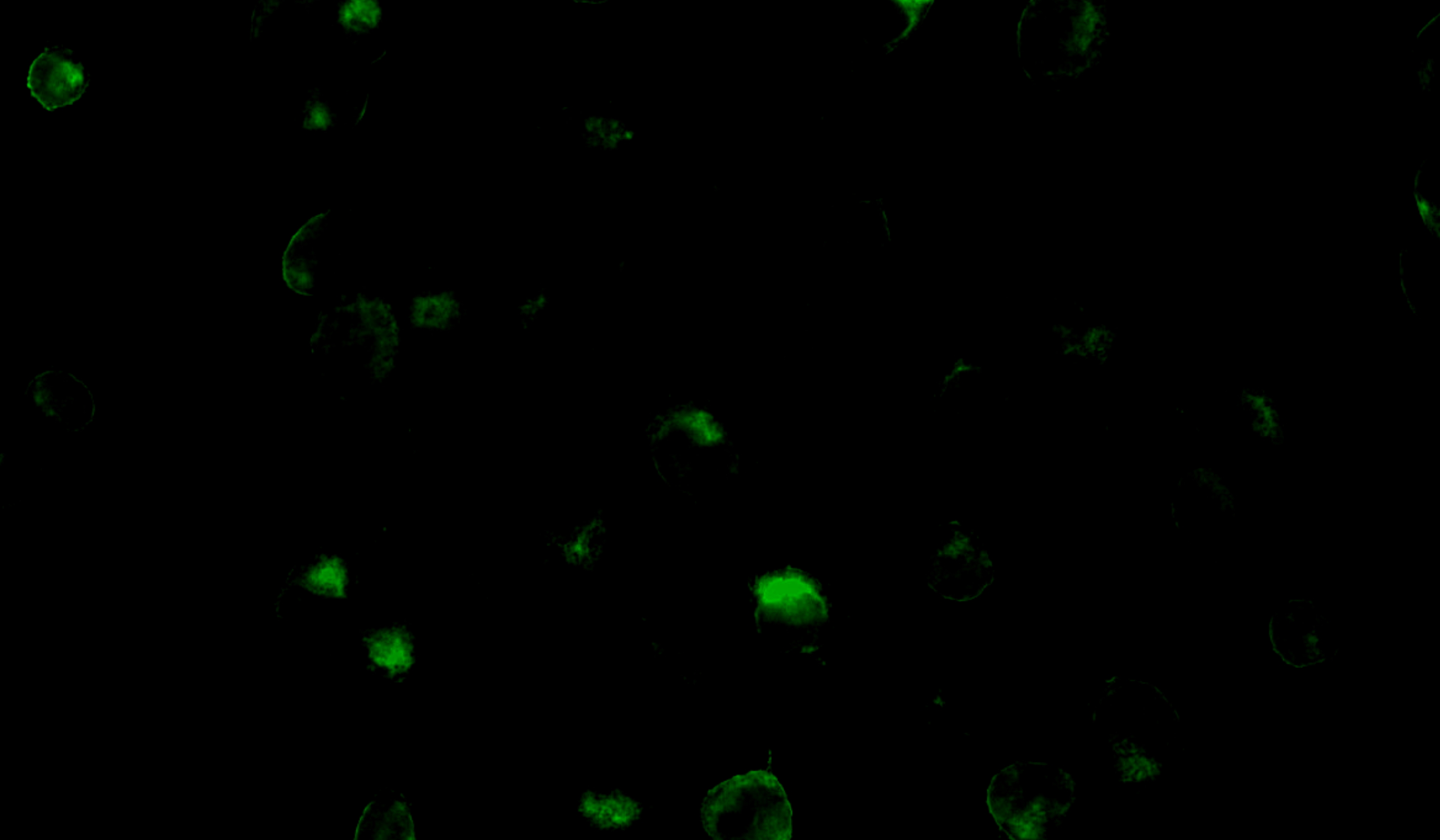

Supplement: Supplemental Information 4 [file peerj-13-20156-s004.zip › 6G/2.jpg]

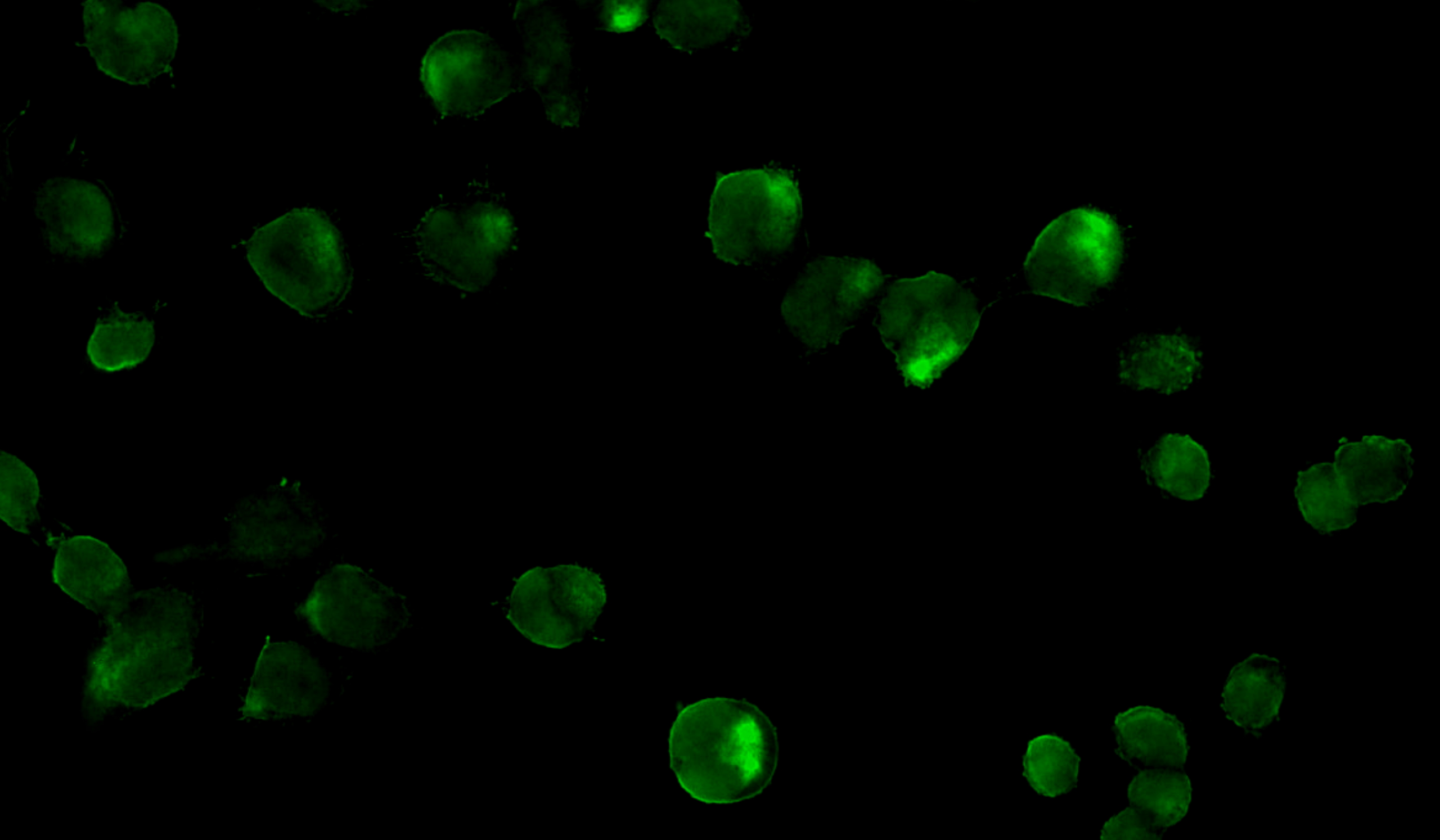

Supplement: Supplemental Information 4 [file peerj-13-20156-s004.zip › 6G/3.jpg]

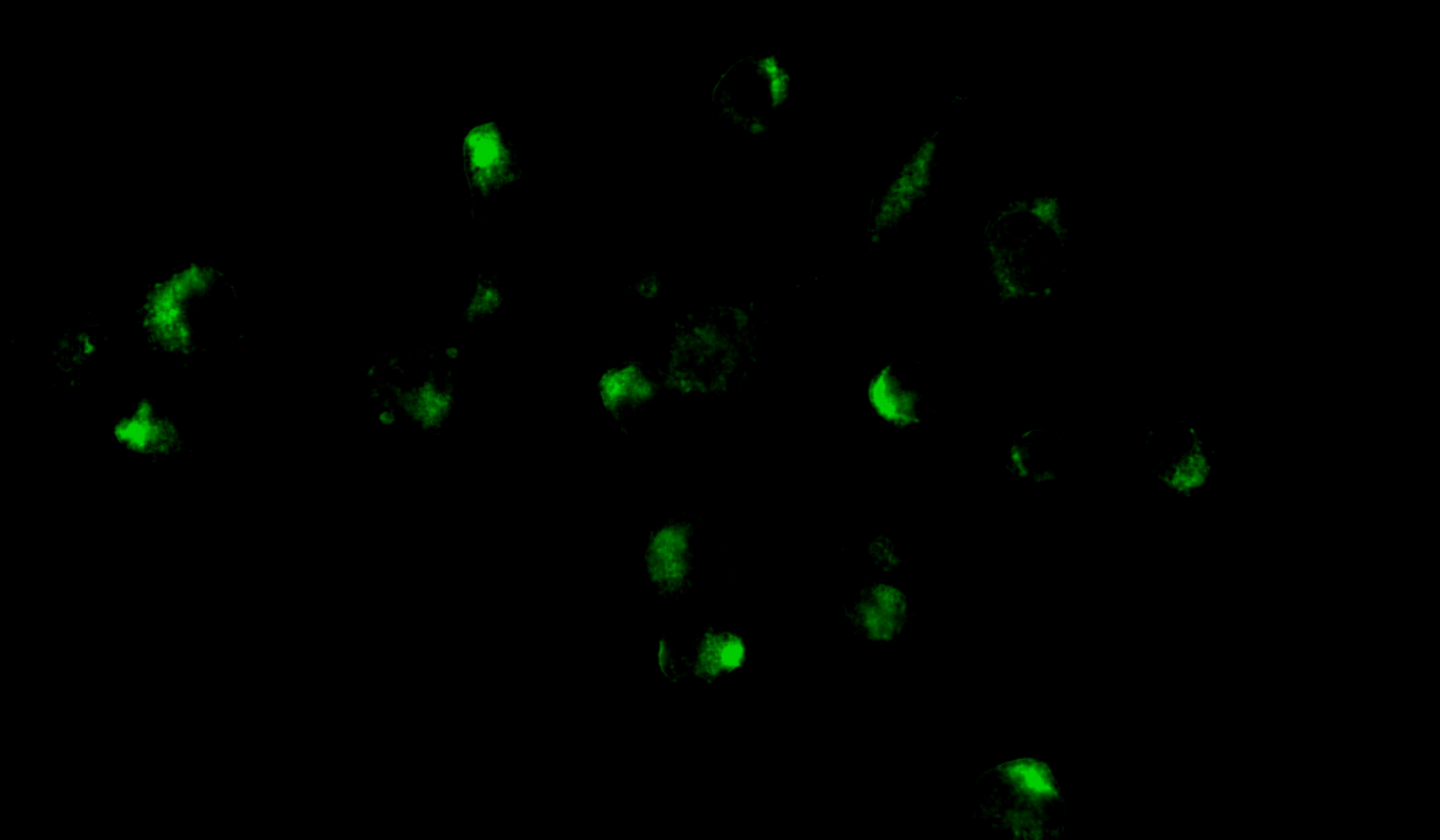

Supplement: Supplemental Information 4 [file peerj-13-20156-s004.zip › 6G/4.jpg]

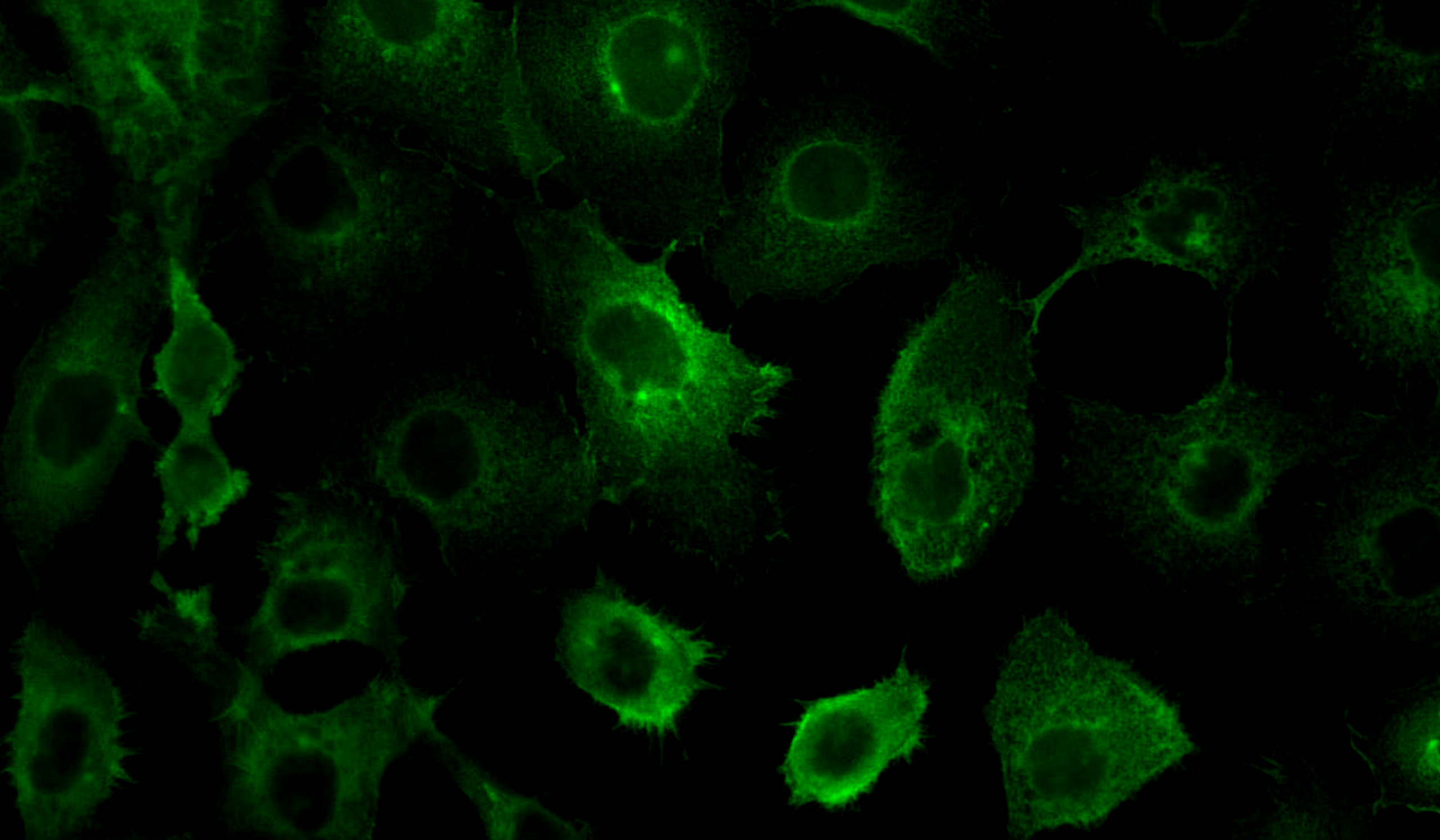

Supplement: Supplemental Information 4 [file peerj-13-20156-s004.zip › 6G/5.jpg]

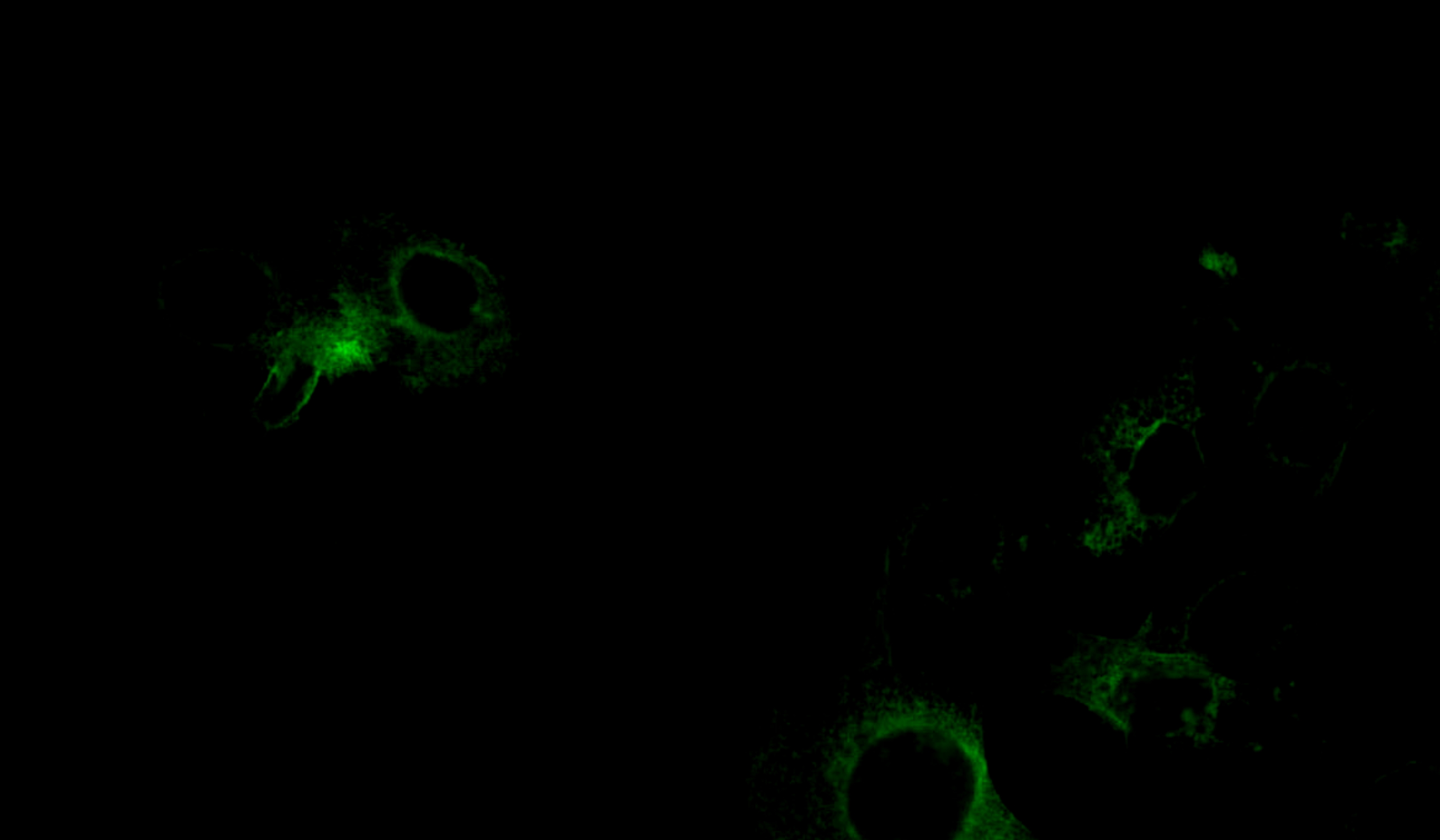

Supplement: Supplemental Information 4 [file peerj-13-20156-s004.zip › 6G/6.jpg]

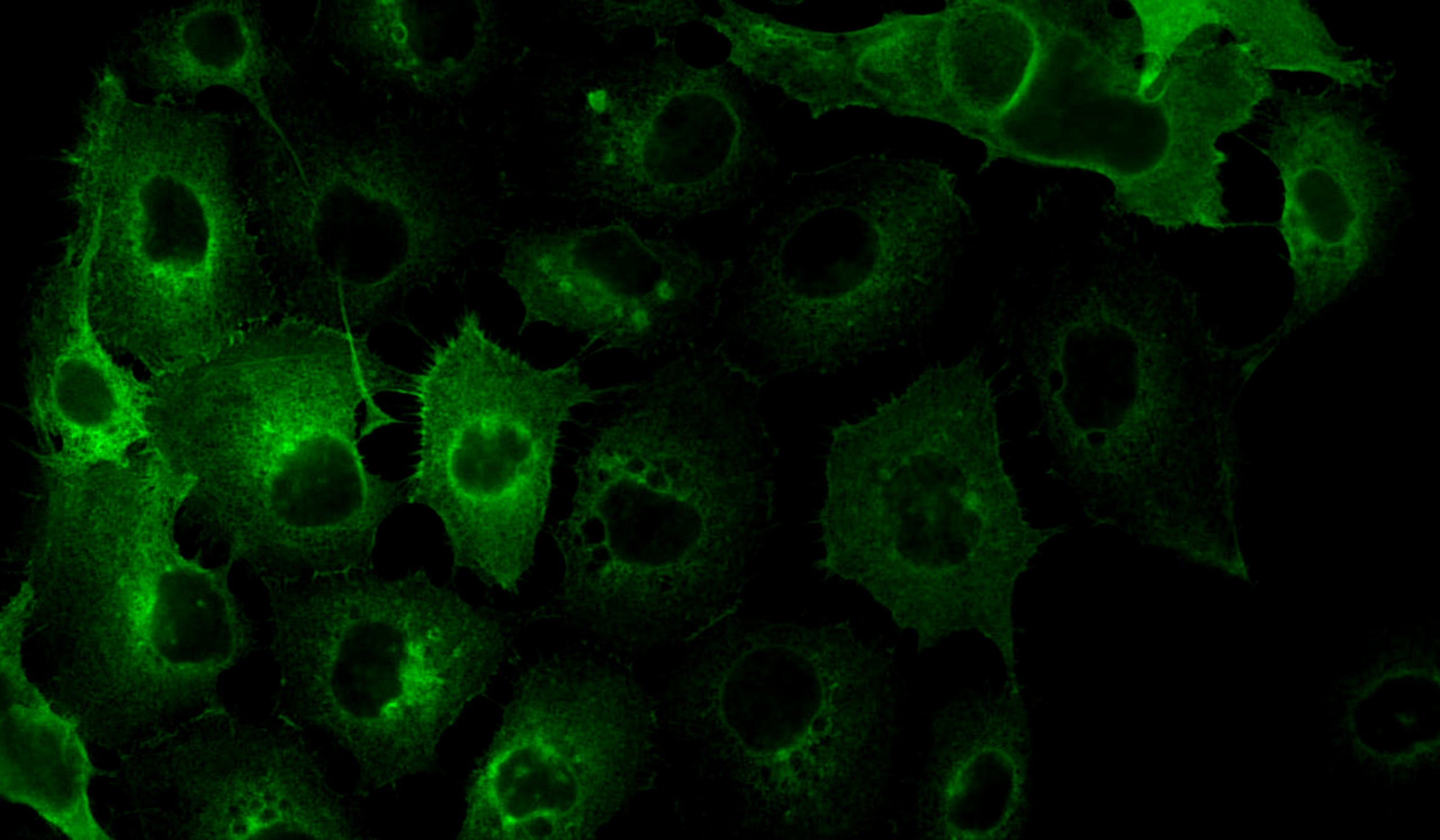

Supplement: Supplemental Information 4 [file peerj-13-20156-s004.zip › 6G/7.jpg]

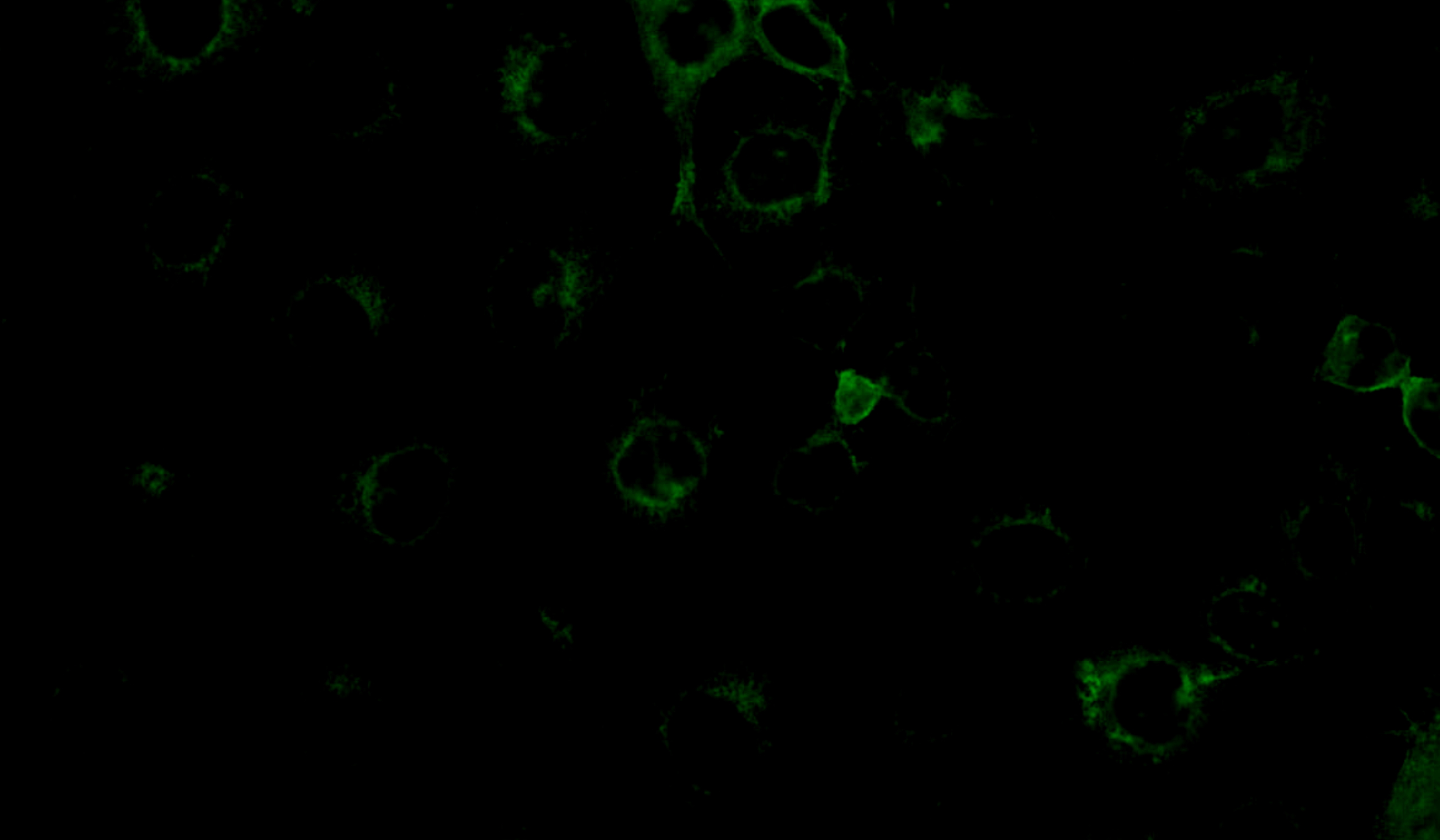

Supplement: Supplemental Information 4 [file peerj-13-20156-s004.zip › 6G/8.jpg]

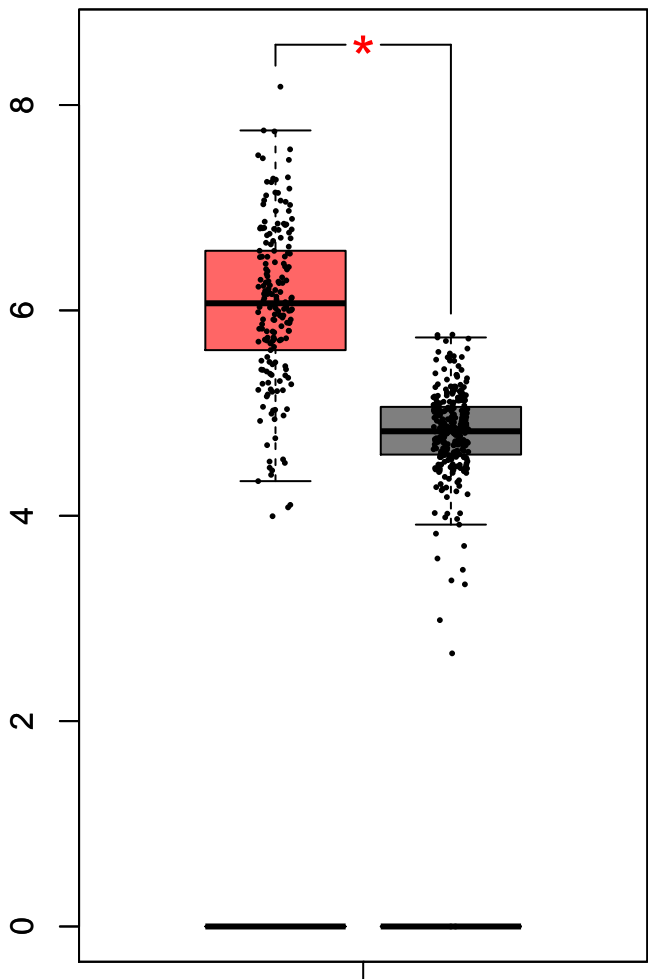

ESCA  
(num(T)=182; num(N)=286)

Supplement: Supplemental Information 4 [file peerj-13-20156-s004.zip › 1B.pdf]

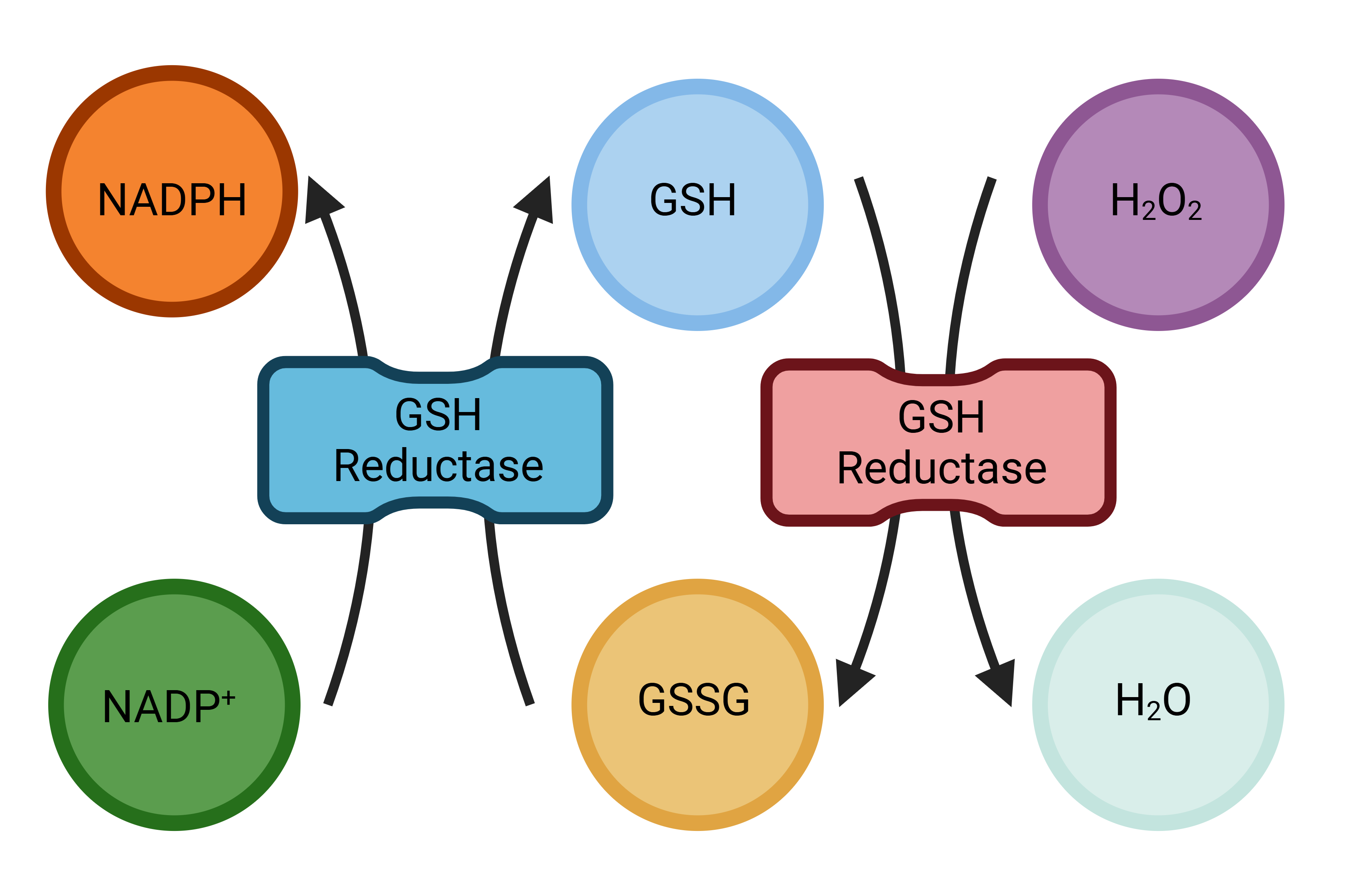

Supplement: Supplemental Information 4 [file peerj-13-20156-s004.zip › 2B.png]

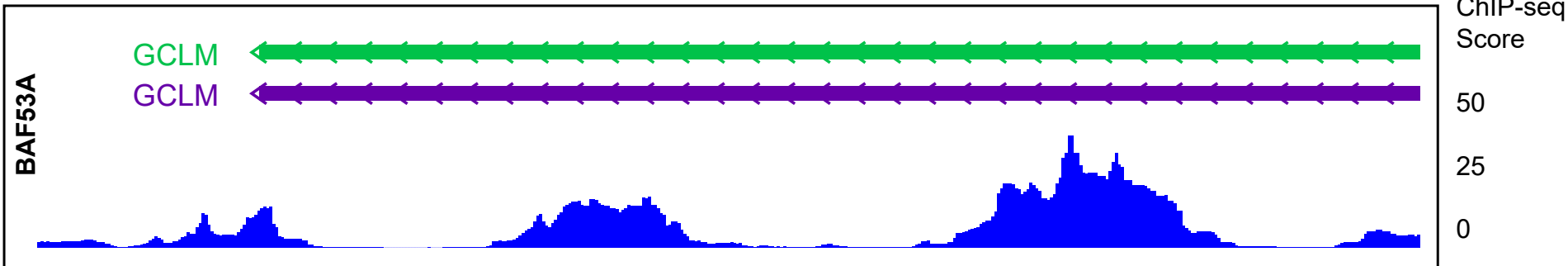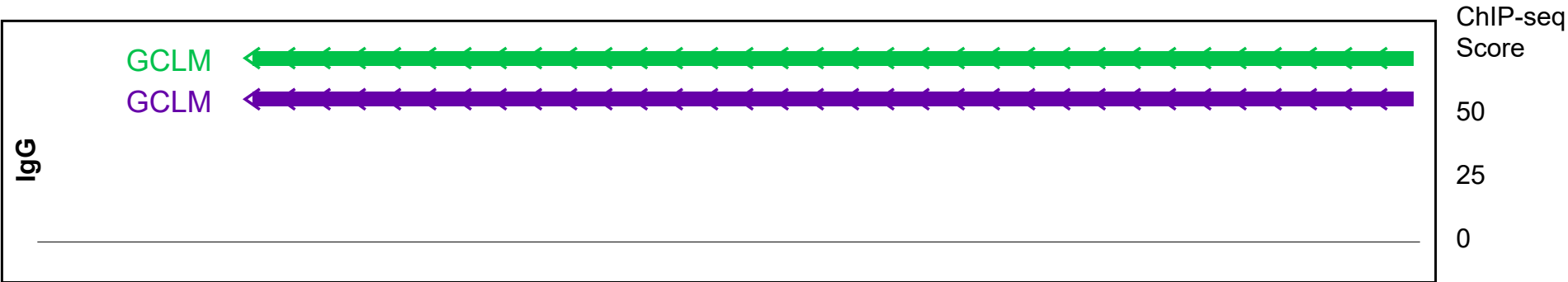

Supplement: Supplemental Information 4 [file peerj-13-20156-s004.zip › 4C-1.pdf]

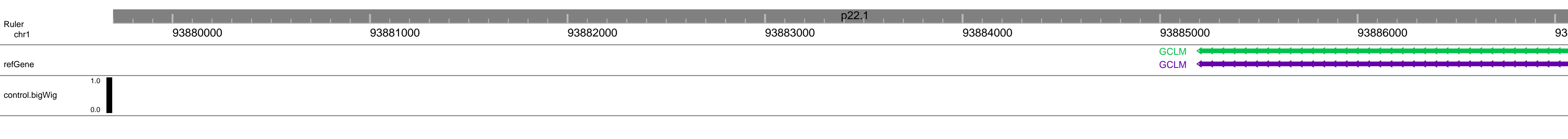

Supplement: Supplemental Information 4 [file peerj-13-20156-s004.zip › 4C-2.pdf]
