# Supplementary material for: The BAF53A-BACH1-GCLM axis regulates glutathione metabolism and enhances ferroptosis resistance in esophageal squamous cell carcinoma
Source: PeerJ. 2025 Oct 3;13:e20156. doi: 10.7717/peerj.20156 (PMC12499559; doi:10.7717/peerj.20156)
Supplement: Supplemental Information 4 [file peerj-13-20156-s004.zip › 6I1.pdf]

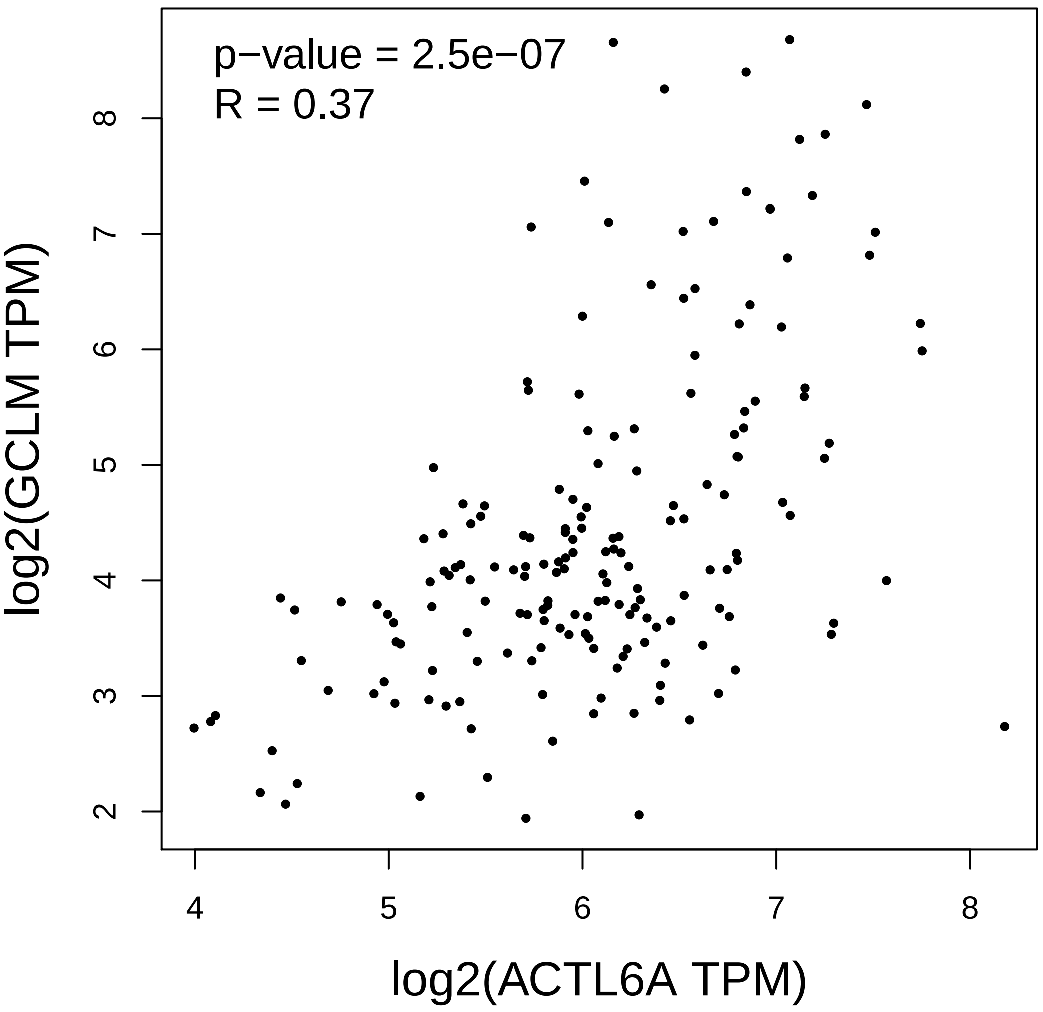
p−value = 2.5e−07
R = 0.37
log2(GCLM TPM)
log2(ACTL6A TPM)
2
3
4
5
6
7
8
4
5
6
7
8
